# Cold-induced vasodilation: A meta-analysis

**DOI:** 10.1080/23328940.2026.2646391

**Published:** 2026-04-14

**Authors:** Rebecca S. Weller, Jacek Buczny, Hein A.M. Daanen

**Affiliations:** Department of Human Movement Sciences, Vrije Universiteit Amsterdam, Amsterdam, Netherlands

**Keywords:** CIVD, finger skin temperature, hunting reaction, cold-water immersion, peripheral circulation, literature review, finger blood flow, arteriovenous anastomoses

## Abstract

Nearly a century ago, cold-induced vasodilation (CIVD) was first described as repeated episodes of warm blood flow to the fingers during cold-water immersion. Since then, hundreds of studies have examined this phenomenon, yet no comprehensive synthesis exists. To address this gap, we conducted a meta-analysis of studies in which the hand, or parts thereof, were immersed for 30 minutes in water below 20°C. A total of 80 studies met the inclusion criteria. Across studies, the weighted onset time of CIVD averaged 7.9 minutes [7.4–8.3], and the mean finger temperature averaged 10.0°C [9.5–10.6]. Onset time was weakly related to finger temperature during immersion (*r* = –0.21 to −0.27), supporting the theory that the onset of CIVD is triggered by low local tissue temperatures, while the magnitude is dependent on sympathetic activity. Onset time was longer for hand versus finger-only immersion, for individuals with a larger surface area, and for males compared to females. Onset time was shorter with higher ambient temperatures, in cold-indigenous populations, and with increasing age. To enrich the meta-analysis, we conducted a narrative literature review of the individual factors and previously proposed mechanisms of CIVD. Current evidence suggests that CIVD is mediated by 1) impaired transfer of noradrenaline from sympathetic nerves to the smooth muscle of the arterio-venous anastomoses or 2) nitric oxide release from these nerves, however, further research is needed to confirm these mechanisms. Future investigations should prioritize including more females and older adults, as these populations remain underrepresented in the literature.

## Introduction

The study of cold-induced vasodilation (CIVD) provides critical insight into how humans can sustain hand function and performance in challenging cold environments. Nearly a century ago, Sir Thomas Lewis first described the cyclic changes in finger skin temperature when the finger is immersed in cold water [1], a phenomenon called CIVD. Since then, several hundred studies have been performed to further elucidate the underlying CIVD mechanisms, differences between populations, the effects of interventions such as residence in cold and/or altitude, and/or the impact of certain pharmacological agents. Although two CIVD literature reviews were published in 2003 [2] and in 2015 [3], a meta-analysis on CIVD responses has not been completed. The lack of a quantitative synthesis limits our ability to determine the relative importance of individual factors. Additionally, since 2015, several new studies have been performed that expand our understanding of CIVD mechanisms and variability. To address these gaps, we conducted a meta-analysis to investigate the influence of environmental, procedural, and personal factors on the CIVD response, as well as the underlying mechanisms. The environmental factors considered were water and air temperature; the procedural factors included finger or hand immersion and presence or absence of a prewarming period of the hand; and personal factors included sex, age, body mass index (BMI), and body surface area (BSA). A disadvantage of a meta-analysis is that many studies must be excluded for not meeting the inclusion criteria; even when they provide valuable insights into underlying mechanisms. A narrative review does not have this disadvantage and enables the construction of a comprehensive text illustrated by a wider range of literature. Therefore, the last paragraph of each section in the narrative review describes the link to the results of the meta-analysis, when applicable. The narrative review includes the personal factors: age, sex, ethnicity, mental stress, pharmacological agents, aerobic exercise and training, body dimensions, and altitude. Having outlined the scope and rationale for combining a meta-analysis with a narrative review, we next describe how CIVD has been measured across studies and clarify the focus of our analysis.

**Figure 1.**
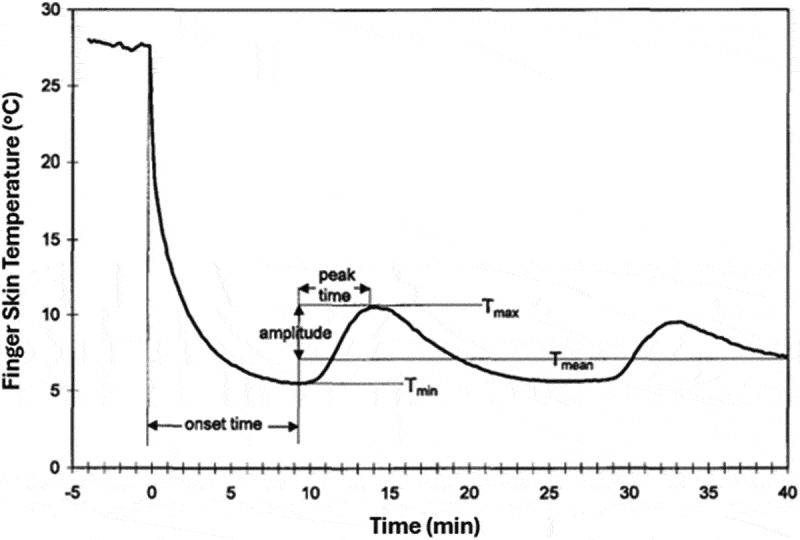
Illustration of fingertip skin temperature during cold-water immersion to display CIVD components. The onset time is the time from immersion to the minimum temperature (T_min_). The amplitude is the difference between T_min_ and the maximum temperature (T_max_). The mean finger skin temperature (T_mean_) denotes the finger skin temperature averaged over the immersion period, excluding the onset time. Figure reprinted with permission from Daanen [2].

The paradoxical vasodilation that occurs when hands/fingers and/or feet/toes are immersed in cold water is generally measured using local skin temperature; however, other methods can be used, such as skin blood flow using laser Doppler flowmetry and more recently laser speckle-based optical flowmetry [4]. In this meta-analysis, only finger skin temperature is included, which reflects the most common measurement technique across CIVD studies. Additionally, CIVD measured in the toes was not included in the analysis due to the limited number of available studies, making meaningful comparisons and robust conclusions difficult. In the review of Daanen [2], a standard protocol was proposed to describe the dependent variables when using finger skin temperature (Figure 1).

The proposed dependent variables by Daanen [2] were included in the meta-analysis: the onset time, minimum finger skin temperature (T_min_), maximum finger skin temperature (T_max_) of the first wave, mean finger skin temperature (T_mean_), and amplitude. The peak time is not included, since it is often not reported.

Beyond its physiological interest, CIVD is relevant to all individuals operating in cold environments. The body’s ability to deliver warm blood to the digits influences not only comfort but also safety, as loss of dexterity and sensation can impair the ability to perform simple tasks, such as buttoning a jacket or safely handling equipment [5]. Furthermore, CIVD has been shown to influence cold-injury risk and may help predict performance and functional capability when exposed to austere cold environments. By presenting the first comprehensive meta-analysis followed by a narrative review, this work consolidates decades of research and establishes a foundation for understanding how individual, environmental, and procedural factors influence CIVD responses.

## Methods for meta-analysis

### Eligibility criteria

Records were selected for inclusion if they met the following criteria: finger only or whole-hand immersions of 30 minutes or more, water temperatures lower than 20°C, finger skin temperature was reported, and papers were included up to the year 2024. For intervention studies, only the control values were extracted (e.g. prior to cold or altitude exposure or prior to the use of medication). The exclusion criteria were non-human investigations (i.e. animals), infant/pediatric populations, participants with a pathological condition (e.g. Raynaud’s phenomenon), and studies without extractable finger skin temperature data.

### Information sources

A comprehensive meta-analysis was conducted by first conducting an algorithmic database search using Scopus, which is the largest abstract and citation database of peer-reviewed literature, including scientific journals, books, and conference proceedings (https://blog.scopus.com/about). An additional 45 articles from a personal database were added to the Scopus database, and duplicate analysis showed that 15 of those 45 were not found in the initial Scopus results for diverse reasons, such as it originated from an unindexed journal, or it contained unusual keywords. Searches were completed in April 2023 and updated in May 2025.

### Search strategy

Search terms and keywords employed were “cold induced vasodilation” OR “cold-induced vasodilation” OR “cold induced vasodilatation.” Search algorithms were systematically applied, and duplicate records were removed by PMID and/or DOI prior to screening.

### Selection process

**Figure 2.**
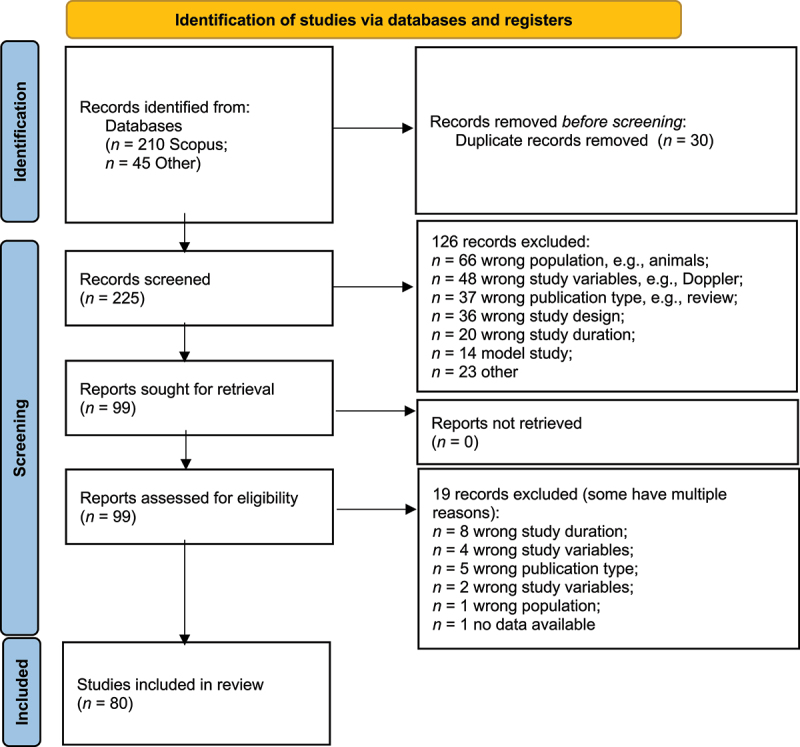
PRISMA flow chart.

Two reviewers independently screened titles, abstracts, and full texts to determine eligibility based on the inclusion criteria using the open-source web application Rayyan QCRI [6]. If disagreements arose, a third reviewer evaluated the full-text record and served as the tiebreaker. Of the 225 peer-reviewed papers screened for study selection (including 15 from the personal database, i.e. 7%), 80 studies were selected for inclusion in the meta-analysis. The process is summarized in the PRISMA flow diagram (Figure 2).

### Data collection process

Data extraction was performed independently by three reviewers. Numerical data was taken directly from tables when available. Both mean values and standard deviations (*SD*) from the reports served as the input for the dataset. If 95% confidence intervals (CI) were reported, the values were converted to *SD* (95% is 1.96 *SD*). When standard error (*SE)* was reported, values were recalculated to *SD*. When the results of separate fingers were reported, the values were averaged, and *SD* was recalculated. The tool WebPlotDigitizer [7] was used to extract the data from figures if there were no tables with information supplied.

### Data items

#### Coded dependent variables extracted included

1. Onset time – the time from immersion to the lowest temperature prior to the first vasodilation in minutes.
2. T_min_ – minimum temperature in °C prior to the first vasodilation. Please note that it may not be the lowest temperature during immersion.
3. T_max_ – maximum temperature in °C of the first vasodilation response.
4. T_mean_ – average temperature in °C of the immersion period minus the onset time. Please note that other authors have defined as 1) averaged over total immersion time or 2) averaged over total immersion time minus the initial 2, 5, or 10 minutes, thus highlighting that standardization of this parameter is poor.
5. Amplitude – reported amplitude in °C, if not provided then replaced by T_max_ minus the T_min_.

Continuous moderator variables (bold) included both individual and procedural moderators.

#### Individual moderators/personal factors

1. **PropMF** – proportion male in the dataset
2. **Age** – mean age of the participants in years
3. **Age*****SD*** – standard deviation of age in years
4. **Stature** – mean stature of the participants in centimeters
5. **Stature*****SD*** – standard deviation of the stature in centimeters
6. **Weight** – mean body weight of the participants in kilograms
7. **Weight*****SD*** – standard deviation of body weight in kilograms
8. **Country –** country of the participants, using ISO coding Alpha 3 (https://www.nationsonline.org/oneworld/country_code_list.htm). Please note that when participants were from several countries, the dominant country served as input.
9. **Ethnicity** – Neg for Negroid, Cau for Caucasian, Mon for Mongoloid, and Oth for unknown

#### Procedural moderators

1. **WaterT** – Water temperature in °C
2. **Duration** – Duration of immersion in minutes
3. **Fingermeas** – measured finger, 1 for thumb, 2 for index, 3 for middle finger, 4 for ring finger, 5 for pinky, 6 for hand
4. **Fingerimm** – immersed finger, 1 for thumb, 2 for index, 3 for middle finger, 4 for ring finger, 5 for pinky, 6 for hand
5. **Part_immersed_** −1 for finger, 2 for hand
6. **PrewarmT** – temperature of water used during prewarming in °C
7. **PrewarmD** – duration of prewarming in minutes
8. **Prewarm** – if prewarming was present (1) or not (0)
9. **T_amb_** – Ambient temperature in °C
10. **RH** – relative humidity in %
11. **ColdInd** – −1 when from cold region, 0 neutral, 1 when from warm region

#### Coding other parameters

1. First author – first author of publication
2. Year – year of publication
3. *N* – number of subjects in the study
4. Female – number of females in the study
5. Male – number of males in the study

#### Calculated dependent variables

The following dependent variables were calculated, but these derived parameters were not included in the meta-analysis:

1. BMI – Body mass index (body weight in kg divided by stature in m squared)
2. BSA – Body surface area [8]
3. SM – Surface to mass ratio

### Effect measures

The primary effect measures were mean values and standard deviations for each CIVD variable. From these, we calculated meta-analytic means, standard errors, 95% CIs, and prediction intervals.

### Synthesis methods

R [9] was used to analyze the data, and the RStudio version was 2023.12.1. To process the data, we applied the R packages *dmetar* [10], *dplyr* [11], *future* [12], *parallel* [9], *parallelly* [13], *readxl* [14], *RoBMA* [15], *tidyverse* [16], *utils* [9], and *writexl* [17]. To summarize the data, we used *psych* [18], and to visualize the data, we applied *ggplot2* [19] and *orchard* [20]. To conduct the meta-analysis, we used *clubSandwich* [21], *metafor* [22], and *metaplus* [23].

For the meta-analysis, we primarily used the *metafor* R package to fit a random-effects model. The studies were weighed according to their respective sample sizes and standard deviations of their group mean values. The main results were reported as meta-analytic means and their standard errors, 95% CIs for the means, and prediction intervals. The latter reflected the mean distribution for potential replication studies. We used Bayesian statistics to compute robust estimation of the means and heterogeneity of the means (Supplemental Figures 21–30).

We used non-aggregated data and, as multiple effect sizes (i.e. means) were extracted from the same study, we applied a multilevel model, where effect sizes were nested within studies. The application of this model resulted in the disentanglement of two types of variances: within and between studies. As a result, we could calculate the within, between, and total level of heterogeneity reported as *I*^2^ [24].

In the moderation analyses, we used a linear mixed-effects model. The results were visualized in forest and funnel plots. The sensitivity analyses were conducted by means of the *metaplus* R package [23] that utilizes an iterative process to detect outliers and correct for, if any, is detected. We conducted robust *t*-distribution analyses and compared them with mixture random-effects model analyses, and to identify potential outliers, we compared the outputs of both analyses. If the LRT statistic was statistically significant, we concluded that outliers were present, and if the *D*-statistic was statistically non-significant, the distributions of effect sizes with and without outliers had a similar fit, excluding the outlier was justified without impeding the quality of meta-analytic estimation and prediction.

The CIVD response (onset time, T_min_, T_max_, T_mean_, and amplitude) was related to personal factors (sex, age, body dimensions, ethnicity), ambient factors (water and air temperature), and protocol (prewarming presence, immersion of fingers or hand). The following procedure was followed to report the results:

1. An overview was provided of the number of articles and populations that provided means and *SD* for the dependent variables.
2. Correlations between the dependent variables were calculated to indicate the interrelations between the dependent variables.
3. A multivariate meta-analysis model (restricted maximum likelihood; REML) with a test for heterogeneity was performed for each dependent variable; confidence and prediction intervals were calculated.
4. Forest plots were provided for each dependent variable.
5. A moderator analysis was performed for each dependent variable with consecutive moderators: WaterT, Part_immersed_, T_amb_, Prewarm, ColdInd, PropMF, Age, BMI, and BSA. These moderators were ranked for their estimated relevance.
6. A combined model of moderators was evaluated. WaterT, Part_immersed_, T_amb_, Prewarm, Cold_Ind_ were used in stepwise combinations.
7. Sensitivity analyses were performed: Contour-enhanced funnel plots and outlier analyses were presented.
8. A robust Bayesian meta-analysis was performed, resulting in robust estimation of means (Supplemental Figures 21–30).

## Results of meta-analysis

### Retrieved articles

The 80 included papers are presented in Table 1.

**Table 1.** All studies evaluating CIVD included in the meta-analysis (*N* = 80). “Fingers measured” represents the digits that finger skin temperature was measured to determine CIVD components.

Study	Males	Females	Water temp (°C)	Fingers measured
[25]	10	1	4	Middle
[26]	9	0	8	All
[27]	9	0	0	Index
[28]	10	0	8	All
[29]	40	0	8	All
[30]	8	0	5	All
[31]	16	0	8	All
[32]	9	0	8	Middle
[33]	148	0	0	Middle
[34]	13	0	0	Middle
[35]	14	2	10	All
[36]	8	4	9	Index
[37]	10	7	8	Index
[38]	4	4	8	Index
[39]	7	4	8	Index
[40]	5	0	8	All
[41]	5	0	8	All
[42]	9	0	8	Index
[43]	6	0	2	Index, Pinky
[44]	11	0	5	Middle
[45]	7	0	8	All
[46])	8	0	8	All
[47]	14	0	8	All
[48]	24	0	8	All
[49]	18	0	8	All
[50]	0	17	4	Middle
[51]	7	5	0	Index, Middle, Ring
[52]	0	30	4	Middle
[53]	8	0	0	Middle
[54]	0	13	0	Middle
[55]	24	0	4	Middle
[56]	0	213	4	Middle
[57]	10	7	5	Middle, Ring
[58]	129	0	0	Middle
[59]	3	0	0	Middle
[60]	10	0	8	Index, Middle
[61]	15	0	4	Index
[62]	36	0	4	Index
[63]	40	0	4	Index
[64]	17	0	4	Index
[65]	135	0	4	Index
[66]	9	0	15, 10, 8, 5	All
[67]	9	3	5	Index, Middle, Ring, Pinky
[68]	58	12	8	All
[69]	19	2	4	Middle
[70]	9	0	4	Middle
[71]	2	0	4	Middle
[72]	12	2	4	Middle
[73]	19	3	0	Middle
[74]	9	34	4	Middle
[75]	12	0	4	Index
[76]	7	0	4	Index
[77]	32	0	4	Index
[78]	40	0	4	Index
[79]	10	0	5	Index, Ring
[80]	10	10	0, 5, 10	Middle
[81]	20	6	0.3, 0.7	Middle
[82]	20	0	5	Index
[83]	12	0	5	Index
[84]	16	2	5	Middle
[85]	29	0	5	Middle
[86]	60	0	0	Middle
[87]	38	0	0	Middle
[88]	50	0	0	Middle
[89]	214	0	0	Middle
[90]	24	0	0	Middle
[91]	0	24	0	Middle
[92]	15 total		0	Middle
[93]	37 total		0	Middle
[94]	6	1	8	Index
[95]	7	7	8	Index
[96]	5	0	0	Index
[97]	15	15	0, 8	Index
[98]	11	0	5	Index, Middle, Ring
[99]	20	0	8	Index
[100]	7	0	5	Middle
[101]	8	2	4	Index, Middle, Ring
[102]	10	5	8	Index, Pinky
[103]	10	0	8	Index, Pinky
[104]	84	0	5	Middle

### General description of the included datasets

The database of 80 studies contained 143 coded populations. In 10 studies, comparisons were made within subjects. The 10 studies compared several variables within subjects, including: differences between nailbed and finger pad temperature [25,69], left versus right hand [39], comparison of different water temperatures [66,80,97], comparison of room temperatures [84], comparison of euhydration versus hypohydration [72], and comparison of hand versus finger immersion [82,83] were evaluated. One study measured the population 4 times [66], and another study measured it 3 times [80]; therefore, 130 different populations (datasets) remained in the analysis. Table 2 provides an overview of the dataset’s composition used for analysis.

**Table 2.** The number of articles (a) or study populations (p) for single (s) and multiple (M) effect sizes for which mean and standard deviation were available. “#p within” means the number of studies in which the study population was used for more than one measurement. “#p no doubles” are the number of studies in which the population was only measured once.

Variable	#A	#A S	#A M	#P	#P within	#P no doubles
Onset	59	25	34	108	7	101
T_min_	70	33	37	125	11	114
T_max_	51	26	25	87	10	77
T_mean_	58	30	28	101	8	93
Amplitude	36	13	23	69	7	62

In total, 358 females and 1835 males were included. Thus, 84% of study participants were male and only 16% were female. The number of males and females was not specified in two studies: one with 37 participants [93] and another with 15 participants [92].

Table 3 shows the countries from which the participants in the datasets were recruited. One study [55] constructed a participant pool from a selection of countries around the equator (“Several” in the table). Only one study had a group of 10 participants of African descent [60].

**Table 3.** The countries from which the participants were recruited in the 130 datasets. Country code is according to ISO 3166.

Country	Datasets
JPN	28
IND	18
CAN	16
SVN	14
USA	12
KOR	10
NLD	7
GBR	6
RUS	4
SWE	4
FRA	3
GRC	3
CHN	2
Several	1
NOR	1
DEU	1

In 55% of the datasets, the hands were immersed to the wrist level; in 40% of the datasets, the middle fingers were only immersed, and in 3%, the index finger alone was immersed. In one study, the index and little fingers of both hands were immersed to the level of the proximal interphalangeal joints [43], and in another study, all fingers but the thumb to the second interphalangeal joints were immersed [67]. The measured fingers for all datasets were thumb (15%), index (52%), middle (68%), ring (21%), and little finger (20%).

The immersion water temperature was approximately 4°C in 42% of the datasets, 32% in ice water, and 26% at approximately 8°C. Prewarming was performed in 26% of the datasets. The prewarming water temperature averaged 35.5 ± 3.6°C (range: 20–42°C).

The age of the subjects was not reported in 4 studies or 11 datasets [69,85,93,105]. The age was under 20 y in 16%, from 20–30 y in 60%, from 30–40 y in 15% and over 40 y in 9% of the datasets. BMI and BSA could be calculated in 83 datasets (64%) and were 23.2 ± 2.0 kg/m^2^ and 1.8 ± 0.2 m^2^, respectively.

### Interrelation between dependent variables

All dependent variables were available in 58 datasets out of 143. Table 4 shows the correlations. T_min_, T_max_, and T_mean_ were highly correlated.

**Table 4.** Meta-regression estimates for the relationships between the dependent variables: amplitude, T_min_, T_max_, T_mean_ in °C, and onset time in minutes. Bold values were statistically significant (*p* < 0.05).

	Onset time	T_min_	T_max_	T_mean_	Amplitude
Onset time (min)	–	**−0.27**	**−0.21**	**−0.23**	**−0.16**
T_min_ (°C)		–	**0.65**	**0.81**	**−0.29**
T_max_ (°C)			–	**0.98**	**0.63**
T_mean_ (°C)				–	0.17

**Figure 3.**
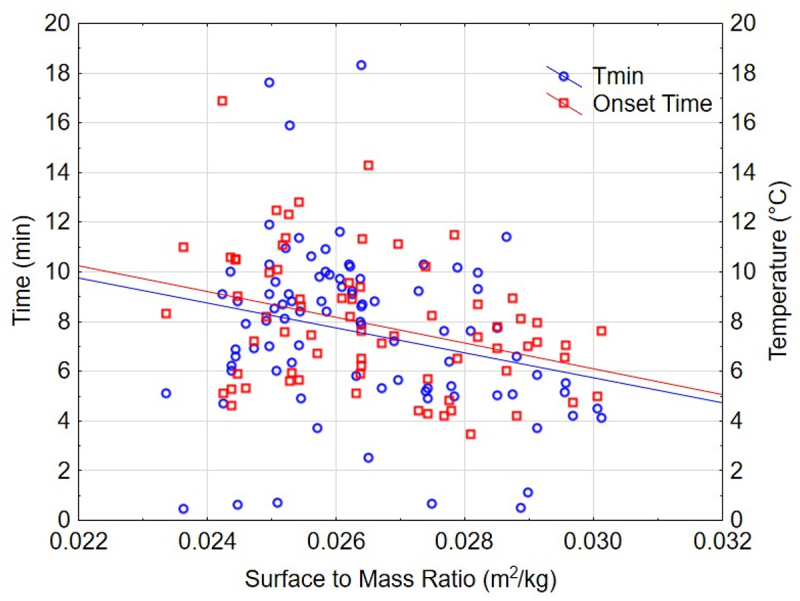
The onset time (red) and T_min_ (blue) are plotted against the surface-to-mass ratio, with regression lines shown.

The surface-to-mass ratio was related to the onset time and T_min_ (Figure 3). Subjects with a relatively larger skin surface area compared to body mass showed lower T_min_ values and shorter onset times.

### Meta-analysis for the dependent variables

Table 5 summarizes the results of the meta-analysis for the dependent variables.

**Table 5.** Meta-analytical means with standard errors for the dependent variables: amplitude, T_min_, T_max_, T_mean_ in °C, and the onset time in minutes. M_+_ = meta-analytical mean. *SE*= standard error of mean. Bold values represent the *z*-value, and others represent the *t*-value. All means were significant at *p* < 0.001; CI = confidence interval; PI = prediction interval; Lb = lower bound, UB = upper bound; *df* is degrees of freedom for *Q* = heterogeneity that was significant for all variables at *p* = 0.001. σ^2^b stands for between-studies variance, and σ^2^w shows the variance within studies. Similarly, *I*^2^b stands for the heterogeneity variance between studies, and *I*^2^w for within studies; *I*^2^tot is the total heterogeneity variance.

	*M*_+_	*SE*	*z*/*t*	CI LB	CI UB	PI LB	PI UB	*df*	*Q*	σ^2^b	σ^2^w	*I*^2^tot	*I*^2^b	*I*^2^w
Onset time (min)	7.9	0.2	33.00	7.4	8.3	3.4	12.4	107	4349.9	3.39	1.68	98.5%	69.9%	28.6%
T_min_ (°C)	7.2	0.3	**24.50**	6.6	7.7	1.00	13.3	124	35725.3	3.07	6.72	99.7%	32.8%	66.9%
T_max_ (°C)	12.5	0.4	34.27	11.8	13.2	5.9	19.0	86	5075.4	0.24	10.49	98.5%	0.0%	97.9%
T_mean_ (°C)	10.0	0.3	**34.97**	9.5	10.6	4.6	15.5	100	11921.2	1.25	6.42	99.3%	19.4%	79.7%
Amplitude (°C)	6.1	0.5	**13.30**	5.2	7.0	0.00	13.21	68	3841.1	8.02	4.84	99.3%	15.6%	83.7%

### Forest plots

**Figure 4.**
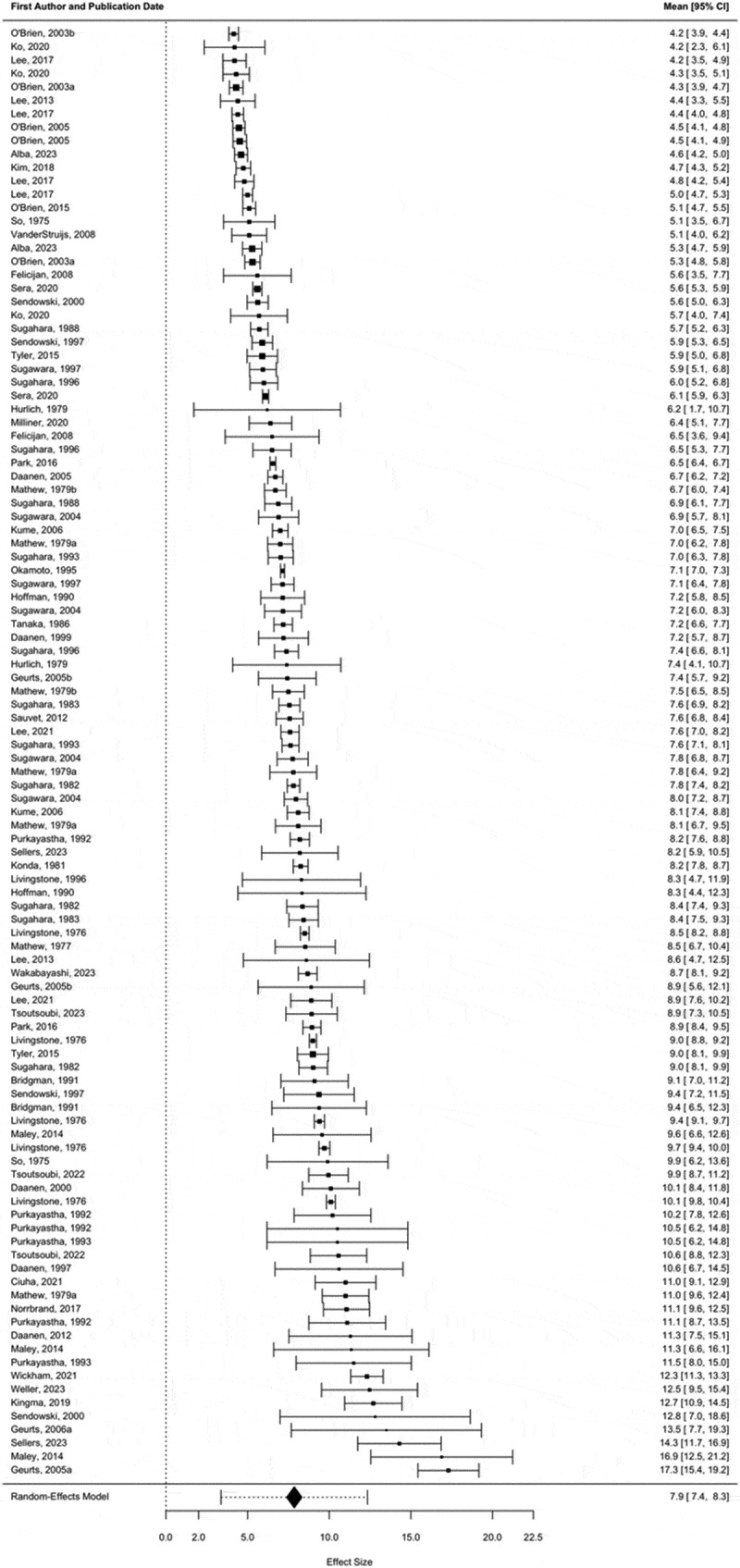
Forest plot of onset time representing the meta-analytical means. The black diamond represents the confidence intervals, and the dashed lines on both sides of the diamond represent the prediction intervals.

Forest plots enable the visual representation of study heterogeneity and display the estimated common effect in a single figure. The forest plot for onset time is shown in Figure 4. The forest plots for T_min_, T_max_, T_mean_, and amplitude are presented in Supplemental Figures 1–4.

### Moderator analysis

The results of the moderator analysis are presented in Table 6.

**Table 6.** Estimated moderator effects with standard errors (*SE*) for the dependent variables: amplitude, T_min_, T_max_, T_mean_ in °C, and onset time in minutes for the moderators: water temperature, Part_immersed_ (hand or finger), ambient temperature (T_amb_), Prewarm, cold indigenous, proportion male over female, age, BMI, and BSA. CI = confidence interval; Lb = lower bound; UB = upper bound; *I*^2^ tot = total heterogeneity after controlling for the moderator in each separate model. For cold indigenous populations, the difference between warm and cold indigenous people was tested. Bold values are significant.

Variable	Moderator	*Effect*	*SE*	*z*	*p*	CI LB	CI UB	*I*^2^ tot
Onset time (min)	Water temperature	0.09	0.08	1.09	0.274	−0.07	0.24	98.43%
	**Part immersed**	**2.18**	**0.44**	**5.02**	**<.001**	**1.33**	**3.04**	**98.01%**
	**Tamb**	**−0.30**	**0.08**	**−3.70**	**<.001**	**−0.45**	**−0.14**	**98.21%**
	Prewarm	0.63	0.62	1.02	0.309	−0.58	1.84	98.51%
	**Cold indigenous**	**−3.16**	**1.17**	**−2.70**	**0.007**	**−5.46**	**−0.87**	**98.37%**
	**propMF**	**1.61**	**0.59**	**2.74**	**0.006**	**0.46**	**2.76**	**98.36%**
	**age**	**−0.04**	**0.02**	**−2.20**	**0.028**	**−0.08**	**−0.01**	**98.21%**
	BMI	0.20	0.14	1.47	0.142	−0.07	0.46	97.15%
	**BSA**	**4.80**	**1.50**	**3.20**	**0.001**	**1.86**	**7.73**	**96.81%**
T_min_ (°C)	**Water temperature**	**0.72**	**0.06**	**11.40**	**<.001**	**0.59**	**0.84**	**99.32%**
	**Part immersed**	**2.42**	**0.54**	**4.52**	**<.001**	**1.37**	**3.47**	**99.62%**
	Tamb	0.10	0.11	0.93	0.354	−0.11	0.31	99.69%
	**Prewarm**	**2.83**	**0.61**	**4.67**	**<.001**	**1.64**	**4.02**	**99.63%**
	Cold indigenous	0.41	1.35	0.30	0.761	−2.24	3.06	99.67%
	**propMF**	**2.35**	**0.78**	**3.04**	**0.002**	**0.83**	**3.87**	**99.65%**
	age	−0.01	0.03	−0.22	0.824	−0.06	0.04	99.67%
	BMI	0.27	0.18	1.53	0.125	−0.08	0.62	99.66%
	**BSA**	**7.01**	**1.83**	**3.83**	**<.001**	**3.42**	**10.60**	**99.61%**
T_max_ (°C)	**Water temperature**	**0.45**	**0.13**	**3.51**	**<.001**	**0.20**	**0.70**	**98.24%**
	Part immersed	−0.44	0.76	−0.58	0.561	−1.93	1.05	98.48%
	Tamb	−0.08	0.13	−0.61	0.544	−0.32	0.17	98.50%
	**Prewarm**	**1.50**	**0.74**	**2.03**	**0.042**	**0.05**	**2.94**	**98.40%**
	Cold indigenous	2.35	1.53	1.54	0.124	−0.64	5.34	98.25%
	propMF	1.88	1.10	1.72	0.086	−0.27	4.03	98.38%
	age	−0.04	0.03	−1.30	0.193	−0.09	0.02	98.43%
	BMI	0.21	.23	0.91	0.365	−0.24	0.65	98.08%
	**BSA**	**6.29**	**2.33**	**2.70**	**0.007**	**1.73**	**10.86**	**97.85%**
T_mean_ (°C)	**Water temperature**	**0.31**	**0.08**	**3.79**	**<.001**	**0.15**	**0.47**	**99.15%**
	Part immersed	0.78	0.57	1.36	0.173	−0.34	1.89	99.21%
	Tamb	0.09	0.12	0.76	0.447	−0.14	0.32	99.27%
	Prewarm	0.71	0.65	1.10	0.270	−0.55	1.98	99.26%
	Cold indigenous	1.03	1.39	0.74	0.459	−1.69	3.74	99.20%
	propMF	0.71	0.77	0.92	0.358	−0.80	2.22	99.23%
	age	−0.03	0.02	−1.18	0.238	−0.07	0.02	99.20%
	BMI	−0.06	0.16	−0.34	0.732	−0.38	0.27	99.23%
	BSA	2.29	1.85	1.24	0.216	−1.34	5.93	99.20%
Amplitude (°C)	**Water temperature**	**−0.76**	**0.09**	**−8.29**	**<.001**	**−0.93**	**−0.58**	**98.17%**
	**Part immersed**	**−4.79**	**0.68**	**−7.05**	**<.001**	**−6.12**	**−3.46**	**98.47%**
	Tamb	0.07	0.20	0.35	0.730	−0.33	0.47	99.15%
	**Prewarm**	**−3.43**	**1.04**	**−3.31**	**<.001**	**−5.46**	**−1.40**	**98.96%**
	Cold indigenous	−0.44	2.88	−0.15	0.878	−6.09	5.21	99.14%
	propMF	−1.69	1.03	−1.65	0.100	−3.71	0.32	99.11%
	**age**	**−0.07**	**0.03**	**−2.23**	**0.026**	**−0.12**	**−0.01**	**99.09%**
	**BMI**	**−0.84**	**0.24**	**−3.51**	**<.001**	**−1.31**	**−0.37**	**99.03%**
	**BSA**	**−8.02**	**2.73**	**−2.94**	**0.003**	**−13.37**	**−2.67**	**99.06%**

The onset time was significantly longer for hand versus finger immersion, for participants with a relatively large body surface area, and in males compared to females. Onset time was significantly shorter when ambient temperature increases, in cold-indigenous people, and with increasing age. Furthermore, onset time was unrelated to water temperature, prewarming of the hand, and BMI.

T_min_ was significantly higher during hand immersion compared to finger immersion, with prewarming, in males compared to females, and in individuals with greater body surface area. T_min_ also increased significantly with water temperature. In contrast, ambient temperature, cold-indigenous status, age, and BMI were not related to T_min._

T_max_ was significantly higher with prewarming, in individuals with greater BSA, and with increasing water temperature. No significant effects were observed for part immersed, ambient temperature, cold-indigenous status, sex, age, or BMI.

T_mean_ increased significantly with water temperature, while all other moderators were not related to T_mean._

Amplitude was significantly lower for hand immersion compared to finger-only immersion, with prewarming, in participants with higher BMI, greater BSA, and with increasing age. Amplitude also decreased significantly with higher water temperature. In contrast, ambient temperature, cold-indigenous status, and sex were not related to amplitude.

### Combined model of moderators

Table 7 shows the results of the combined model.

**Table 7.** Estimated moderator effects with standard error (*SE*) for the dependent variables: amplitude, T_min_, T_max_, T_mean_ in °C, and onset time in minutes for the consecutive moderators: water temperature, Part_immersed_ (hand or finger), ambient temperature (T_amb_), prewarm, proportion male over female, and cold indigenous combined. CI = confidence interval; Lb = lower bound; UB = upper bound. Bold values are significant.

Variable	Moderator	Effect	*SE*	*z*	*p*	CI LB	CI UB	*I*^2^ tot
Onset time (min)	Intercept	14.20	1.93	7.37	<.001	10.43	17.98	97.27%
	**WaterT**	**−0.27**	**0.09**	**−3.16**	**0.002**	**−0.43**	**−0.10**	
	**Part immersed**	**3.00**	**0.52**	**5.76**	**<.001**	**1.98**	**4.02**	
	Prewarm	0.46	0.62	0.75	0.451	−0.74	1.67	
	**Tamb**	**−0.27**	**0.07**	**−3.74**	**<.001**	**−0.41**	**−0.13**	
	PropMF	−0.08	0.59	−0.14	0.888	−1.24	1.07	
	ColdInd	0.71	0.43	1.66	0.096	−0.13	1.54	
T_min_ (°C)	Intercept	0.65	1.90	0.34	0.734	−3.08	4.38	99.06%
	**WaterT**	**0.97**	**0.09**	**10.44**	**<.001**	**0.79**	**1.15**	
	**Part immersed**	**−2.07**	**0.55**	**−3.75**	**<.001**	**−3.15**	**−0.99**	
	Prewarm	−0.60	0.54	−1.12	0.264	−1.65	0.45	
	Tamb	0.06	0.07	0.83	0.405	−0.08	0.20	
	**PropMF**	**2.53**	**0.61**	**4.14**	**<.001**	**1.33**	**3.72**	
	ColdInd	−0.65	0.40	−1.62	0.105	−1.44	0.14	
T_max_ (°C)	Intercept	9.11	3.02	3.02	0.003	3.19	15.03	97.58%
	**WaterT**	**0.82**	**0.17**	**4.92**	**<.001**	**0.50**	**1.15**	
	**Part immersed**	**−3.62**	**0.87**	**−4.14**	**<.001**	**−5.33**	**−1.91**	
	Prewarm	−0.48	0.78	−0.62	0.537	−2.01	1.05	
	Tamb	−0.07	0.11	−0.65	0.514	−0.28	0.14	
	**PropMF**	**3.76**	**1.15**	**3.27**	**0.001**	**1.51**	**6.01**	
	ColdInd	−1.22	0.69	−1.76	0.078	−2.58	0.14	
T_mean_ (°C)	Intercept	7.79	3.18	2.45	0.014	1.57	14.02	98.90%
	WaterT	0.57	0.14	4.18	<.001	0.30	0.84	
	**Part immersed**	**−1.77**	**0.83**	**−2.15**	**0.032**	**−3.39**	**−0.15**	
	Prewarm	−1.09	0.76	−1.44	0.150	−2.57	0.39	
	Tamb	0.01	0.12	0.03	0.976	−0.24	0.24	
	PropMF	1.23	0.81	1.53	0.127	−0.35	2.81	
	ColdInd	−0.08	0.586	−0.14	0.887	−1.23	1.07	
Amplitude (°C)	**Intercept**	**16.53**	**4.08**	**4.05**	**<.001**	**8.53**	**24.54**	97.47%
	**WaterT**	**−0.42**	**0.11**	**−3.75**	**<.001**	**−0.63**	**−0.20**	
	**Part immersed**	**−2.99**	**0.79**	**−3.77**	**<.001**	**−4.55**	**−1.44**	
	Prewarm	−0.65	0.92	−0.70	0.484	−2.45	1.16	
	Tamb	−0.28	0.15	−1.82	0.069	−0.58	0.02	
	PropMF	−0.60	0.82	−0.73	0.468	−2.21	1.01	
	**ColdInd**	**2.41**	**0.80**	**2.99**	**0.003**	**0.83**	**3.98**	

### Sensitivity analysis

**Figure 5.**
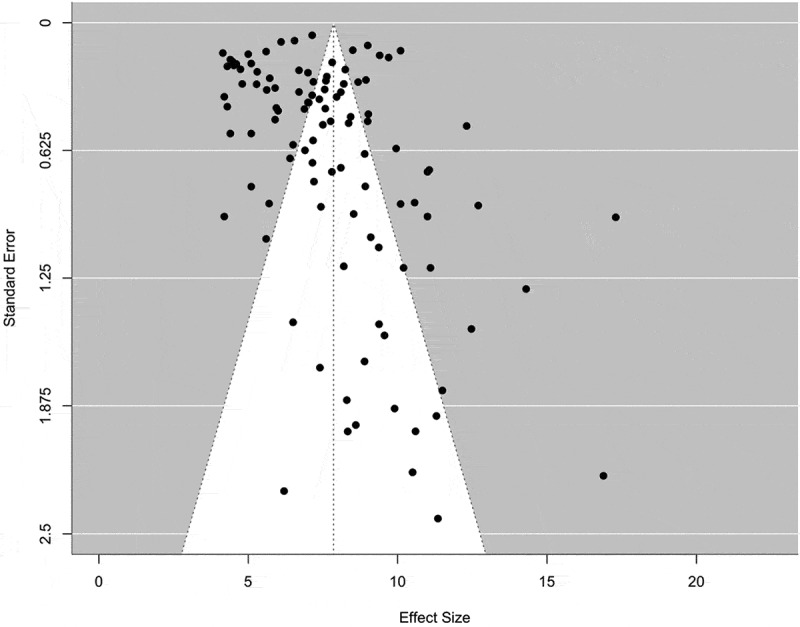
Funnel plot for onset time representing the meta-analytical means.

The funnel plot for onset time is shown in Figure 5; the contour-enhanced and standard funnel plots for the onset time and all other variables are available in Supplemental Figures 5–8.

**Figure 6.**
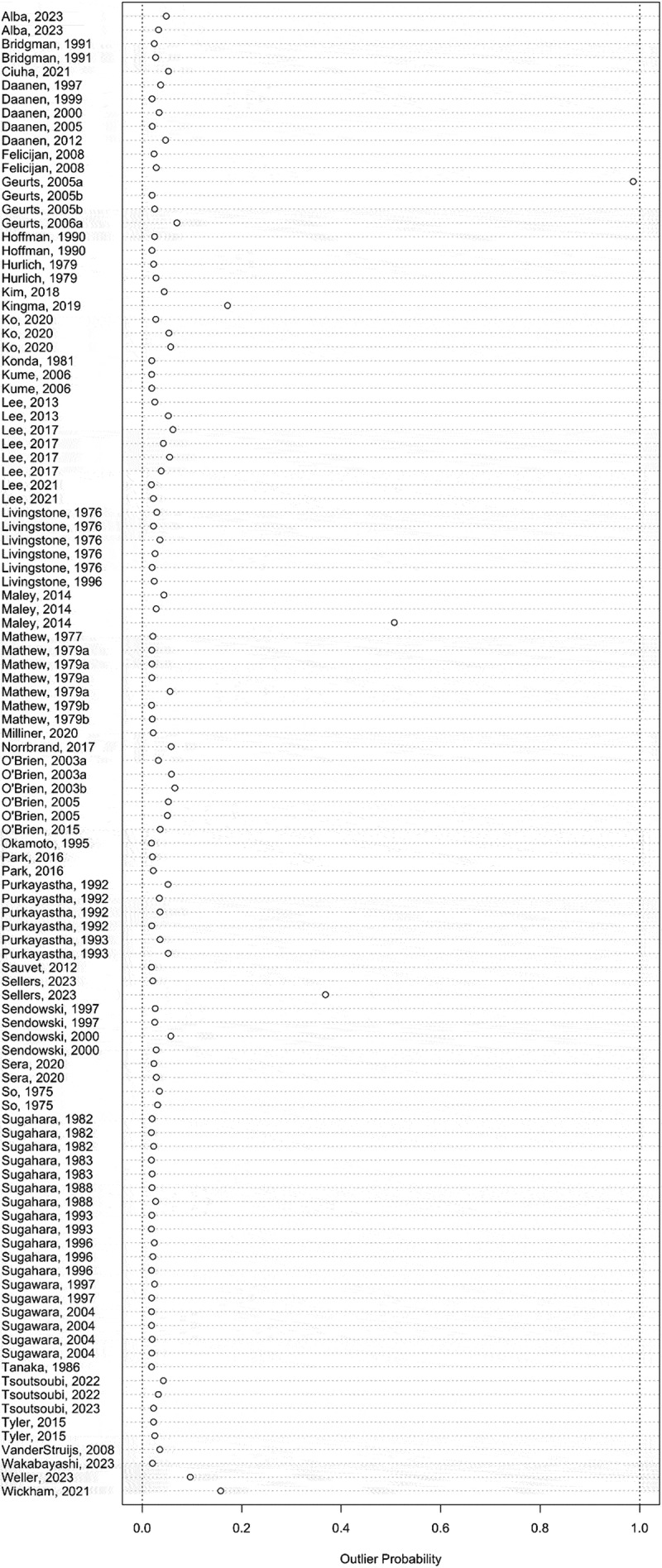
The outlier probabilities for onset time from the robust mixture random effects model.

**Figure 7.**
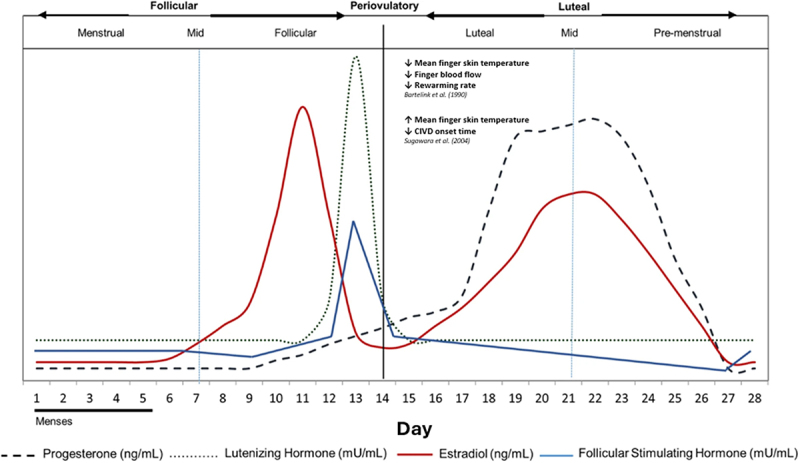
Typical 28-d menstrual cycle displaying the changing concentrations of female sex hormones through the menstrual phases, highlighting that female sex hormones may influence CIVD responses. Changes in peripheral blood flow and finger skin temperatures are described. Figure adapted with permission [106].

**Figure 8.**
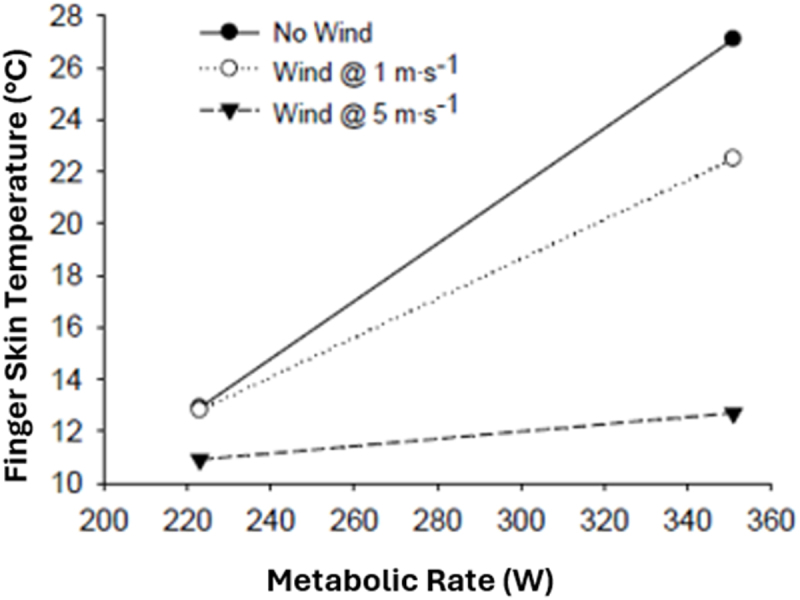
Finger temperatures during treadmill walking at 220 and 350 watts in −10°C air in three different wind conditions. Figure reprinted with permission [107].

In this funnel plot, the mean values are plotted on the horizontal axis, and the standard error is plotted on the vertical axis. A small standard error (generally due to a large sample size) should be related to a narrower band around the mean effect size (7.86 minutes). However, in the meta-analysis, several moderators have been shown to potentially explain the differences in the onset time between the studies, such as ambient temperature and immersion of finger and/or hand in the cold water. This resulted in a large scatter, even when the standard error was very small. Notably, the two articles with effect sizes larger than 15 minutes [36,60] can be explained by the use of electrical stimulation during immersion and including people of African descent, respectively.

### Outlier analyses

Outliers were identified using the *metaplus* R package. Figure 6 shows the outliers for onset time, LRT = 2.50, *p* = 0.030, *D*(1) = 2.45, *p* = 0.117, in which the previously mentioned articles [37,60] dominate again; the probabilities of outliers and the corrected effect sizes (based on robust *t*-distribution), and plots for the other dependent variables can be found in Supplemental Figures 14–17 and 23–27, respectively.

## Meta-analysis general conclusions

The meta-analysis demonstrated a significant yet low correlation between onset time and finger skin temperature during immersion, suggesting that while low local tissue temperature influences CIVD onset time, its contribution is modest, and the response magnitude remains primarily governed by sympathetic activity. Personal factors (age, sex, body dimensions, and ethnicity), ambient factors (water and ambient temperature), and immersion protocol have an impact on CIVD timing and magnitude.

T_min_ was lower when only the finger was immersed, when prewarming was not conducted, and among females. Water temperature significantly influenced T_min_, T_max_, T_mean_, and amplitude. As expected, higher water temperatures yielded higher T_min_, T_max_, and T_mean_ values, though amplitude decreased with increasing water temperature. T_min_ was lower for finger-only immersions compared to whole-hand immersions, though T_max_ and T_mean_ were unaffected, resulting in a larger amplitude for finger-only conditions. Although immersion of the hand has been associated with increased sympathetic activity and lower finger temperatures [83], our data showed lower T_min_ with finger-only immersion. Ambient temperature had no observable effect on finger temperatures, although the average across studies was relatively narrow (24.6 ± 2.9°C). Prewarming increased T_min_ and reduced amplitude, likely due to reduced cooling rates from residual hand heat. No significant differences in temperature response were observed between warm- and cold-indigenous populations. T_min_ was lower among females, which may relate to their lower initial finger temperatures. Age and BMI had no significant impact on T_mean_, T_min_, or T_max_, though amplitude decreased with increasing BMI. Higher surface area-to-mass ratios were associated with lower T_min_ values (Figure 3).

When a combined moderator model was applied (Table 7), using the sequence of water temperature, part immersed, prewarming, ambient temperature, sex proportion, and cold-indigenous status, the findings were consistent with those of individual meta-analyses (Table 6). For onset time, the most explanatory moderators were part immersed and ambient temperature; however, in the combined model, water temperature also emerged as a significant predictor. The mean onset time was 8 minutes when water temperature was about 1°C and 7 minutes when water temperature was about 6°C. The modeling approach allows for practical estimation of the onset time in future studies where moderator characteristics are known. For instance, in a study with 8°C water temperature, 25°C ambient temperature, finger immersion with prewarming, and cold-indigenous male participants, an onset time of 8 minutes can be expected, assuming 95% precision. Increasing the ambient temperature to 30°C would reduce the expected onset time to 6.7 minutes, assuming 95% precision.

A key limitation of this meta-analysis lies in the composition of the dataset, which, although comprehensive and inclusive of articles published in other languages (e.g. Japanese), reflects a demographic bias. Young males are overrepresented across the studies, and only a single study included participants of African descent. This lack of diversity restricts the generalizability of the findings to broader populations.

Additionally, inconsistencies in data reporting among the included studies present another limitation. While 70 out of 80 studies reported T_min_ and its standard deviation, only 59 reported the onset time, and 58 included T_mean_. However, the time span over which T_mean_ was calculated varied substantially: 34% of studies used the full 30-minute immersion period, 31% used the 5–30-minute window, 12% used 6–30 minutes, 10% calculated the mean from onset time to the end of immersion, and the remaining 13% used other time frames. T_max_ was reported in 51 studies, and amplitude (with standard deviation) in only 36 studies. These inconsistencies hinder direct comparisons across studies. For future research, it is recommended that all five dependent variables: T_min_, onset time, T_mean_, T_max_, and amplitude, be consistently reported alongside their standard deviations and calculations. Furthermore, although T_mean_ is frequently calculated over the full immersion period, excluding the initial 5 minutes may yield a more accurate representation of the CIVD response, as this early phase primarily reflects the dissipation of residual heat from the hand rather than true vasodilatory activity.

## CIVD modifiers – Narrative review

A narrative review evaluating the effect of personal factors (age, sex, body dimensions, ethnicity, mental stress, nutrition, pharmacological agents, exercise, and training), ambient factors (water and air temperature), and protocol (prewarming presence, immersion of fingers or hand) on CIVD was performed and evidence was synthesized with the observations from the meta-analysis.

### Personal factors: Age

The efficiency of thermoregulatory mechanisms is reduced with increasing age [108]; such as reduced temperature responsiveness [109], weakened vasomotor response [110], and decrements in sympathetic neurotransmission [111]. It has been observed that those 45 y and older show a steep decline in oral temperature compared to younger individuals when exposed to a two-hour cold exposure (10°C), indicating that people of older age have more difficulties in maintaining thermal control in the cold [65]. The mechanisms mediating both reflex and local cutaneous vasoconstriction and vasodilation are impacted by age [112] due to changes in local intracellular signaling.

CIVD onset time has been shown to be significantly longer and the magnitude lower in the elderly compared to younger individuals, likely due to changes in peripheral vascular reactivity to cold stress and morphological and/or functional degradation of the AVAs [113–115]. Furthermore, CIVD amplitude during immersion and finger skin temperature during rewarming are significantly greater in young rural living females compared to older Korean female divers [56]. Additionally, the rate at which finger skin temperatures return to pre-immersion levels has been shown to be delayed in older men (62–70 y) compared to younger men (20–29 y) after a 10-minute finger immersion (10°C) [114]. Finger blood flow is also reduced in older individuals at baseline and during cold-water hand immersion [116,117].

Reduced CIVD responses have been attributed to the diminished efficiency of sympathetic vasoconstrictor responses with increasing age [2,118,119]. Aging down regulates post-junctional alpha receptors, decreasing the responsiveness of norepinephrine mediated cutaneous vasoconstriction and increasing reliance on RhoA and Rho kinase pathways [120,121]. Greater activation of the RhoA and Rho kinase pathway suppresses endothelial nitric oxide production, leading to an imbalance between vasodilatory and vasoconstrictor influences and resulting in lower peripheral temperatures during cold exposure. However, some studies indicate that older adults also exhibit blunted vasoconstriction during cold exposure. For example, Yochihara et al. [122] found that finger skin temperatures were significantly higher in 10 elderly females (66–79 y) compared to 10 younger females (20–22 y) during exposure to 10°C for 49 minutes, indicating an impaired ability to reduce heat loss via vasoconstriction in the elderly.

Aging changes the relative contributions of the underlying mechanisms mediating vasoconstriction [113,123], where elderly have a decreased reliance on adrenergic-induced vasoconstriction and a shift toward Rho/ROCK-mediated vasoconstriction [111]. In addition, decreased bioavailability of nitric oxide and age-related increases in oxidative stress may contribute to alterations in thermoregulatory control [124]. Previous data have shown that supplementation with L-arginine for increasing nitric oxide synthesis or perfusion of ascorbate into aged skin restores reflex cutaneous vasodilation and increase cutaneous vascular conductance [124].

In addition to physiological thermoregulatory impairment, the elderly show reduced cutaneous thermal sensitivity [108] and reduced subjective thermal perception during cooling [116,122]. The altered structure and function of the central nervous system, peripheral fiber loss, and lower conduction velocity could result in reductions of thermal sensitivity and a decreased ability to perceive cold [116,125]. This is potentially dangerous because thermal perception and thermal comfort are the factors that initiate behavioral thermoregulation.

Limited data is available regarding children. Consistent with Yoshimura and Iida’s [126] study, Inoue Yoshimitsu et al. [113] showed that young boys exhibited inferior CIVD responses compared to adults [113,126]. Children show poorer CIVD than adults, but better than during puberty [126]. Additionally, T_mean_ and T_max_ during immersion were lower in boys compared to adults [113]. Moreover, Miller and Irving [127] observed that the finger temperature of Eskimo children decreased more than that of adults when exposed to cold temperatures [127]. Taken together, the limited studies available on finger blood flow in children exposed to cold indicate an inferior response, and therefore, likely a higher risk for cold injuries. However, this cannot be substantiated since epidemiological data on cold injuries in children is lacking, to the best knowledge of the authors.

To summarize, aging is associated with 1) changes in intracellular signaling, 2) impairments in endothelial and vascular smooth muscle second-messenger signaling, 3) decreased vasomotor responsiveness to norepinephrine, and 4) decreased subjective thermal perception leading to reduced CIVD responses in the cold and slower rewarming. These results are in line with the observations of the moderator analysis that onset time declined with age. However, the meta-analysis showed that temperature responses of CIVD were not affected by age. It is worth noting that the age group of 20–30-year-olds was overrepresented in the meta-analysis, and data on the elderly and children were lacking.

### Personal factors: Sex

Women experience a higher increase in vasomotor tone and thus have lower finger and hand blood flow than men in thermoneutral conditions [2,128]. However, it has been shown that the lower resting skin blood flow in females disappears in postmenopausal women, potentially due to changes in female sex hormones [129].

Estrogen has a direct effect on blood vessel walls; therefore, it is frequently used as a cardioprotective mechanism [130]. The impact of estrogen on the cutaneous microcirculation was evaluated on the forearm using 1% acetylcholine chloride (endothelium-dependent vasodilation due to the release of nitric oxide) and 1% sodium nitroprusside (endothelium-independent vasodilation due to relaxation of smooth muscle cells) through iontophoresis [131]. It was found that both endothelium dependent and independent vasodilation were greater during the midcycle phase (10–14 d after menses) than during the menstrual phase. Estrogens increase vasoconstriction, possibly by stimulating endothelial nitric oxide synthesis [131], up-regulation of vasoconstrictive alpha2-adrenoreceptors [132], antioxidant properties [133], stimulation of prostacyclin, direct influence on the vascular wall, inhibition of prostanoids, and alteration of calcium flux in vascular smooth muscle [131]. Previous studies have shown changes in the number of alpha 2-adrenergic receptors during the menstrual cycle [134], while others have not [135].

Further work demonstrating the influence of hormonal status on peripheral circulation has shown that after a finger cooling test, the lowest T_mean_, lowest finger blood flow, and slowest rewarming were found in the luteal phase compared with the pre-ovulatory phase [136]. On the contrary, Sugawara et al. [91] found that T_mean_ and CIVD onset time were shorter in the luteal phase compared to the late follicular phase in 24 women [137]. Figure 7 illustrates the menstrual phases, associated hormone levels, and CIVD responses. Bartelink et al. [138] evaluated the influence of hormonal status on skin blood flow within and between males, pre-menopausal women (during day 3, 4, or 5 of menstruation because skin blood flow and hormone levels are the most stabilized [136,139]), postmenopausal women, and women using oral contraceptives (OC) (during any of the active pill phase days 10–21). During the 15°C finger cooling test, finger skin temperature and blood flow were higher in males compared with OC women and premenopausal women [138]. Additionally, finger skin temperature was higher in postmenopausal women compared to OC women. Similar results have been found during rewarming from finger cooling tests, where males display higher finger skin temperature and blood flow [138,140]. The slower rewarming rate of females may have been due to estrogens prolonging vasoconstriction by up-regulating vasoconstrictive alpha-adrenergic receptors [138].

Freedman et al. [141] evaluated the interaction of circulating catecholamines with alpha- and beta-adrenergic receptors. They found that women have lower sensitivity and/or density of peripheral vascular adrenergic receptors [141]. This may explain why females have been shown to have a faster rate of fingertip skin temperature cooling when exposed to contact cooling across different types of materials [142]. Furthermore, female skin cools significantly faster during 0°C cold air exposure, suggesting that they may be at a higher risk for cold injury to the fingers [143]. However, Miller and Irving [127] found no finger skin temperature differences in cold air between Eskimo women and men [127].

In contrast, a greater paradoxical vasodilation response (greater femoral blood flow and vascular conductance) has been found in young women to a cold pressor test compared to young men [144]. Regarding the feet, Tsoutsoubi et al. [95] found that women had more CIVD reactions in the toes compared to males during a 40 minute foot and hand cold-water immersion (8 ± 1°C) [95].

Previous investigations have found no direct relationship between hand and finger blood flow/vascular reactivity and levels of circulating estrogen and progesterone [138]; [128,145] and no effects of sex on CIVD in the fingers [95,97,127,146]. Additionally, it has been previously shown that no intra-menstrual differences were found after cooling the feet of males and females during both the inactive and active pill phases [147] or while measuring CIVD in hands exposed to 0°C cold air [143].

Recent studies have suggested that differences in hand anthropometrics may be the cause of sex differences in CIVD responses. For instance, small hand volume shows an increased tendency toward more cold-induced vasoconstriction [148]. The fingers and feet of females cool at a faster rate than those of males, which may be due to their smaller hands and feet [142,147,149], as their smaller dimensions provide lower heat content and consequently faster skin cooling. Conversely, during a 5-minute cold water hand immersion (15°C), hand volume did not relate to CIVD or rewarming responses [138]. To determine if sex-related differences are due to the differing dimensions of the hands and feet, anthropometric matched groups of males and females are needed. Weller et al. [150] constructed a male and female subgroup of identical anthropometrics and found no differences between any CIVD parameter (T_mean_, T_max_, T_min_, and onset time), while for the entire sample, individuals with smaller surface-to-mass ratios exhibited enhanced CIVD responses. These results suggest that differences in body dimensions between sexes likely play a larger role in modulating CIVD responses than sex alone.

In addition, while some studies have observed a higher incidence of non-freezing cold injuries in females than males during cold water paddling [151], others have not found any CIVD differences between sexes [95,97,144]. Whether these differences in peripheral blood perfusion and temperature in the cold between women and men may be linked to increased risk of cold-weather injuries remains to be established [152].

In summary, women generally exhibit lower finger skin temperatures and hand blood flow in thermoneutral conditions, with female sex hormones playing a role in modulating vascular responses to cold. Estrogen’s vasoregulatory effects contribute to prolonged vasoconstriction, potentially leading to slower rewarming rates. Furthermore, the moderator analysis indicated that onset time was shorter and T_min_ was lower for females compared to males. While some studies support these findings, others report no significant sex differences, emphasizing the need for further research on the interplay between hormonal status and anthropometrics in sex differences.

### Personal factors: Body dimensions

Hand and finger dimensions may contribute to the significant interindividual differences found in CIVD responses [153]. “Allen’s rule” is an ecogeographical principle that describes homeothermic animals living in cold climates as having shorter and thicker limbs and extremities, which provide a favorable thermal adaptation in such environments [154]. The high surface area-to-volume (SA:V) ratio predisposes the hand to heat loss, thus increasing the risk of cold injury [155–157].

Hand volume [149], palm size [149], and finger size [158] have been shown to be predictors of skin cooling when measuring contact cooling against metal surfaces. Jay and Havenith [142] measured finger skin cooling on contact with cold metals using the index finger at surface temperatures of −2°C and −10°C. Two groups were identified as either slow coolers (>45 seconds to reach a contact cooling temperature of 1°C) or fast coolers (<25 seconds to reach a contact cooling temperature of 1°C). Under slow cooling conditions, a larger index finger provided a higher heat content, thus giving a slower cooling speed; however, for the fast coolers, hand and finger size did not have any significant correlations. Similarly, after 3 minutes of ice water immersion of the hand, index finger width was correlated with post-immersion average digit temperature, where wider index fingers displayed higher skin temperature values [156].

During the initial 5 minutes of cooling in 8°C water, average finger temperature has shown a significant, negative correlation to digit surface area to volume (SA:V) ratio (R^2^ = 0.06), however the low R^2^ values suggest that other factors besides digit anthropometry likely predominate in predicting finger temperature [102]. In agreement, hand size as measured by water displacement and amount of subcutaneous fat on the hand has not shown any significant correlations to any CIVD components after 30 minutes immersion in 5°C water; however, the small sample sizes make correlation analyses difficult to interpret [85].

Regarding rewarming responses after cold water exposure, longer and narrower fingers have correlated with slower rewarming, while shorter and broader fingers have shown faster rewarming responses following a 3-minute cold water hand immersion (0°C water) [156]. Conversely, after 30 minutes of 8°C immersion, Wickham and Cheung [102] found no relationship between the SA:V ratio and average finger skin temperature during 10 minutes of rewarming.

A predictive model utilizing different hand sizes with varying SA:V ratios while wearing a windproof mitten at −20°C found that even with appropriate outdoor clothing, smaller hands were shown to be more prone to reach skin temperatures associated with dexterity loss or cold injury [153]. This is possibly due to the large SA:V ratio, minimal muscle mass [102], minimal fat mass [157,159], and the potent vasoconstrictor capacity of the fingers [102] leaving the hands and fingers poorly insulated and more susceptible to heat loss [156].

Additionally, as core temperature plays a major role in CIVD responses, and hand SA:V ratio has been shown to impact core temperature [160], it is further likely that hand SA:V ratio can influence peripheral responses. Payne et al. [156] conducted a 3-minute ice water hand immersion on 114 volunteers. They found that skeletal muscle mass was the strongest predictor of heat loss from the digits [156], likely due to the greater thermogenic capacity of muscle mass [161]. Bergmann’s rule supports these results, where larger body size animals (smaller SA:V ratio) are predominantly found in cold environments, while smaller size animals, presumably with less muscle mass, inhabit warm environments [162].

In summary, wider/larger hands and shorter/wider fingers (specifically the index finger) may improve heat retention in the periphery during cold stress. However, there is insufficient evidence to determine the extent to which hand and finger anthropometrics are a strong determinant of CIVD responses. The moderator analysis indicated that onset time increased with body surface area and that higher surface-to-mass percentages were related to lower T_min_ values. Populations in tropical areas are known to have a larger surface-to-volume ratio than cold-indigenous people, and thus ethnicity or geographical location may interfere with body dimensions [163], making it difficult to draw conclusions. Future CIVD studies should consider including both males and females from a single geographical location with similar body dimensions to separate sex from anthropometry and avoid ethnicity bias.

### Personal factors: Adaptation of CIVD

Individuals living in cold climates often display more robust CIVD responses than those from warmer regions, indicating possible adaptation to their environment [164]. However, laboratory and field studies evaluating the ability to enhance CIVD through repeated local cold exposures generally show minor or no improvement over time, raising questions about its potential to mitigate cold injury risks.

#### Short-term exposure/laboratory studies

Laboratory studies offer a controlled environment for exploring the trainability of CIVD, but findings are largely inconsistent. Early research demonstrated some evidence of adaptation [165], such as faster onset times and higher finger skin temperatures after immersing the right index finger in ice water for 20 minutes, 4–6 times daily for 1 month [166]. More recent studies have revealed that 2 wk of repeated hand immersions in 8°C water led to increased T_min_ and T_mean_ of the index finger, alongside decreased minimum skin temperatures of the hand, with no differences between exercising and resting during immersion [38]. This region-specific response, with warming in the fingertips and cooling in the larger hand muscles likely reflects the localized nature of CIVD, where rapid cooling and subsequent cold-induced paralysis of blood vessels result in stronger CIVD events in the fingertips [2].

However, other studies have shown minimal [61] or no significant changes in CIVD parameters despite extended immersion protocols [31,167]. Some research even reported reduced CIVD responses, attributed to heightened sympathetic activity following repeated exposures, such as immersions in ice water for 15 minutes daily over 14 d [31], 8°C water for 30 minutes daily over 13 d [167], or 5 d a week for 3 wk [36]. For instance, Geurts et al. (2005) found that cold acclimation not only failed to produce favorable adaptations, but might increase the risk of cold injury, as T_min_, T_mean_, and amplitude decreased, while onset time increased compared to controls (Geurts et al., 2005). Similarly, Mekjavic et al. [167] observed a decrease in CIVD waves and mean temperatures after 13 d of daily hand immersions in 8°C water [167]. Others reported no changes in CIVD response following repeated immersions of the foot in 8°C water for 30 minutes, 5 d/week for 3 wk [168].

Physiological changes during whole-body cold exposure can influence local responses. For example, during whole-body acclimation protocols, such as 1-hour immersions in 20°C water over 5 wk, reduced finger skin temperatures were found during 4°C finger-only immersions [169]. This suggests that central nervous system adaptations might suppress CIVD responses (Livingstone, 1976 [170]). These findings indicate that whole-body cold adaptations can override localized vasodilation. In contrast, repeated local cold exposure of the lower limbs (twice daily, 5 d/week for 1 month) induced hypothermic insulative adaptations. These were characterized by decreased core and skin temperatures during whole-body cold exposure after local acclimation, and higher skin temperatures of the feet during lower limb immersion [171]. Such systemic effects may arise from altered thermoregulatory processes and thyroid hormone dynamics, rather than localized skin temperature changes or habituation to the cold.

In summary, while some protocols show limited evidence of adaptation, the general trend is a lack of meaningful laboratory-based acclimation.

#### Long-term exposure/field studies

Longitudinal field studies emphasize the ecological complexity of CIVD trainability, with findings often varying based on environmental and individual factors. For example, tropical soldiers exposed to Arctic conditions for 7 wk demonstrated moderate improvements in CIVD, though their responses remained inferior to those of Arctic natives [77]. In contrast, SCUBA divers in Antarctica displayed no significant enhancement in CIVD compared to non-divers during monthly index finger immersions in ice water, despite a year of extreme cold exposure. While these divers did not experience hypothermic core temperatures during dives, they underwent repeated substantial peripheral cooling, yet peripheral acclimatization was absent [27]. Similarly, previous research on non-divers living in Antarctica has shown that men working in such environments do not develop peripheral adaptations to cold exposure [172].

Furthering this inconsistency, Livingstone (1976) observed lower T_mean_ and T_min_, as well as delayed onset times in Canadian soldiers after a 2-wk Arctic expedition with diurnal temperatures ranging from −10°C to 40°C. These findings suggest that short-term cold exposure may increase vasoconstrictor tone and promote general, rather than peripheral, acclimation.

The results of prolonged Antarctic expeditions also vary widely. Some studies have reported no change in CIVD [173] or even reduced finger skin temperatures in 5°C air compared to controls [174]. Conversely, others found enhanced CIVD responses and finger temperatures during extended cold exposure [77,175]. For example, during an eight-month Antarctic expedition, participants showed improvements in T_mean_, T_min_, T_max_, CIVD amplitude, and onset time during 30-minute immersions of the index finger in 0°C water [96].

Overall, while long-term extremity cold exposure has the potential to elicit CIVD adaptation under certain conditions, it does not appear that there is sufficient evidence that CIVD is trainable from long-term field studies. Findings do remain inconsistent and likely depend on a combination of exposure duration, environmental stressors, and individual variability.

#### Ecogeographical adaptation (population and genetic studies)

Ethnic groups native to Arctic regions are assumed to have greater exposure to both general and localized cold compared to those from tropical areas, potentially driving genetic or physiological adaptations over generations. CIVD has also shown differences between Africans and Caucasians as the CIVD response occurs more in Caucasians, and Africans exhibit a more intense and prolonged finger vasoconstriction [60].

Population studies reveal pronounced differences in CIVD across ethnic groups, likely influenced by both environmental and genetic factors. Arctic natives, such as the Inuit, display enhanced CIVD responses with higher T_mean_ and faster onset times compared to temperate populations [176], while tropical populations exhibit weaker responses. However, studies on occupational groups, like fishermen, provide conflicting evidence of CIVD enhancement, likely due to self-selection biases and varying degrees of local versus whole-body cold exposure [177]. Table 8 taken from [27] highlights the influence of habitual cold exposure.

**Table 8.** Table reproduced with permission from Bridgman [27] comparing the onset time of CIVD across various populations and occupational groups, highlighting differences in cold adaptation. The table was extended to include studies published after 1991 comparing CIVD responses in different populations, however studies were only included in the chart if onset time was reported. H = hand; IF = index finger; LF = little finger; MF = middle finger; *SD* = standard deviation; ns = not stated.

Participants (Reference)	*n*	Part Imm	Water temp (°C)	Fingers measured	CIVD onset (min + SD)
UK fish filleters [178]	10	H	0	MF fingerpad	4.5 ± 1.5
Arctic Indians [179]	9	H	0	IF fingerpad	4.7 ± ns
Japanese fishfactory workers [180]	9	MF	0	MF nailbed	4.7 ± 1.7
Finnish Lapps [181]	10	ns	ns	ns	5.0 ± 1.2
Norway Lapps [182]	40	H	0	MF nailbed	5.4 ± 3.7
Japanese farmers [180]	8	MF	0	MF nailbed	5.4 ± 1.6
UK fish filleters [178]	16	H	0	MF base	5.5 ± 1.9
Aimu [180]	9	MF	0	MF nailbed	5.5 ± 0.7
Japanese students [180]	148	MF	0	MF nailbed	6.2 ± 4.8
Japanese policemen [180]	13	MF	0	MF nailbed	6.5 ± 1.4
Norway fishermen [182]	14	H	0	MF nailbed	6.9 ± 2.2
Caucasian post laboratory acclimation [166]	8	IF	0	IF fingerpad	7.0 ± ns
Caucasians [179]	10	H	0	IF fingerpad	7.5 ± ns
Japanese fishermen [180]	4	MF	0	MF nailbed	7.6 ± 0.9
Japanese Main Island [180]	7	MF	0	MF nailbed	7.8 ± 0.7
Caucasians [166]	5	IF	0	IF fingerpad	7.9 ± ns
Caucasians [183]	5	H	5	LF nailbed	8.4 ± ns
Japanese [184]	9	MF	0	MF nailbed	8.7 ± ns
Nondivers [27]	6	IF	0	IF fingerpad	9.1 ± 2.5
Caucasians [182]	11	H	0	MF nailbed	9.1 ± 4.4
Caucasians [185]	7	MF	0	MF fingerpad	9.2 ± ns
Canadian soldiers [58]	8	MF	0	MF fingerpad	9.3 ± 0.8
Divers [27]	14	IF	0	IF fingerpad	9.4 ± 5.5
British [178]	24	H	0	MF base	9.7 ± 6.3
British [178]	18	H	0	MF fingerpad	9.9 ± 4.2
Canadian soliders post cold exposure (Livingstone, 1976)	0	MF	0	MF fingerpad	12.4 ± 0.0
Blacks [183]	7	H	5	LF nailbed	12.9 ± ns
Blacks [185]	0	IF	0	IF fingerpad	15.9 ± ns
Caucasians [60]	10	H	8	IF & MF fingerpad	11.4 ± 7.7
Asian [60]	10	H	8	IF & MF fingerpad	9.6 ± 4.8
Blacks [60]	10	H	8	IF & MF fingerpad	16.9 ± 7.0
Korean Haenyeo [56]	22	MF	4	MF fingerpad	4.2 ± 1.7
Korean older non divers [56]	25	MF	4	MF fingerpad	4.8 ± 1.5
Korean young urban dwellers [56]	15	MF	4	MF fingerpad	4.4 ± 0.7
Korean young rural dwellers [56]	51	MF	4	MF fingerpad	5.0 ± 1.1
Korean older non-divers [52]	10	MF	4	MF fingerpad	4.3 ± 1.3
Korean retired Haenyeo [52]	10	MF	4	MF fingerpad	5.7 ± 2.8
Korean active Haenyeo [52]	10	MF	4	MF fingerpad	4.2 ± 3.0
North Chinese [85]	16	H	5	MF fingerpad	5.1 ± 3.2
South Chinese [85]	13	H	5	MF fingerpad	9.9 ± 6.8
Tuvan pastoralist [81]	13	MF	0	MF fingerpad	8.2 ± 4.3
Western European [81]	13	MF	0	MF fingerpad	14.3 ± 4.7

Occupational groups exposed to cold, such as Gaspé fishermen [186], Norwegian fishermen [182], British fish filleters [178], and Korean Haenyeos [56], also exhibit enhanced CIVD. Fishermen, who regularly immerse their hands in water between 9°C and 12°C for hours daily, showed significantly higher finger temperatures and faster vasodilation onset during cold-water immersion than unacclimatized controls [178]. Fish filleters working with one ungloved hand in ice water experienced similarly enhanced finger and hand temperature maintenance. Importantly, these adaptations appear to be region-specific, as Quebec fishermen exhibited no differences in foot temperature during cold-water immersion compared to controls, likely reflecting less frequent exposure of the feet to the cold [187]. However, discrepancies remain. For example, Leblanc et al. [186] and Krog et al. [182] found enhanced CIVD in fishermen, while Hellstrom and Andersen [188] observed no differences. Selection may again have played an important role, as individuals with persistently cold hands would be unlikely to choose a profession such as a fish filleter, which involves repeated exposure to cold.

Korean female divers (Haenyeos) swim for hours in average sea water temperatures of 14–16°C in the winter at Jeju Island, South Korea. Although Haenyeos no longer wear cotton bathing suits, their faces, hands, and feet are continuously exposed to cold water during long dives. Active Haenyeos have showed 1.7°C higher finger skin temperatures at the onset of vasodilation compared to non-divers during finger immersion in 4°C water, and their CIVD cycles appeared twice as often as those of retired Haenyeos [52]. Modern Haenyeos’ cold tolerance demonstrates local hand/finger adaptations to cold and may be related to vasomotor reaction magnitude rather than its speed [56]. Lee et al. [189] found that older Haenyeos showed significantly greater T_min_ and T_max_, shorter onset and peak time, and greater frequency of CIVD during the finger immersion test in 4°C water when compared to young urban females [189]. Older Haenyeos showed more pronounced T_min_ during the finger cold-water immersion than older non-diving females, further highlighting that they do retain their cold-acclimatized features despite switching to wetsuits decades earlier [56,190].

Similar CIVD responses have been observed in native populations habitually exposed to the cold, including indigenous Arctic groups [191,192], Inuits [127,187], Yukon Native Americans [179], Lapps [182] and Manchurians [126]. Genetic factors also contribute to CIVD variation. For example, northern Chinese exhibited higher T_mean_, T_min_, and earlier onset times compared to southern Chinese, correlating with colder January temperatures in their region (<2°C vs. >2°C), even when controlling for lifetime and occupational acclimatization [85]. Since the participants were not cold acclimatized, these findings point to a genetic influence on CIVD among Continental Asian populations [85]. Similarly, 13 Tuvan nomadic pastoralists, who reside in yurts and are regularly exposed to winter ambient temperatures between −38.4°C and −15.4°C, showed significantly stronger CIVD responses than 13 Dutch Europeans. At rest, Tuvans had higher finger skin temperatures (34.6 ± 2_C vs. 26.2 ± 4_C, *p* < .001) and higher T_mean_ values (13.4 ± 3.4_C vs. 3.9 ± 2.3_C, *p* < .001) during a 30-minute immersion in ice water, with CIVD occurring in 83% of Tuvans compared to 45% of Europeans [81]. Despite this evidence, most CIVD studies do not report on participant ethnicity. In Western countries, study populations are often students or military personnel, typically of mixed ethnic backgrounds. Given the influence of ethnicity on CIVD, future studies should report on the ethnic background of participants to improve interpretation of results. For instance, differences in CIVD between inhabitants of two neighboring Algonkian Indian villages were attributed to ancestry; one village was primarily Native American, while the other included more individuals of European descent [44].

Factors contributing to ecogeographical adaptations include the potential of enhanced formation of new AVAs in the skin, which relax under cold stress to promote blood flow, and a blunted sympathetic response in Arctic residents, allowing for greater peripheral blood flow and T_mean_ during local cold exposure [177,193]. Additionally, morphological adaptations, such as thicker, shorter fingers that reduce heat exchange [75,194], and the potential role of mast cells, rich in vasodilatory histamine [186], may further enhance CIVD responses, though the genetic versus environmental basis of these mechanisms remains unclear [177].

Overall, individuals in cold-weather occupations and natives to cold regions may develop enhanced cold tolerance due to repeated exposure, though the extent to which this reflects true physiological adaptation versus self-selection remains a topic of interest. Additionally, the moderator analysis found that cold-indigenous populations exhibited shorter onset times of CIVD compared to controls, further supporting the idea that long-term exposure to cold environments may shape vascular responses.

### Personal factors: Mental stress

Hugdahl et al. [195] demonstrated that exposure to a cold room (Ambient: 0°C) combined with mental stress (counting backward out loud under threat of electric shock for 15 minutes), led to decreased finger skin temperatures [195]. In agreement, others have found decreases in finger skin temperature in response to a mental stress task [166,196]. In some cases, CIVD responses were completely abolished following acute stress. For example, one participant who consistently displayed CIVD in three earlier trials showed no response after completing a stressful exam [197]. In another study, students exposed to varying levels of shock intensity after a 15-minute cold pressor test displayed stress-dependent changes; those in the highest shock intensity group had increased CIVD latency [198], suggesting an increase in sympathetic nervous system activation [199].

Although mental stress is generally associated with cutaneous vasoconstriction [200], paradoxical vasodilation has been observed under certain conditions. Cooling participants in a thermal suit while inducing mental stress produced vasodilation rather than vasoconstriction [201–203], particularly when baseline hand blood flow was already low [201]. This paradoxical response may occur because AV shunts are already closed by the cold, limiting further constriction, and rendering stress-induced effects less apparent [204]. More recently, Moes [205] reported that mental stress applied prior to hand immersion reduced CIVD, whereas stress applied during CIVD had no effect [205].

Links between mental stress and vasospastic attacks have also been described in individuals with Raynaud’s phenomenon where emotional stress and cold exposure can trigger attacks [206]. In one study, mental stress (arithmetic calculations) and mild cold (20°C and 25°C) produced contrasting effects: the control group displayed reductions in fingertip blood flow during mental stress while those with Raynaud’s experienced vasodilation [207]. However, when individuals with Raynaud’s were shown a stressful scene specific to their ailment, such as being stuck in a snowstorm with lost gloves and car keys, finger skin temperature decreased [208].

While performing mental arithmetic at rest in thermoneutral conditions, a decrease in fingertip blood flow [209] and reduced hand blood flow and skin perfusion in men [128,210] has been observed. Mental arithmetic increases the skin’s sympathetic adrenergic vasoconstrictor nerve activity, which decreases finger and hand blood flow [211]. The impact mental stress has in thermoneutral conditions on hand blood flow may be dependent on sex differences since only males experienced reduced hand blood flow while females had a paradoxical vasodilation [128]. The increase in hand blood flow in females may have also been due to a decrease in hand vascular resistance.

Overall, mental stress generally decreases finger skin temperature and reduces finger and hand blood flow under both cold and thermoneutral conditions. However, the effects are highly variable, depending on factors such as the type, intensity, duration, and individual differences in stress response. Due to this variability and the challenge of standardizing mental stress across studies, it was not included as a moderator variable in the meta-analysis.

### Personal factors: Nutrition

There are only a few studies that have investigated the influence of chronic and/or acute nutritional interventions on CIVD responses.

It is known that diet-induced thermogenesis increases body temperature by sympathetic activation of heat production [212] and due to the increase in metabolic rate to digest food. Takano & Kotani [213] evaluated the influence of consuming food on CIVD [213]. Twelve female students completed a finger-cooling test after an overnight fast. After the first finger cooling test, a 700-kcal meal with 36% carbohydrates, 48% fats, and 16% proteins were consumed. About 30 minutes later, 2 additional tests were conducted with approximately 1 hour in between. It was shown that the CIVD index (defined as the area under the curve from T_onset_ to end of test) significantly increased from 5.4 ± 1.4 pre-prandial period to 9.1 ± 2.2 at 30 minutes and 8.1 ± 1.6 at 90 minutes of the post-prandial period. These results showed that even small increases in heat due to the thermic effect of food can mediate CIVD responses.

It is not only dietary-induced thermogenesis that increases body temperature, but the temperature of the food itself may have an impact. Drinking hot beverages has been shown to produce an immediate peripheral vasodilation that can last up to 40 minutes [214]. Goldman et al. [214] provided 10 males 200 ml of 55°C bouillon beverage 60 minutes prior to a 60-minute hand and foot immersion (15°C water). During the first 15 minutes, the 3 digits (1^st^, 3^rd^, and 5^th^) skin temperature after consuming hot bouillon was higher than control (200 ml of 23°C water) and significantly reduced heat loss after 30 minutes. No significant differences were found in the foot (number of CIVD cycles in the toes).

The amount of heat production from the thermic effect of food and the impact on CIVD may also rely on diet composition. The following sections will review the impact different macronutrients and micronutrients have on CIVD.

#### Macronutrients

Van Marken, Lichtenbelt & Daanen (2003) reviewed the impact diet has on cold-induced metabolism and found that the protein content has a strong relationship to heat produced from increases in energy expenditure [2,215]. Specifically looking at peripheral responses, Yoshimura et al. [216] showed that chronic intake of protein (150–200 g/d) or salt (>45 g/d) daily for 1–2 wk improved the hunting reaction and resistance index to frostbite score due to the increase in basal metabolism leading to increased peripheral blood flow [137,216].

The role of carbohydrates and fat in CIVD responses is less clear. To our knowledge, there have not been any studies investigating high vs low carbohydrate diets or high vs low fat diets on CIVD in humans.

#### Micronutrients

The previous review [2] highlighted the impact of vitamin C on CIVD components. Vitamin C (ascorbic acid) has been shown to improve peripheral vascular responses during local cold stimulus [78,217–219]]. Livingstone (1976) found that after providing 2000 mg of vitamin C daily for 30 d, CIVD improved due to a shortened onset time [59]. In agreement, LeBlanc et al. [217] found that daily 525 mg vitamin c supplementation for 13 d compared to control (25 g vitamin C) greatly improved average skin temperature responses in men during concurrent stressors: cold exposure and fed a survival ration of 550 calories/day (jelly candy). Supplementation with vitamin C may augment CIVD due to its antioxidant, metabolic, and thermogenic properties [2].

In recent years, the most frequently studied nutritional strategy to improve CIVD responses is acute beetroot juice supplementation [103,164]. Beetroot juice contains vasodilating substances such as dietary nitrate (NO_3_^−^), betanin, quercetin, and chlorogenic acid that may play a role in improving peripheral re-warming [220,221].

Wickham et al. [103] found that ingesting 140 mL of nitrate-rich beetroot juice 2 hours prior to cold water hand immersion had no effect on finger skin blood flow or any CIVD parameters during immersion. Wakabayashi et al. [99] found similar results where beetroot juice did not influence CIVD responses during cold water hand immersion, however the rewarming rate in finger skin temperature and skin blood flow was significantly faster with individuals who consumed 140 mL of beetroot juice (800 mg NO_3_^−^ and 154 mg betanin) 2 hours prior to cold exposure. In summary, beetroot juice seems to impact rewarming responses, but not CIVD.

The effect of beetroot juice on CIVD during immersion and rewarming may be influenced by individual differences in cold sensitivity. Wakabayashi et al. [99] found that cold-sensitive individuals who consumed the control (140 mL water) had slower rewarming rates, however with beetroot juice supplementation the same cold-sensitive individuals demonstrated significantly faster rewarming rates. It is worth noting that blood samples were not collected to verify increases in plasma NO_3_^−^ and NO_2_^−^ for both studies evaluating beetroot juice supplementation.

In summary, studies suggest that dietary-induced thermogenesis and the temperature of consumed food can influence peripheral vasodilation and CIVD. Protein intake appears to enhance CIVD, likely due to increased basal metabolism, while vitamin C supplementation has been linked to improved peripheral vascular responses. Conversely, beetroot juice supplementation has shown no direct impact on CIVD during cold immersion but may aid rewarming. Despite these findings, the research primarily focuses on acute nutritional effects in male participants, with limited exploration of chronic dietary influences.

Nutrition was not included in the moderator analysis due to the scarcity of studies, the lack of controlled comparisons across macronutrient and micronutrient groups, and the absence of consistent findings across different populations and exposure conditions.

### Personal factors: Pharmacological agents

Individuals suffering from Raynaud’s syndrome/phenomenon or diabetes; those under prescription medication for hypertension or cardiovascular disease; and those who have recently taken anesthesia agents, caffeine, alcohol, insulin, menthol, morphine, nicotine, sedatives, l-menthol, sublingual glyceryl trinitrate, and/or other drugs may have impacted CIVD responses. Certain pharmacological agents influence thermoregulatory responses, therefore affecting vasoconstriction and/or vasodilation in the periphery.

#### Anesthesia & sedation

During anesthesia and/or sedation, it is common for core temperatures to drop inducing mild hypothermia due to the reduction in shivering rate in turn enhancing cooling for patients [222]. Drops in core temperature as small as only a few tenths of a degree can cause changes in vasomotion; therefore, it is reasonable to assume CIVD would be impacted by anesthesia and sedation [222,223]. Since hypothermia frequently occurs following spinal anesthesia, Kurz et al. [223] evaluated thermoregulatory responses with seven healthy women with and without spinal anesthesia [223]. Spinal anesthesia was found to significantly decrease vasoconstriction and increase shivering thresholds by 0.5°C. The initiation of shivering at a lower core temperature enhances cooling rates due to the inability to produce metabolic heat. Sedation and anesthesia (general and regional) promote heat loss through an anesthetic-induced peripheral vasodilation by impairing hypothalamus thermoregulation and altered afferent input [222].

#### Caffeine

CIVD is influenced by the thermal state of the body, and caffeine has been shown to increase thermogenesis during rest in thermoneutral conditions [2,224,225]. Therefore, Kim et al. [226] investigated the effects of acute caffeine intake (300 mg caffeinated chewing gum) on CIVD responses [226]. Control trials included sitting for 10 minutes without consumption of any caffeinated or noncaffeinated chewing gum. Caffeine consumption prior to peripheral cold exposure negatively impacted CIVD responses: a lower T_max_ and T_mean_ was observed for those chewing caffeinated gum compared to control. Factors that may have contributed to lowered skin temperatures after caffeine consumption include increased norepinephrine and a caffeine-induced block of adenosine receptors, resulting in reduction of peripheral blood flow [227]. Norepinephrine has been shown to stimulate α- adrenergic receptor activity triggering vasoconstriction and decreased CIVD responses [228].

#### Beta blockers

Cold environments are associated with higher cardiac symptoms (angina, arrhythmias, dyspnea), hypertensive crisis, heart failure, angina pectoris, and sudden cardiac deaths in both healthy and persons with cardiovascular diseases [229]. Individuals with cardiovascular disease typically also present with hypertension. Beta blockers are a common medication that functions as an anti-hypertensive agent. Nebivolol, a beta blocker, has been shown to increase nitric oxide bioavailability, therefore producing endothelium-dependent vasodilation [230]. For example, nebivolol promoted vasodilation by increasing forearm blood flow in both normotensive and hypertensive individuals after infusing 354 micrograms/minutes into the brachial artery.

Sodium nitroprusside is a potent vasodilator used for lowering blood pressure [231]. Morris & Shore [232] used an iontophoretic application of sodium nitroprusside on the forearms of healthy volunteers and found that forearm blood flow significantly increased for the sodium nitroprusside site compared to the placebo [232]. Additionally, carvedilol and atenolol are beta-blocking agents also used to treat hypertension. Klemsdal et al. [233] provided 25 mg carvedilol or 50 mg atenolol prior to immersing normotensive participant hands in ice-water for 60 sec [233]. Both groups showed an increase in finger skin temperature at baseline and a sharp rise after cold-water hand immersion.

#### Insulin

Individuals with type 1 and 2 diabetes suffer from endothelial dysfunction that impairs vasodilation and vasoconstriction [234,235]. Postprandial and fasting high concentrations of glucose in the blood plasma is responsible for vascular dysfunction due to the inhibition of NO synthase and NO production [235,236].

With limited evidence, it could be suggested that diabetics are at a greater risk of weaker CIVD responses. However, insulin infusion has previously shown to be a potent vasodilator [237].

#### Nicotine & smoking

A previous review [2] highlighted that smoking led to acute peripheral vasoconstriction and inhibits vasodilation [2,238,239], however smokers who abstain from smoking prior to investigations appear to have an enhanced hunting response compared to nonsmokers [2,240]. Acute peripheral vasoconstriction from smoking may be caused by an increase in skin sympathetic nerve activity [241], while chronic smokers show increased skin circulation and vasodilation during periods of smoking cessation [242]. The acute effects of smoking 2 cigarettes prior to 60 minutes cold-water hand immersion (15°C) with 1 hand and 1 foot immersed resulted in hand heat loss from 15.1 ± 1.9 (control) to 11.6 ± 1.7 watts (smokers) and fewer CIVD cycles in the smokers [214]. Smoking had no impact on foot responses.

Acute smoking and nicotine administration also negatively impacts rewarming rate and has been shown to delay the onset of CIVD [57,242]. This may be due to the increase in circulating plasma catecholamines and impairing nitric oxide bioavailability [57]. The effects of vasoconstriction may be noticeable up to 1 hour. However, Usuki et al. [243] found that chewing nicotine gum (2 mg) for 15 minutes immediately increased cutaneous blood flow and skin temperature compared to control [243].

The effect of chronic smoking on CIVD responses after a period of abstinence appears to increase skin circulation so that hand and skin temperatures are higher compared to nonsmokers and may display faster CIVD onset times [2]. The number of years of smoking or age may have an impact as well. Miland & Mercer [242] examined the effect of a 12 hour abstinence from smoking in young and old habitual smokers on rewarming rates [242]. The older habitual smokers had a faster rewarming rate (9.7 ± 0.8 minutes) than the older nonsmokers (16.7 ± 2.6 minutes) and the older habitual smokers had higher baseline hand skin temperatures. There were no differences between young smokers and young nonsmokers. Results may be due to an increase in peripheral vasodilation following abstinence in smokers, which is likely caused by an increase in inflammatory cytokines, upregulation of alpha 2 receptors, and/or autoimmune responses [242,244].

Inconsistencies among the impact of smoking/nicotine on CIVD responses warrants further investigations.

#### Alcohol

The peripheral vasodilator effect of alcohol has been utilized since World War II where soldiers were given alcohol daily [214]. It has previously been shown that alcohol ingestion can increase peripheral blood flow and elevate finger skin temperature [214,245]. Keatinge and Evans [246] studied the effects of alcohol ingestion (59.2 g) on the finger blood flow before and during 30 minutes cold-water hand immersion (15°C). The alcohol trial showed significantly higher finger blood flow during pre-immersion and no differences between alcohol and non-alcohol trials during immersion [246]. Furthermore, Johnston et al. [247] found that participants with a mean blood alcohol concentration of 0.097 ± 0.010 at the start of whole-body cold-water immersion (28°C) and 0.077 ± 0.0008 at the end of cooling showed elevated fingertip blood flow [247]. It appears that alcohol may produce strong peripheral vasodilatory effects. However, there is evidence that suggests alcohol can have a negative impact on the periphery [248] or no difference on finger skin temperatures and rewarming rates [249].

#### Tadalafil

Approximately 5% of the population is affected by Raynaud’s phenomenon [250], a condition characterized by excessive cold- or stress-induced vasoconstriction in the extremities. Since individuals with Raynaud’s exhibit exaggerated reductions in digital blood flow, they are sometimes used as a clinical model to examine pharmacological interventions targeting peripheral vasomotor control. Although most CIVD studies exclude individuals with Raynaud’s for safety, previous work has demonstrated that frequent whole-body exposure to cold air (0°C), while simultaneously submerging hands in warm water (43°C) for 10 minutes can increase finger skin temperature by at least 1°C in this population [251].

Tadalafil, a phosphodiesterase 5 inhibitor, enhances nitric oxide’s vasodilating properties [252]. Friedman et al. [252] evaluated the effect of a single dose of tadalafil in control (healthy) and Raynaud’s phenomenon after 60 minutes stepwise cooling exposure to 8°C air. Tadalafil did not increase finger skin blood flow for individuals with Raynaud’s, suggesting limited efficacy in enhancing cold-induced digital perfusion.

#### L-menthol

L-menthol has been routinely investigated for its use as an over-the-counter topical analgesic to reduce hyperthermia [253]. L-menthol is a cutaneous vasodilator that can enhance skin cooling and heighten the perception of cooling [254–256] due to its agonistic properties of the TRPM8 channel [50]. The TRPM8 channel is a cutaneous thermosensor that is a vasoactive stimulus playing a key role in cold signaling [257]. Kim and Lee [50] found that applying 1.5% L-menthol to the hand and forearms enhanced vasoconstriction after the first CIVD response during cold-water immersion of the middle finger (lowered T_max_ and T_mean_ in L-menthol compared to a control without L-menthol application) [50]. The attenuated magnitude of CIVD may have been due to enhanced vasoconstriction caused by stimulation of additional cold receptors by the L-menthol application.

#### Glyceryl trinitrate

Sublingual glyceryl trinitrate administration (400 ug) has been shown to decrease hand rewarming time after cold-water immersion in individuals with Raynaud’s phenomenon/syndrome [258]. Therefore, Hope et al. [259] evaluated if 400 ug of sublingual glyceryl trinitrate compared to 1 drop of peppermint oil mixed with 100 mL of water (placebo) would improve peripheral responses in cold-sensitive individuals [259] after 2 minutes cold-water foot immersion (15°C, stirred). The sublingual glyceryl trinitrate group showed an increased rate of rewarming in the coldest toe and increased blood flow during rewarming compared to placebo. Sublingual glyceryl trinitrate is an endothelial-independent NO donor that may assist in the release of vasoconstrictor tone during the rewarming period for cold-sensitive individuals [259].

To summarize, pharmacological agents can influence CIVD responses, though their effects vary depending on the substance. Evidence suggests that anesthetics and sedatives impair thermoregulation by increasing shivering thresholds and promoting heat loss, leading to decreased CIVD responses. Similarly, nicotine and acute smoking induce vasoconstriction and delay CIVD onset, though chronic smoking may enhance vasodilation following abstinence. Beta blockers and vasodilators, such as nebivolol and sodium nitroprusside, have been shown to increase peripheral blood flow, which could enhance CIVD, but their direct impact on CIVD remains unclear. Caffeine has demonstrated negative effects by increasing norepinephrine activity and reducing peripheral blood flow, thereby lowering T_max_ and T_mean_. Alcohol’s impact is inconsistent, with some studies showing increased peripheral vasodilation and others reporting no effect or even negative consequences. Insulin has known vasodilatory properties, but its specific effect on CIVD remains underexplored, particularly in individuals with diabetes, who already exhibit endothelial dysfunction. Other agents, such as tadalafil, L-menthol, and sublingual glyceryl trinitrate, have been investigated for their vasodilatory properties, but their direct influence on CIVD remains inconclusive due to mixed findings or limited research. Given the wide range of mechanisms and individual variability in response, pharmacological agents were not included in the moderator analysis to maintain sample homogeneity and avoid confounding effects related to dosage, frequency, and underlying health conditions.

### Personal factors: Aerobic exercise

When exercise precedes a CIVD test, the number of CIVD waves in the toes compared to a control trial without exercise was increased, likely due to the exercise-induced rise in core temperature [260,261]. In agreement, after 10 participants cycled for 30-minute at a fixed rate of 400 W of metabolic heat production, T_min_, T_max_, and amplitude measured at the middle finger were higher after submaximal exercise (post-exercise) compared to pre-exercise [262].

The benefit of exercise-induced elevations in core temperatures on CIVD may occur immediately after exercise but not during exercise while the hand is immersed in cold-water [263]. Geurts [38] evaluated index finger skin temperature responses during 2 wk of repeated 30-minute cold-water hand immersion (8°C), while cycling at 50% heart rate reserve versus a seated control. Despite a significant rise in rectal temperature during cycling (0.6 ± 0.2°C), no differences were observed between groups (Geurts et al., 2006). This may be because minimum index finger skin temperature was reached before the elevated core temperature could enhance vasodilation, as exercise and hand immersion began simultaneously. Moreover, exercising muscles compete with the skin for warm blood while exercising in the cold [263]. In contrast, Makinen [264] found that finger skin temperature increased with higher exercise intensity in still and low-wind conditions, though this effect was abolished when wind speed increased (Figure 8).

Fatigue induced after high-intensity exercise may negatively impact CIVD. O’Brien and Young [169] observed that onset time was reduced after 5 d of repeated strenuous exercise when not allowing for sufficient rest between bouts [265]. Chronic fatigue results in increased circulating norepinephrine that reduce effector sensitivity to sympathetic stimulation [265].

In summary, acute exercise enhances CIVD in both the fingers and toes due to elevations in core body temperature. However, the exercise-induced elevated core temperature may only be beneficial immediately after exercise and not during (particularly depending on ambient conditions).

### Personal factors: Aerobic training

Aerobic training improves vasomotor responses to thermal stimuli [266], increases micro circulation [49], and increases metabolic heat production leading to improved cold tolerance [267]. Sugawara et al. compared 15 athletes and 9 non-athletes and found that athletes finger skin temperatures were higher during cold-water hand immersion compared to non-athletes due to an increased release of histamine substances/vasodilators and reduction of vascular sensitivity to catecholamines [91].

Twenty healthy males completed daily 60-minute cycling training sessions for 10 d and were assigned to either a heat acclimation group who conducted cycling sessions in a 35°C environmental chamber, heat and hypoxic acclimation group who cycled in a 35°C environmental chamber while confined to the hypoxic facility, and a normoxic thermoneutral group who cycled in a 24°C environmental chamber without confinement [29]. Measurements of the CIVD response were conducted 2 d before and 2 d after each 10-d protocol (30-minute whole hand immersion 8°C followed by 15-minute recovery). Ten days of exercise at thermoneutral and the combined stressors of hypoxia and heat did not have any effect on CIVD. Compared to pre-training, the heat acclimation groups average temperature of all five fingers was higher (from 13.9 ± 2.4 to 15.5 ± 2.5°C; *p* = 0.04), 15-minute recovery (from 22.2 ± 4.0 to 25.9 ± 4.9°C; *p* = 0.02), greater number of waves in the thumb, index, and middle finger, and onset time for the pinky was reduced by 4 minute after 10 d of training [29]. Furthermore, exercise increases core body temperature and has been considered a form of heat acclimation.

**Figure 9.**
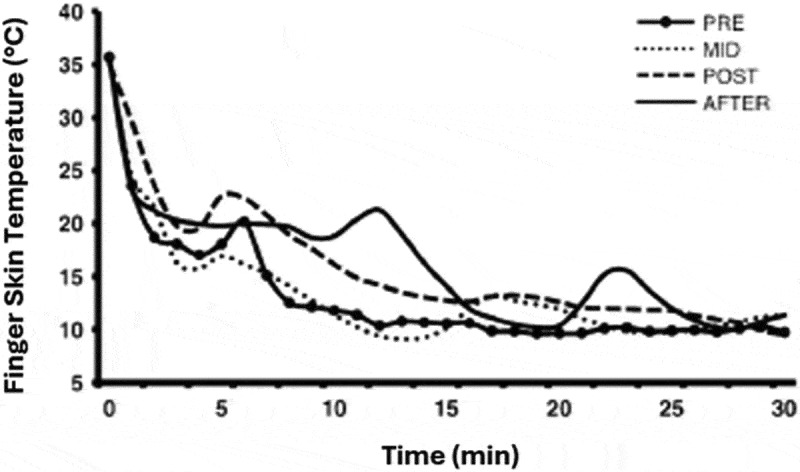
Representative CIVD responses of a subject’s index finger gradually increasing over time during cold-water hand immersion pre, mid, post, and after 4 wk of cycling training. Figure reprinted with permission [49].

Additionally, cycling at 50% of peak power output at a constant cadence of 70 rpm for 5 d/wk for 4 wk increased the number of CIVD waves and T_mean_ [49] (Figure 9). This is in agreement with the previous study by Moriya and Nakagawa [268] who found a significant correlation between absolute maximum oxygen uptake (l/minute), T_mean_, and T_max_ [268].

Aerobic exercise influences CIVD through both acute and chronic mechanisms. Acutely, exercise can enhance CIVD in fingers and toes due to elevated core temperature, though this effect is mainly observed post-exercise since concurrent exercise during cold exposure diverts blood flow to working muscles, reducing its impact on CIVD. Over longer periods, aerobic training induces cardiovascular and neural adaptations that improve vasomotor responses and microcirculation, with heat training providing greater enhancement than normothermic training. However, repeated strenuous exercise without sufficient recovery may impair CIVD due to heightened sympathetic activity.

Exercise was not included as a moderator due to inconsistent effects across studies. The impact of acute exercise varies based on timing (pre-exercise vs. during exercise), environmental conditions (wind exposure, cold air vs. cold-water immersion), and exercise intensity. Similarly, the effects of chronic aerobic training are influenced by training duration, acclimation conditions, and individual fitness levels, thus making it difficult to establish a uniform relationship between exercise and CIVD across different datasets.

### Procedural factors: Altitude, normobaric, and hypobaric hypoxia

**Figure 10.**
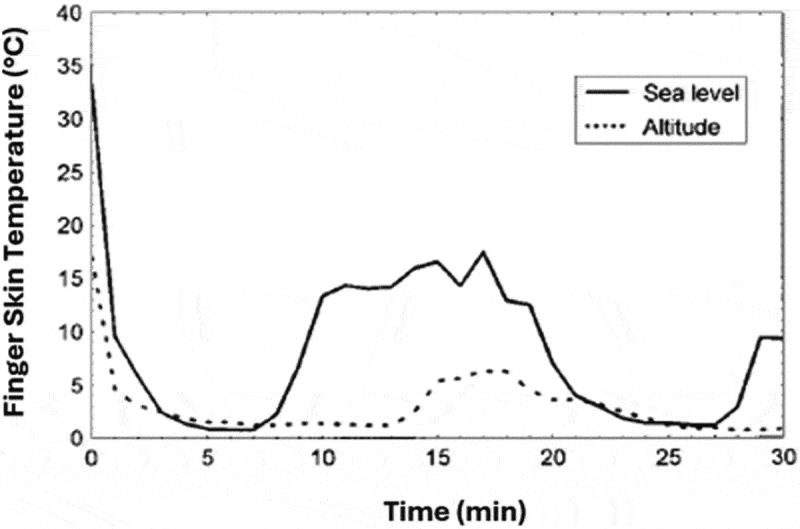
Typical example of CIVD response at sea level and altitude for a nonacclimatized participant displaying a decreased CIVD amplitude and delayed onset time at altitude. Figure reprinted with permission [34].

CIVD has been shown to be less pronounced at high altitude, suggesting an increased risk for cold injuries in high altitude cold environments [64]. Figure 10 demonstrates a typical example of finger skin temperature at sea level and at altitude, highlighting how altitude amplifies finger skin vasoconstriction from sympathetic overactivity [269] that can impair CIVD [34]. Additionally, at altitude, reduced oxygen tension in the inspired air and reduced air pressure on the body generally coexist with cold. In the study of Daanen and van Ruiten [34], however, the ambient temperature in the tents at altitude were not lower than the ambient temperature at sea level, leaving hypoxia and reduced external pressure on the body as mechanisms. Takeoka et al. [270] evaluated CIVD responses at Qinghai Plateau (cold + high altitude) and in a climactic chamber where a similar altitude was simulated (thermoneutral + high altitude) between low-altitude and high-altitude natives to separate the hypobaric hypoxia influence from the cold [270]. It was found that T_mean_ was lower at 4860 m compared to 2260 m at Qinghai peninsula, however in the climactic chamber, T_mean_ was higher at 4000 m compared to 2000 m, suggesting that the colder air temperatures, rather than hypobaric hypoxia, had the dominant effect on CIVD responses.

The physiological stress of high altitude is accurately simulated in hypobaric tanks, but not in hypoxic chambers, since in the latter environment the effect of air pressure on the body is not considered. Hypobaric hypoxia exhibits greater hypoxemia, hypocapnia, blood alkalosis, and a lower arterial oxygen saturation for the same ambient oxygen partial pressure due to greater breathing frequency, a lower tidal volume and minute ventilation compared to normobaric hypoxia [271]. Furthermore, a 2°C reduction in finger skin temperature was found when the atmospheric pressure was reduced from 1 to 0.5 atmosphere absolute [272]. The reduction in finger skin temperature was presumably mediated by an increase in sympathetic activity from the hypoxic and hypobaric environment [273]. Conversely, Meeuwsen et al. [274] found no CIVD differences between normobaric and hypobaric environments in healthy well-trained participants, however CIVD amplitude was reduced by 1°C compared to the control after acute hypoxic exposure for simulated altitudes of 3353 m (14% oxygen). In line with these observations, when comparing normobaric hypoxia set to the same level of 14% O_2_ [275], 17% O_2_ [262], and 13% O_2_ [262] to sea level (21%), T_mean_ and T_min_ did not differ during the cold-water immersion phase, however T_max_ of the fingers was lower in hypoxic conditions. O’Brien et al. [70] observed no differences between hypoxia levels in T_mean_ and T_min_, but onset time and T_max_ occurred earlier at 3000 m compared to sea level [70]. However, core temperature was not measured to control for the possible effect of increased core temperatures on onset time.

Breathing hypoxia impacts CIVD during cold stress and markedly impairs the rewarming responses of the hand following cold exposure [276]. T_mean_ and T_max_ have shown to be lower in hypoxia compared to sea level during the rewarming phase [275]. The lower finger skin temperature values are likely mediated by sympathetic overactivity when exposed to hypoxia [275]. Hypoxia can induce hyperventilation leading to reduced cutaneous perfusion in the fingers [275,276], and an increase in sympathetic outflow to peripheral blood vessels, resulting in vasoconstriction [277]. Additionally, when the brain does not get enough oxygen caused by hyperventilation, hypoxia can inhibit efferent impulses to the periphery [253].

CIVD has been shown to gradually improve during prolonged stay at altitude in the field [34,47], however intermittent laboratory hypoxic exposures reduced CIVD finger [48] and hand [278] skin temperatures during the posttest compared to the pretest. The CIVD reduction is likely because both hypoxic groups exhibited a lower baseline T_mean_ during the post-exposure condition compared to the pretest values. The lowered baseline T_mean_ can be explained by hypoxia-induced peripheral vasoconstriction [273,279], whereas the baseline T_mean_ was significantly elevated following the normoxic training. Additionally, the impaired CIVD response in both hypoxic groups could be attributed to hypoxic induced adaptations within the cutaneous microvasculature and decreased antioxidative capacity leading to suppression of the vascular endothelial function [280,281].

Keramidas et al. [48] controlled for ambient temperature and evaluated two short-term, intermittent hypoxic (12% inspired oxygen) training protocols (4 wk exercise training with 5 sessions/wk) where one group cycled in hypoxia and lived in normoxia (LL-TH), another cycled in normoxia preceded by a series of brief intermittent hypoxic exposures at rest (IHE+NOR), and the control cycled and lived in normoxia. During cold-water immersion, fingers were colder immediately after both hypoxic protocols (LL-TH: −1.2°C, IHE+NOR: −1.8°C, *p* = 0.01) compared to control. While living for 10 d in normobaric hypoxia (simulated altitude of 3400 m) and in the absence of cycling, Kounalakis et al. [282] found no significant differences in CIVD responses before and after the hypoxic confinement [282]. Additionally, Ruijs et al. did not observe effects of hypoxia on CIVD.

In summary, the CIVD response is generally diminished at high altitudes due to increased sympathetic activity, hypoxia, and reduced atmospheric pressure. Both normobaric and hypobaric hypoxia have been shown to lower finger skin temperatures, with acute hypoxic exposure leading to heightened vasoconstriction, though some studies report no significant changes. Adaptation to prolonged altitude exposure may improve CIVD, whereas intermittent hypoxic training appears to impair it. Notably, exercise may counteract some of hypoxia’s adverse effects through improved endothelial function and metabolic heat production, potentially enhancing CIVD [262] and rewarming responses [275]. Further research is warranted to clarify how different exercise types and frequencies mediate finger CIVD responses in hypoxia.

The effects of altitude on CIVD are complex, involving multiple interacting factors such as ambient temperature, oxygen availability, and atmospheric pressure. The heterogeneity in study designs, including variations in altitude levels, exposure durations, and environmental controls, made it challenging to isolate altitude as an independent moderator in the meta-analysis. Furthermore, differences in methodological approaches (e.g. field studies vs. simulated altitude chambers) contributed to inconsistencies in findings, leading to exclusion from the moderator analysis.

### Theoretical synthesis: The mechanisms of CIVD

#### Central mechanisms

A cold body core leads to an increase in sympathetic activity and a reduced CIVD magnitude, while a warm body core leads to an increased CIVD magnitude [30,32,261]. Thus, the magnitude of CIVD is dependent on sympathetic activity that is strongly mediated by central mechanisms, such as central body temperature. Similarly, the higher sympathetic activity at altitude compared to sea level [283] is linked to poorer CIVD magnitude [34,64,78], although these observations were not confirmed in a recent study [284]. Heat acclimation has been shown to reduce the activation threshold for skin sympathetic nerve activity, leading to an earlier activation of cutaneous vasodilatation [285]. In line with this observation, Ciuha et al. [29] observed a larger CIVD magnitude after heat acclimation, despite lower rectal temperatures. This highlights that the change in sympathetic activity has a larger impact on CIVD responses than the small drop in body core temperature, often observed with heat acclimation. In conclusion, increased sympathetic activity due to cold, altitude, and heat acclimation can lead to enhanced CIVD magnitudes, showing the importance of the sympathetic system in the CIVD response. Some authors believe that the sympathetic system may also be involved in the triggering of the CIVD response [286], therefore a closer look at the peripheral mechanisms is warranted.

#### Peripheral mechanisms

The pulsating blood flow observed during CIVD is likely linked to the presence of arterio-venous anastomoses (AVAs) in the skin where CIVD occurs [287]. 3D imaging studies and mathematical characterizations have revealed the structural complexity of AVAs, describing them as highly tortuous channels and glomus-like arrangements, particularly in the nail region and finger pad, which are key sites of thermoregulatory activity [288].

These specialized vascular structures, concentrated in regions prone to CIVD [289], such as the fingers, toes, and nail beds, possess thick muscular walls densely innervated by adrenergic axons, allowing dynamic regulation of blood flow in response to thermal stress [290,291]. AVAs are integral to the mechanism of CIVD due to their ability to facilitate rapid blood flow from arteries to veins, bypassing capillaries and warming peripheral tissues [2,290]. During cold exposure, AVAs typically remain closed below a local temperature of 21.5°C because of the heightened alpha-adrenoceptor activity [291]. However, prolonged contraction of the AVA smooth muscle becomes unsustainable at lower temperatures, leading to relaxation of the smooth muscles surrounding the AVA. The increased blood flow that is the result of this relaxation causes these vessels to reopen, characteristic of the “hunting” response observed during CIVD [290].

The rhythmic contraction and relaxation of AVAs are mediated by the sympathetic nervous system. This explains the strong CIVD response with a warm body core and the absence of CIVD during hypothermia. Active vasoconstriction is controlled by alpha-adrenergic stimulation, while passive vasodilation occurs due to reduced sympathetic activity or a nervous blockade at extremely low temperatures [42,290]. Cold-induced paralysis of AVA smooth muscle at tissue temperatures below 12°C further explains the periodic vasodilation observed during CIVD [287,292]. The ability of AVAs to alternate between vasoconstriction and vasodilation allows them to balance heat conservation with intermittent warming of peripheral tissues, highlighting their critical role in both thermoregulation and the unique cyclic nature of CIVD [288,291].

In a previous review on CIVD [2], four primary hypotheses were proposed to explain the underlying peripheral mechanisms:

1. Axon reflex hypothesis- An axon reflex or antidromic vasodilation is a reaction resulting in cutaneous vasodilation from noxious stimuli exciting receptive nerve endings of unmyelinated neurons that travel via the axon branches, thus releasing vasoactive substances at the excited sensory nerve endings [2,293]. This mechanism is considered unlikely since an experiment involving electrical stimulation of the middle fingers while immersed in water (5°C and 35°C) showed that no axon reflex could be evoked during CIVD [294].
2. Vasodilatory substance hypothesis – It was suggested that a dilating substance enters the bloodstream, with NO proposed as a potential candidate [295]. However, at the time, no experimental evidence was available to support this hypothesis. The role of NO has since been revisited considering emerging research and is further discussed below.
3. Adrenergic attenuation hypothesis – A decreased release of norepinephrine from adrenergic nerve endings, a concept further explored in subsequent work by Buijs et al. [296].
4. Direct cold effect on vascular smooth muscle hypothesis- The potential for cold to directly depress or even abolish vascular smooth muscle activity was also considered [297].

**Figure 11.**
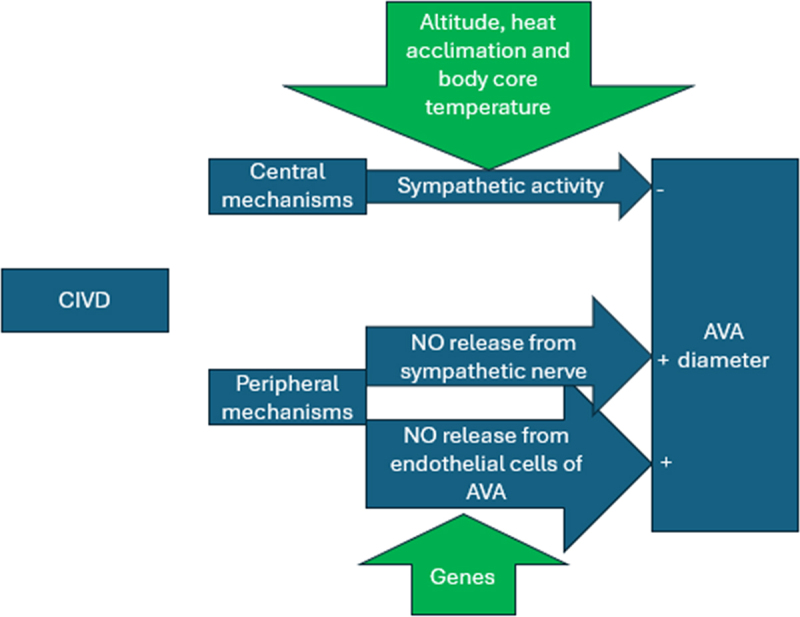
Hierarchical representation of the central and peripheral mechanisms involved in CIVD. The central mechanisms, including SNS activity, core temperature regulation, and altitude effects, influence CIVD magnitude and onset. The peripheral mechanisms, such as AVAs, vascular smooth muscle activity, and NO release, mediate the localized vasodilatory response. The interaction between these mechanisms determines the characteristic cyclic nature of CIVD. Green = modifying factors.

Since 2003, additional research has refined these perspectives, thus reducing the previous four hypotheses to two main proposed mechanisms for CIVD: 1) sympathetic nervous system failure and 2) emerging vasodilators from the vascular wall and the sympathetic nerve ending. Figure 11 displays a hierarchical diagram outlining the central and peripheral mechanisms of CIVD.

#### Proposed mechanism 1: Sympathetic nervous system

**Figure 12.**
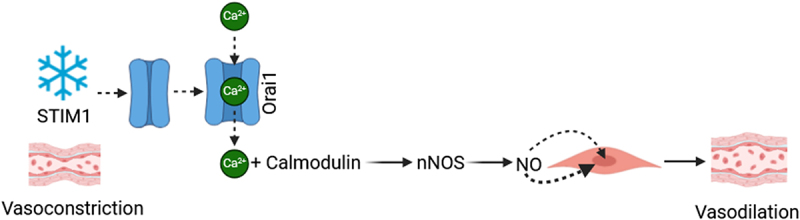
Schematic representation of proposed mechanism 1. The sympathetic nervous system nerve acts on the AVA smooth muscle, where cold exposure initiates a signaling cascade. STIM1 detects cold and activates ORAI1 channels, allowing Ca^2 +^ influx into the cell. The increased intracellular Ca^2 +^ binds to calmodulin, which activates neuronal nitric oxide synthase (nNOS). nNOS subsequently produces nitric oxide (NO), which freely diffuses into the surrounding vascular smooth muscle tissue, inducing vasodilation.

As discussed above, the sympathetic nervous system (SNS) plays a significant role in CIVD, but does it elicit the CIVD response directly? SNS nerves can be activated by cold stimuli. For example, mouse superior cervical ganglion neurons, which innervate areas rich in AVAs such as the ear, can be directly activated by cooling below 16°C [298]. This intrinsic cold sensitivity was suggested to play a role in cold defense mechanisms. This is further supported by the discovery of a cold-induced calcium entry pathway in these neurons, involving the STIM1-ORAI1 ion channel complex, which allows calcium influx into sympathetic neurons at noxious cold temperatures (≤10°C) and could lead to the activation of neuronal nitric oxide synthase (nNOS) and subsequent nitric oxide (NO) release [296,299]. As illustrated in Figure 12, NO-mediated vasodilation (CIVD) may serve as a protective response to prevent frostbite in peripheral tissues, particularly when nerve conduction is inhibited at extreme cold temperatures [300].

Moreover, the SNS exhibits dual functionality, with cholinergic and noradrenergic phenotypes allowing for alternating vasodilation and vasoconstriction at AVAs [301]. These neurons alternate between releasing acetylcholine or NO to induce vasodilation and noradrenaline to promote vasoconstriction, facilitating the cyclic nature of CIVD. Localized SNS activation, such as that driven by the STIM1-ORAI1 mechanism, predominates under extreme cold conditions where CNS-mediated vasoconstriction is not required [296]. These findings underscore the SNS’s complex and context-dependent role in CIVD, balancing thermoregulatory demands with the need to preserve tissue integrity.

#### Proposed mechanism 2: Vasodilators (nitric oxide)

The vasodilator nitric oxide is likely to be a key mediator in CIVD, primarily due to its vasodilatory properties and ability to modulate sympathetic vasoconstriction [302]. NO is continuously released from the nerve endings innervating the smooth muscle of AVAs to maintain basal vascular tone, with production increasing in response to various stimuli, including shear stress and cold exposure [303]. Mechanistically, NO can inhibit sympathetically mediated vasoconstriction by altering adrenergic neurotransmission and reducing vascular responsiveness to adrenergic agents [302]. Durand et al. [302] showed that NO attenuates vasoconstriction using microdialysis experiments with sodium nitroprusside (SNP) during cold stress independently of nonspecific increases in cutaneous vascular conductance (CVC). This suggests that NO serves as a regulatory counterbalance to sympathetic activity during cold stress.

Despite the theoretical potential of NO to enhance CIVD, recent studies examining nitrate supplementation to elevate NO levels have yielded mixed results [304]. Beetroot juice supplementation has been shown to augment skin blood flow and reduce systemic blood pressure [305]. However, studies by Wickham et al. [103] and Wakabayashi et al. [99] found no significant impact of beet root juice on CIVD parameters during cold exposure, though Wakabayashi et al. reported enhanced finger blood flow during rewarming, suggesting a vasodilatory effect overwhelmed by cold-induced vasoconstriction [99]. Similarly, Alba et al. [306] found that providing 900 mg of cocoa flavanol supplementation over 8 d, another method to elevate NO levels, did not significantly influence CIVD despite high dosage levels. Additionally, research on individuals with cold sensitivity or Raynaud’s phenomenon, who exhibit impaired vascular responses, has also shown limited efficacy of beet root juice in improving extremity blood flow or temperature during cold exposure [220,307]. These findings suggest that while NO can modulate vascular tone and attenuate sympathetic vasoconstriction, its role in CIVD is complex and potentially limited under extreme cold conditions. Further studies are needed to clarify the conditions under which NO contributes to CIVD and to explore its interaction with other vasodilatory mechanisms, such as endothelium-derived hyperpolarizing factors (EDHF). A future study may consider directly measuring NO concentrations in both the blood vessel and surrounding tissue during cold exposure to determine its localized contribution to CIVD and to assess whether inadequate NO bioavailability limits vasodilation, which may help explain the limited effectiveness of beetroot juice supplementation.

The role of NO in vasodilation during CIVD is recently supported by genetic research. Yasukochi et al. [104] investigated genetic and physiological mechanisms underlying CIVD through wavelet analysis of skin blood flow and genome-wide association studies (saliva samples) during finger cold-water immersion (5°C) in a Japanese cohort (*n* = 94) [104]. They identified that individuals without a CIVD response exhibited blunted endothelial nitric oxide (eNO)-independent and neurogenic activity, potentially linked to genetic variants in the COL4A2 and PRLR genes. COL4A2 encodes the alpha-2 chain of type IV collagen [308] and “might affect vascular dynamics during finger cold-water immersion through changes in the affinity for COL4A1 in heterotrimer formation” [104]. PRLR molecules bind prolactin, and the interaction has shown to affect regulation of NO-independent vasodilation in rats [309].

Findings suggest that CIVD is influenced by mechanisms such as endothelium-derived hyperpolarizing factor (EDHF)-mediated vasodilation in small vessels [310,311] and neurogenic factors, rather than eNO-dependent activity. Genes like COL4A2 and PRLR may contribute to vascular smooth muscle relaxation via EDHF [312,313], indicating their potential involvement in CIVD-related pathways [311]. Furthermore, epigenetic modifications could play a role in shaping phenotypic responses to cold exposure, adding another layer of complexity to CIVD variability [104]. The study also highlights the genetic basis for interindividual and interethnic differences in CIVD, emphasizing the importance of further research to explore these pathways.

In summary, the four previously proposed hypotheses for CIVD have been refined into two primary mechanisms: (1) sympathetic nervous system modulation and (2) vasodilators. Future research is needed to determine which mechanism has the greatest impact on CIVD magnitude and how they interact under different conditions.

### General discussion

This meta-analysis and literature review synthesized quantitative and qualitative data across studies investigating CIVD, revealing consistent patterns in temperature responses and onset times, and highlighting key moderating variables such as sex, part immersed, and body morphology. Our evidence synthesis demonstrated that while T_min_, T_max_, and T_mean_ are tightly correlated and reflective of CIVD magnitude, onset time remains independent, supporting the theoretical distinction between CIVD initiation and amplitude. Theoretical synthesis of the findings supports a dual-control model in which local cooling triggers CIVD onset, while sympathetic nervous system activity regulates its magnitude.

Correlation analyses revealed that T_min_, T_max_, and T_mean_ were highly interrelated, with correlation coefficients ranging from 0.65 to 0.98 (Table 4). These temperature metrics reflect the magnitude of the CIVD response and appear much less related to its onset (*r* = −0.21 to −0.27). A longstanding debate exists in the literature regarding the mechanisms underlying CIVD initiation [286,314,315]. While Flouris and Cheung proposed that sympathetic withdrawal triggers CIVD, Daanen argued that local vasodilation in the extremities initiates the response. Given the significant role of AVAs in CIVD [287] and the observed relative independence of onset time from CIVD magnitude in our analysis, it may be reasonable to suggest that local finger/toe temperatures trigger the response, while CIVD magnitude is modulated by sympathetic control and core body temperature. This aligns with Sendowski et al. [83], who observed that “the onset of CIVD could be influenced by local cooling, independently of the general sympathetic stimulation.” Thus, CIVD likely serves not only to protect against cold injury but also to facilitate heat dissipation, as argued by Flouris and Cheung.

Forest plots and outlier analyses identified extreme values for some dependent variables across studies. However, most outliers could be plausibly explained. For example, Maley et al. [60] demonstrated delayed CIVD onset among individuals of African descent, while others [36,38] employed electrical stimulation during immersion, potentially delaying the CIVD response. Elevated T_max_ and T_min_ values were often associated with immersion in relatively warm water (e.g. 15°C [66]) or exposure to a warm ambient environment [84]. The highest T_mean_ (17.8°C) was recorded by Sendowski et al. [83] during the immersion of a single index finger in 5°C water. Once the entire hand or the contralateral hand was immersed, sympathetic activation increased, and T_mean_ values subsequently decreased.

Moderator analyses further revealed that onset time was shorter when only the finger was immersed, when participants were female, among cold-indigenous individuals, with increasing BSA, and with decreasing age. If CIVD is initiated by low local tissue temperatures, these findings are consistent with the notion that females, who typically have smaller fingers, cool more rapidly and thus show earlier onset. The faster onset for finger-only immersion compared to whole-hand immersion remains difficult to explain, as similar cooling rates would be expected. Age-related decline in vasoconstrictive capacity [316] likely contributes to faster finger cooling and earlier onset in older adults. Cold-indigenous populations may initiate CIVD at higher local temperatures, offering additional protection against cold. This phenomenon is especially evident in the Tuva population, who have inhabited cold environments for over 70,000 years and exhibited earlier onset times compared to matched Dutch controls [81]. Individuals with a higher surface area-to-mass ratio are expected to cool more quickly, which was supported by our meta-analysis data (Figure 3). While lower water temperatures typically accelerate tissue cooling and would be expected to shorten onset time, our meta-analysis findings did not support this. At 15°C, CIVD did not consistently occur [66], likely because tissue temperature thresholds were not reached. The meta-analysis also showed that prewarming the hand did not significantly affect the onset time.

Future studies should refine CIVD modeling by exploring the threshold local tissue temperatures required for onset across varying immersion sites and conditions, and by integrating real-time sympathetic nerve activity measurements. Additionally, longitudinal studies could examine how repeated cold exposure alters CIVD responses and whether training or acclimatization modifies onset thresholds or amplitudes. More diverse population samples, including various age groups, ethnic backgrounds, and fitness levels, are also essential for a comprehensive understanding of the physiological and adaptive significance of CIVD across human populations.

## Supplementary Material

Appendix figures_Final.docx

## Data Availability

The data and R code used for the meta-analysis are available upon request.

## Abbreviations

#P no doubles: the number of studies in which the population was only measured once
#P within: the number of studies in which the study population was used for more than one measurement
A: the number of articles
Age*SD*: standard deviation of age in years
AVAs: arterio-venous anastomoses
BMI: body mass index
BMI: body mass index
BSA: body surface area
BSA: body surface area
CI: confidence interval
CIVD: cold-induced vasodilation
ColdInd: cold indigenous
CVC: cutaneous vascular conductance
*df*: degrees of freedom
EDHF: endothelium-derived hyperpolarizing factors
eNO: endothelial nitric oxide
Fingerimm: immersed finger
Fingermeas: measured finger
H: hand
*I^2^b*: the heterogeneity variance between studies
*I^2^tot*: the total heterogeneity variance
*I^2^w*: the heterogeneity variance for within studies
IF: index finger
IHE+NOR: cycled in normoxia preceded by a series of brief intermittent hypoxic exposures at rest
ISO: International Organization for Standardization
Kg: kilogram
LB: lower bound
LF: little finger
LL-TH: cycled in hypoxia and lived in normoxia
*M*: multiple effect sizes for which mean and standard deviation were available
*M_+_*: meta-analytical mean
MF: middle finger
Min: minute
nNOS: nitric oxide synthase
NO: nitric oxide
NO_2_^−^: nitrogen dioxide
NO_3_^−^: nitrate ion
NSs: not stated
P: study populations
Part_immersed_: part immersed
PI: prediction interval
PrewarmD: duration of prewarming
PrewarmT: temperature of water used during prewarming
PropMF: proportion male in the dataset
*Q*: heterogeneity that was significant for all variables at p = 0.001
RH: relative humidity
S: single
SA:V: surface area to volume ratio
*SD*: standard deviations
*SE*: standard error of the mean
SM: surface to mass ratio
SNP: sodium nitroprusside
Stature*SD*: standard deviation of the stature in cm
STIM1-ORAI1-: Stromal Interaction Molecule 1- Calcium Release-Activated Calcium Modulator 1
T_amb_: ambient temperature
T_max_: maximum finger skin temperature of the first wave,
T_mean_: mean finger skin temperature
T_min_: minimum finger skin temperature
TRPM8 channel: Transient Receptor Potential Melastatin 8
UB: upper bound
WaterT: water temperature
Weight*SD*: standard deviation of body
σ^2^b: between-studies variance
σ^2^w: variance within studies
