# Supplementary material for: Cold-induced vasodilation: A meta-analysis
Source: Temperature (Austin). 2026 Apr 14;13(2):139–85. doi: 10.1080/23328940.2026.2646391 (PMC13251525; doi:10.1080/23328940.2026.2646391)
Supplement: Appendix figures_Final.docx [file KTMP_A_2646391_SM3825.docx]

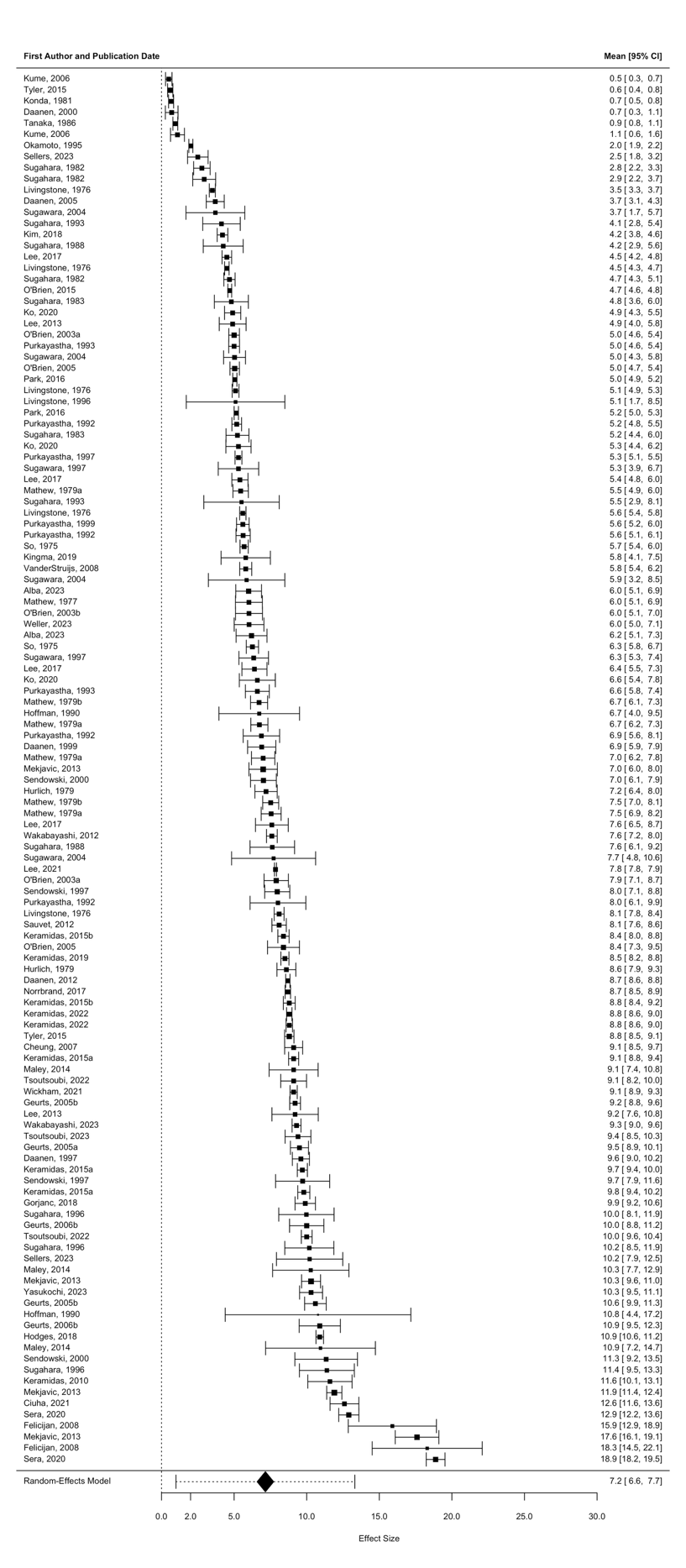


**Supplemental Figure 1:** Forest Plot - T_min_ (°C)


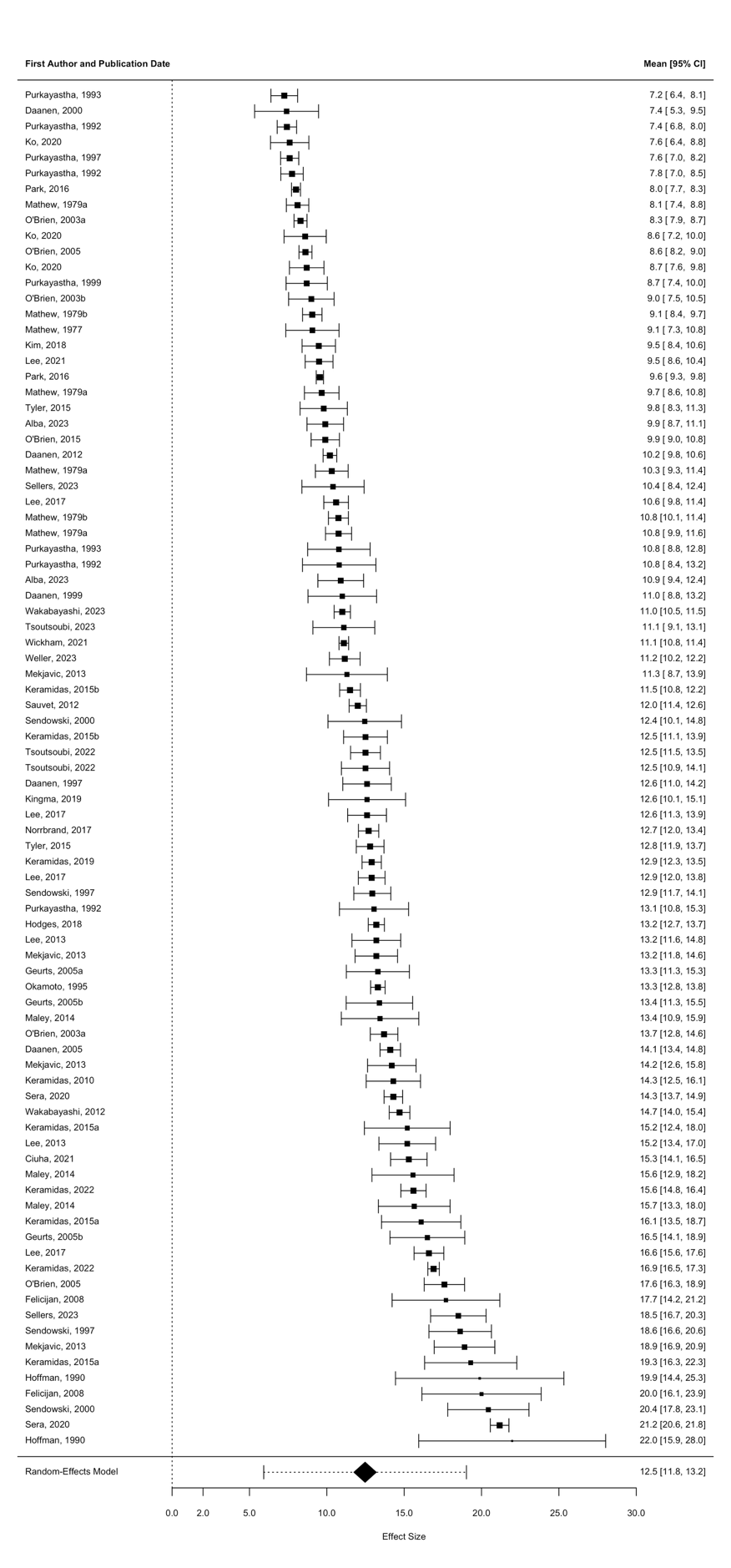


**Supplemental Figure 2:** Forest Plot - T_max_ (°C)

**
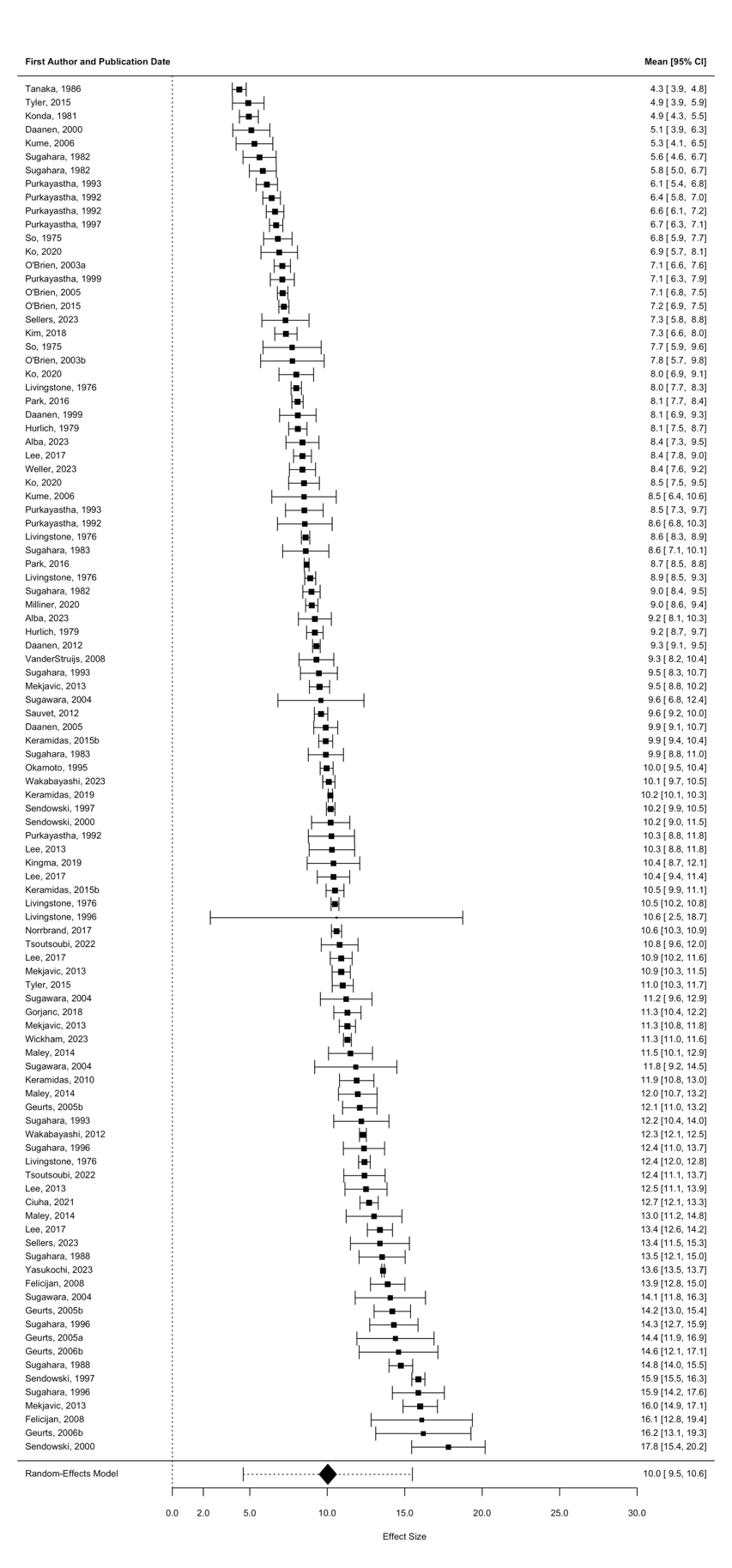
**

**Supplemental Figure 3:** Forest Plot - T_mean_ (°C)

**
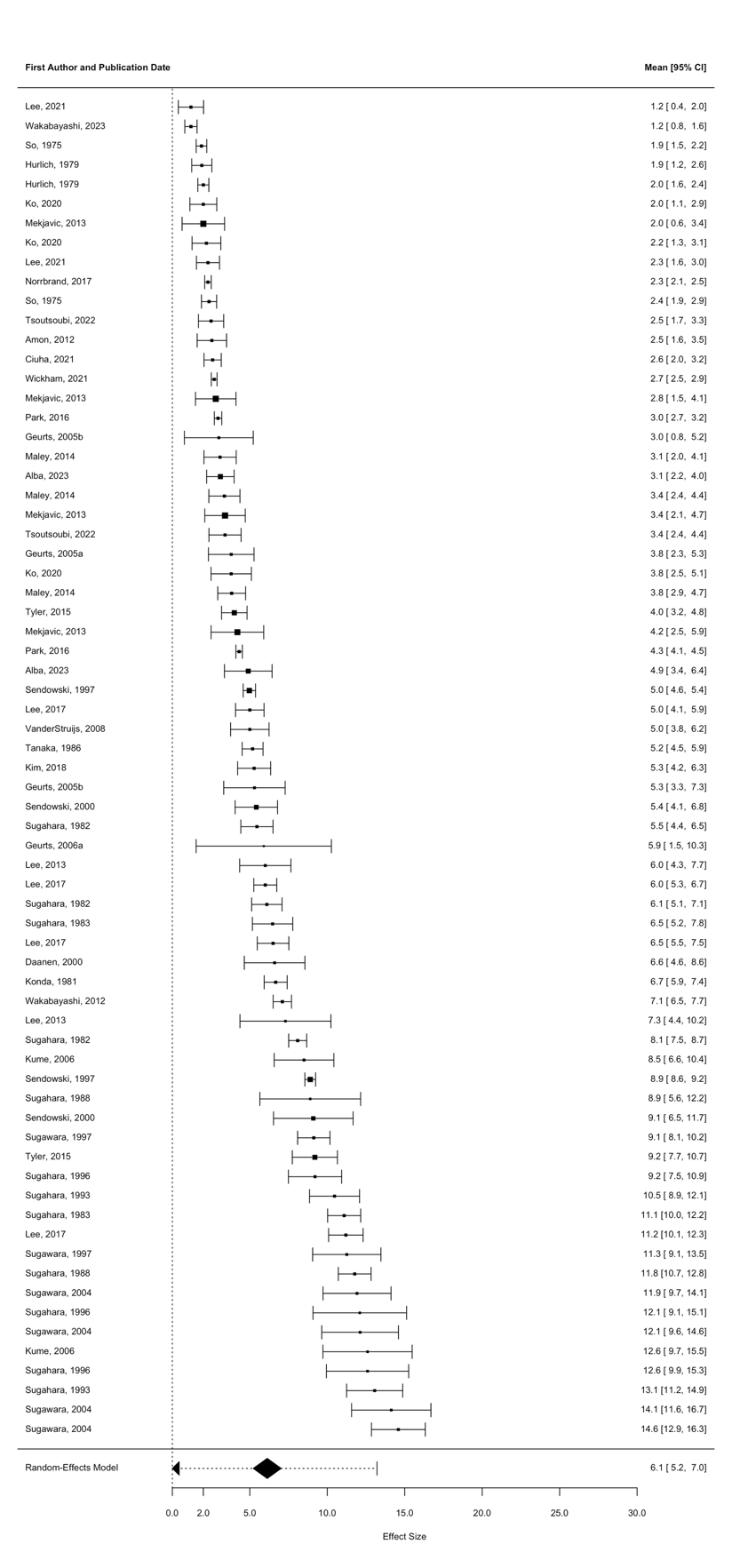
**

**Supplemental Figure 4:** Forest Plot - Amplitude (°C)


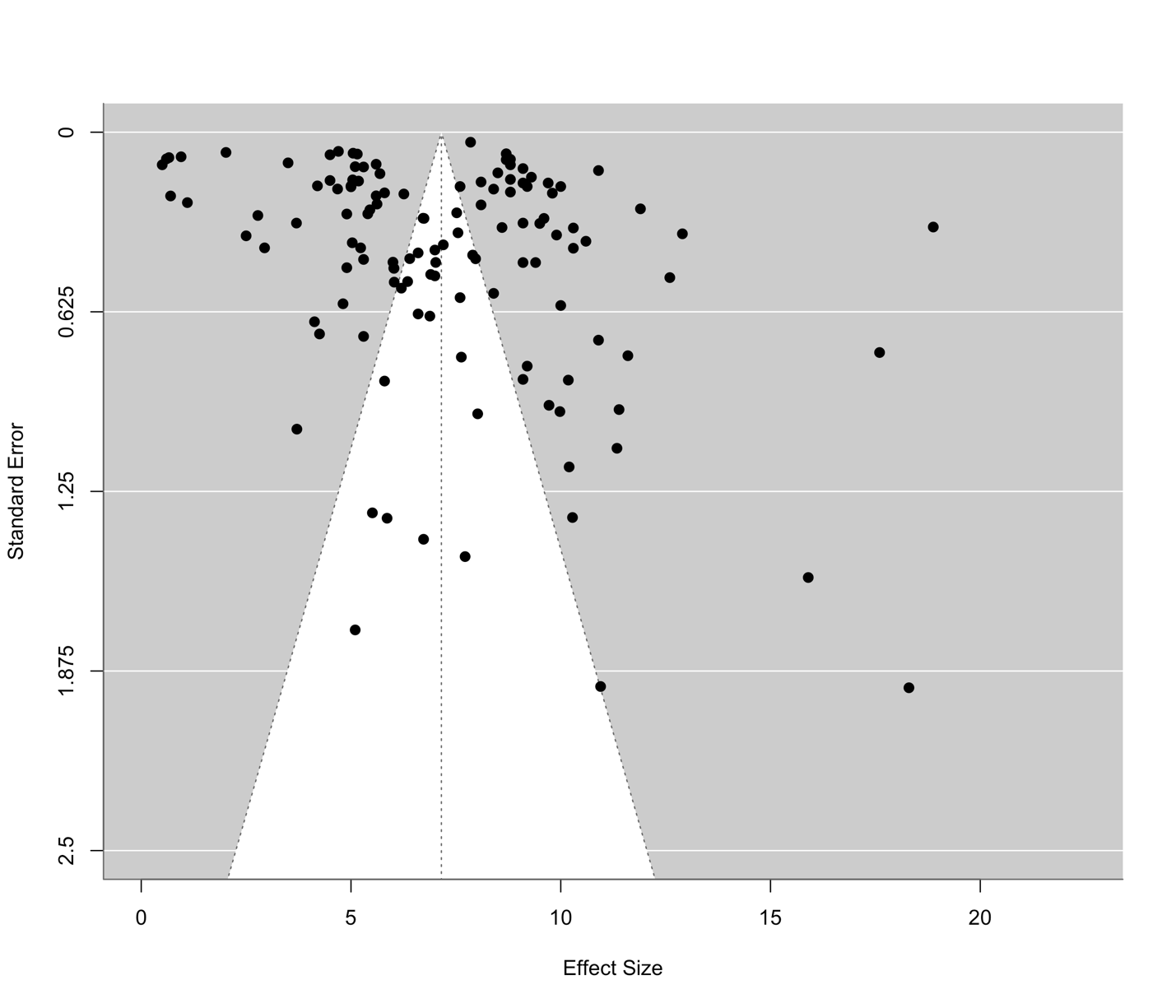


**Supplemental Figure 5:** Funnel Plot - T_min_ (°C)


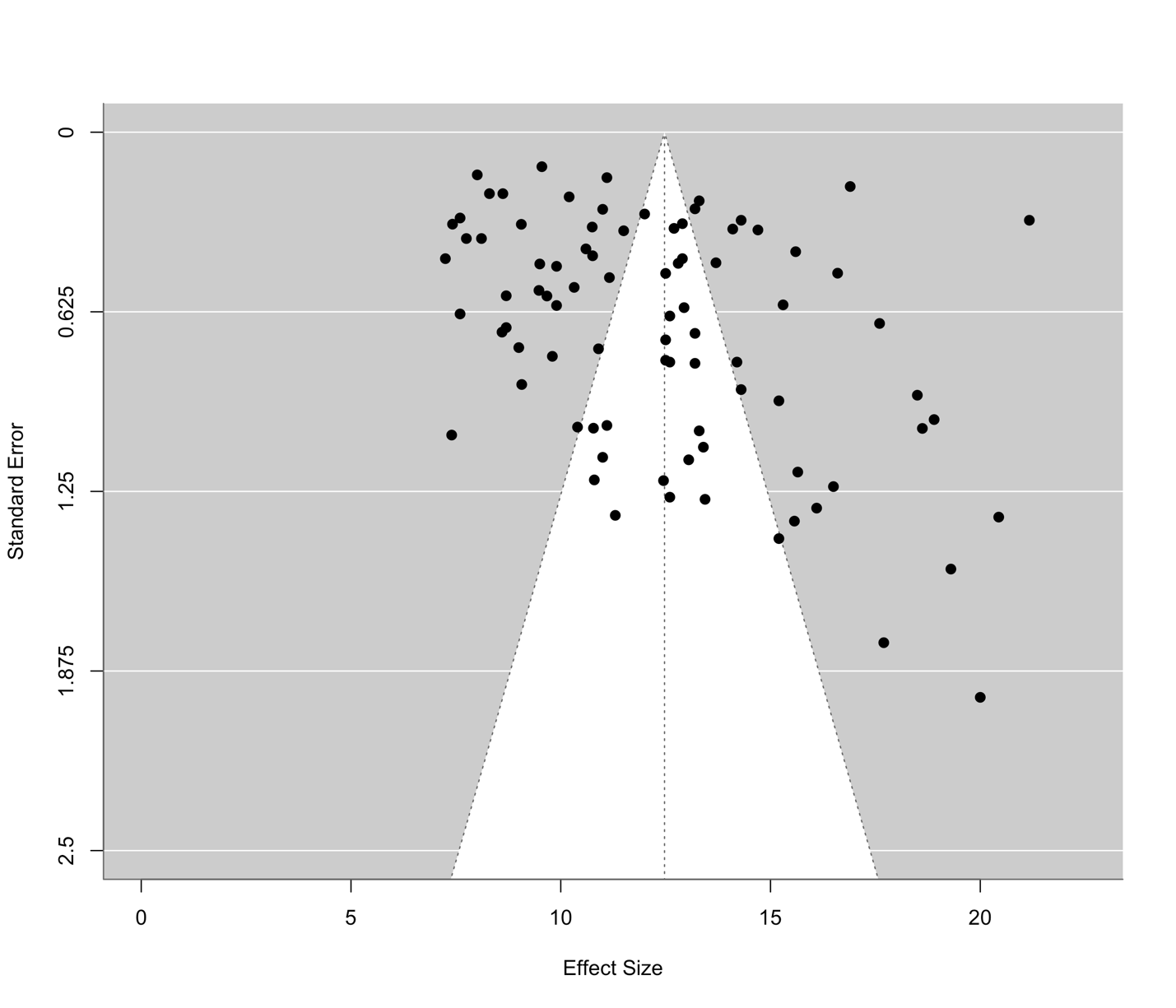


**Supplemental Figure 6:** Funnel Plot - T_max_ (°C)


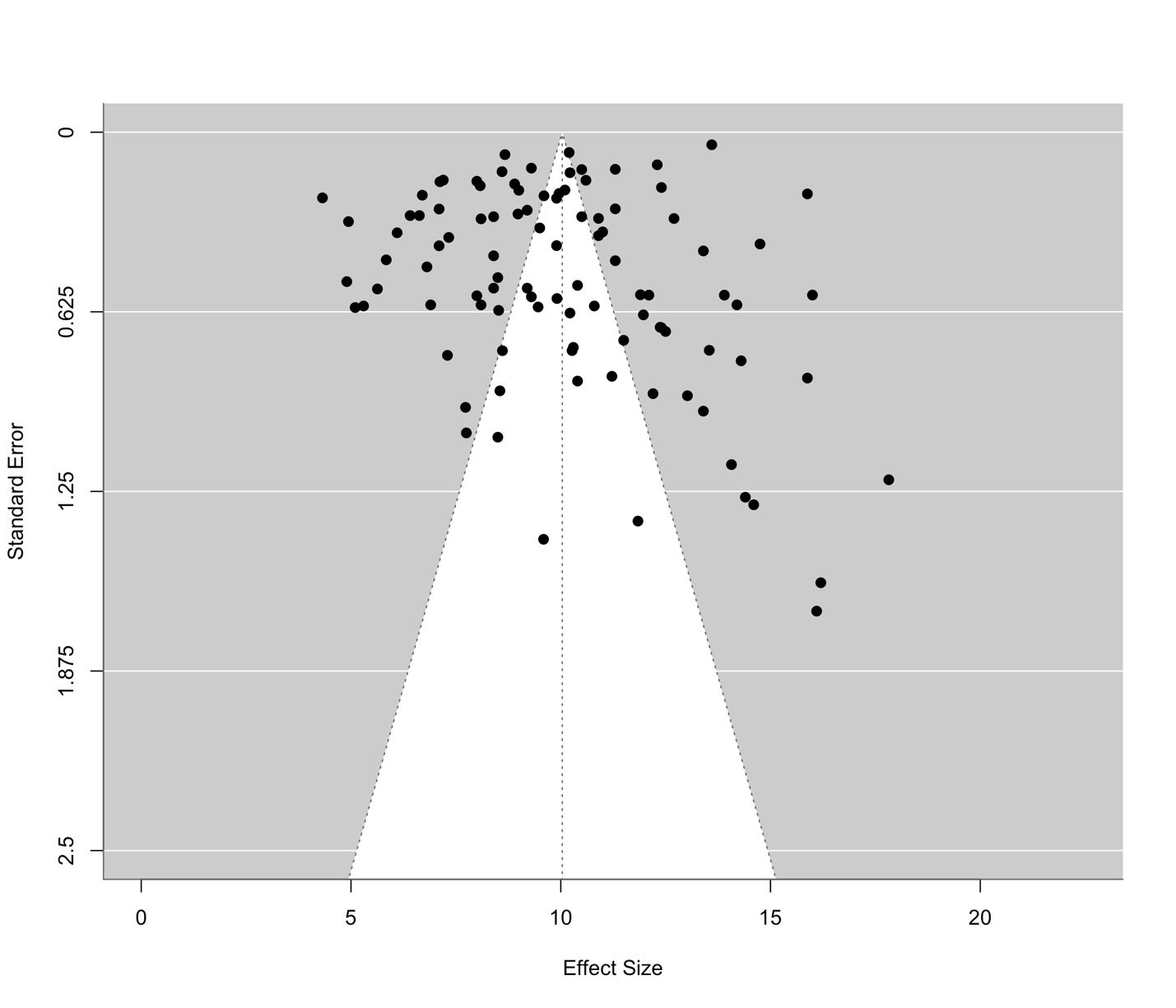


**Supplemental Figure 7:** Funnel Plot - T_mean_ (°C)


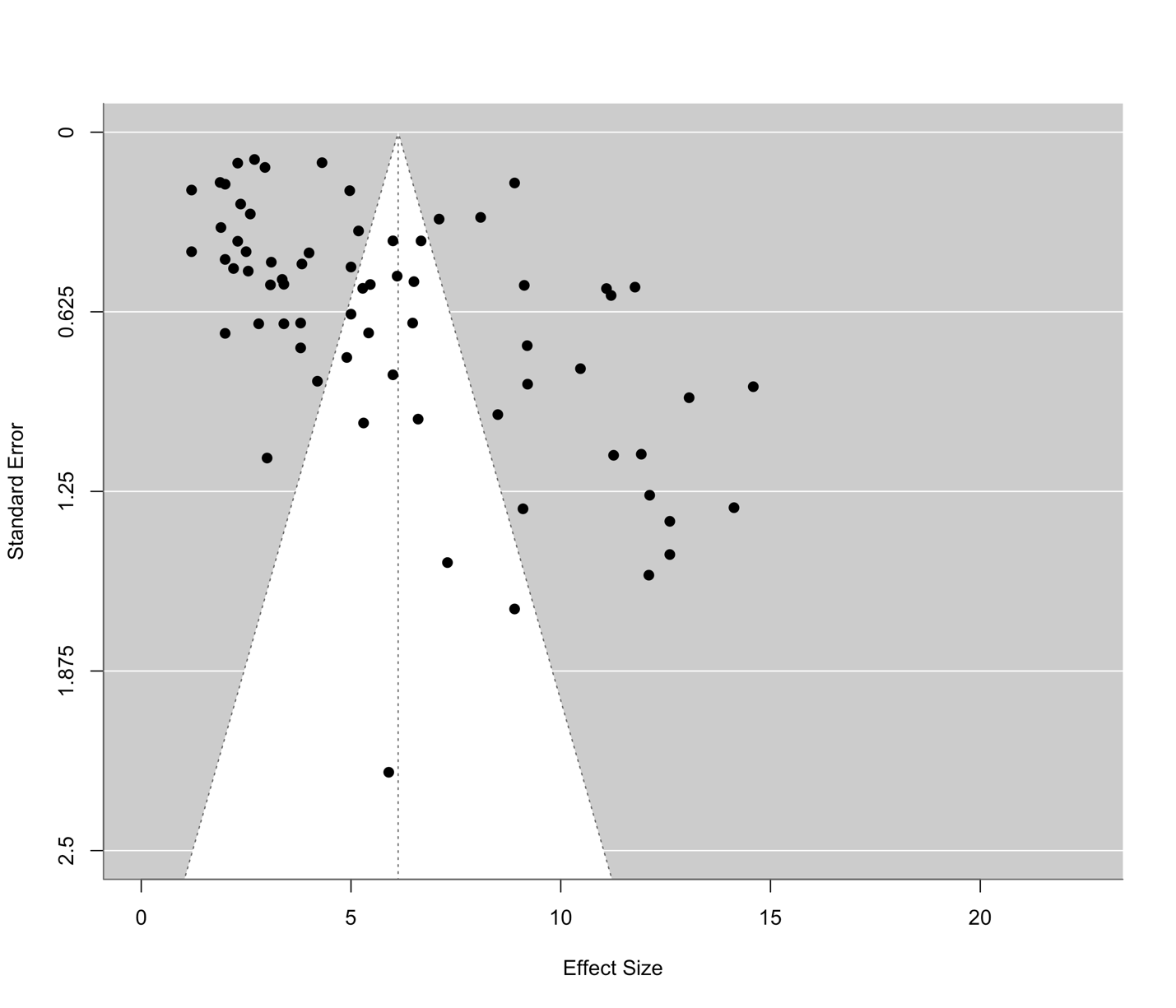


**Supplemental Figure 8:** Funnel Plot - Amplitude (°C)


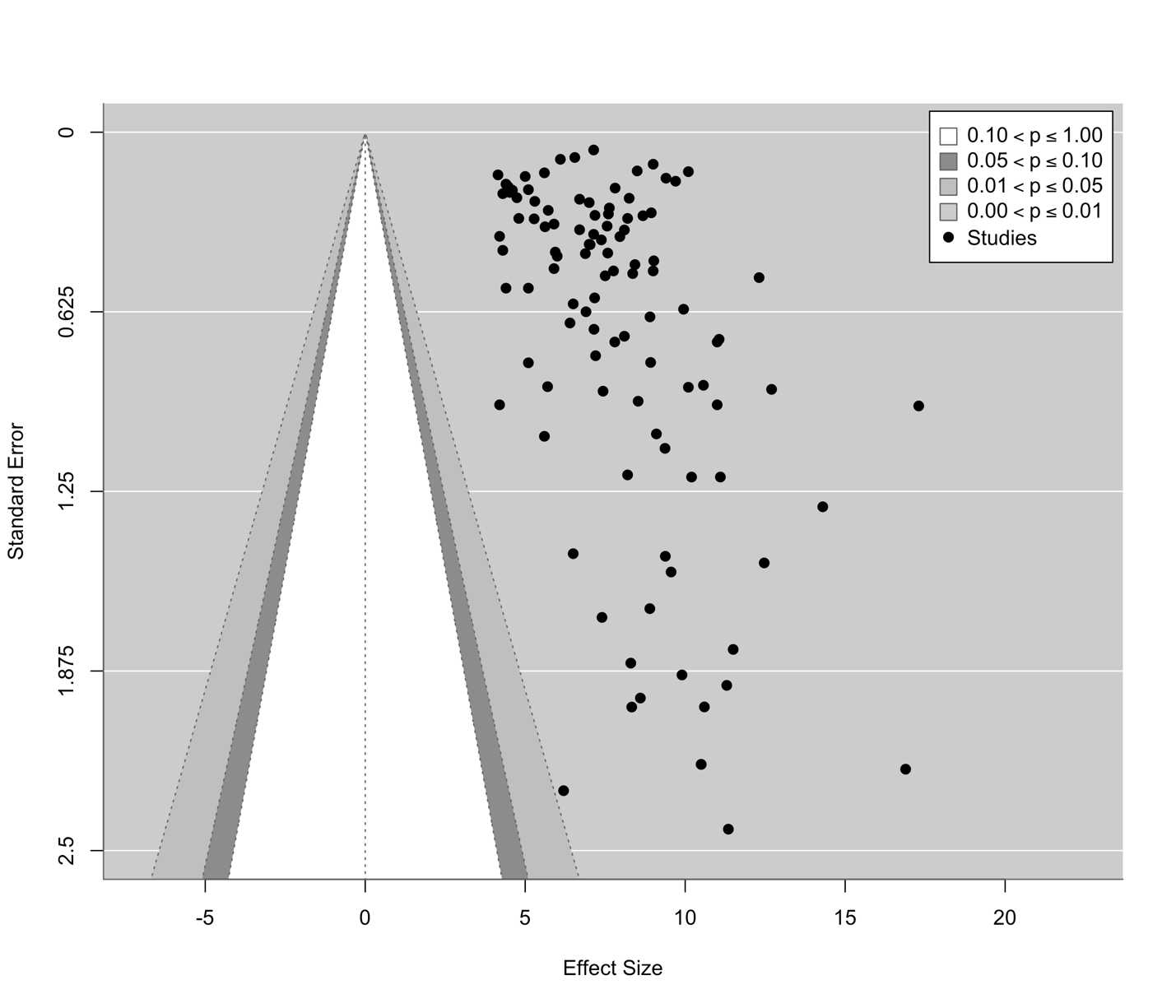


**Supplemental Figure 9:** Contour-Enhanced Funnel Plot - Onset (min)


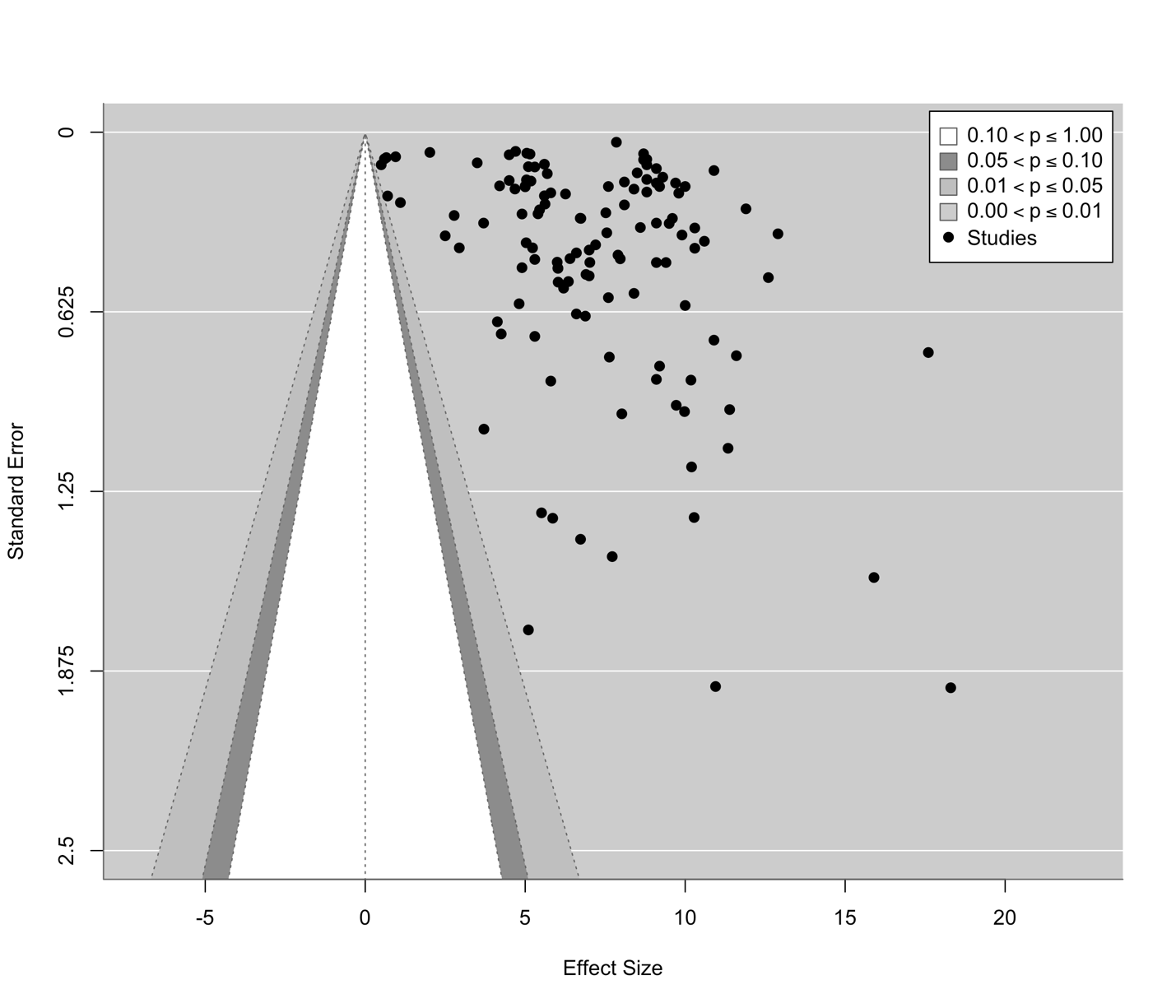


**Supplemental Figure 10:** Countour-Enhanced Funnel Plot - T_min_ (°C)


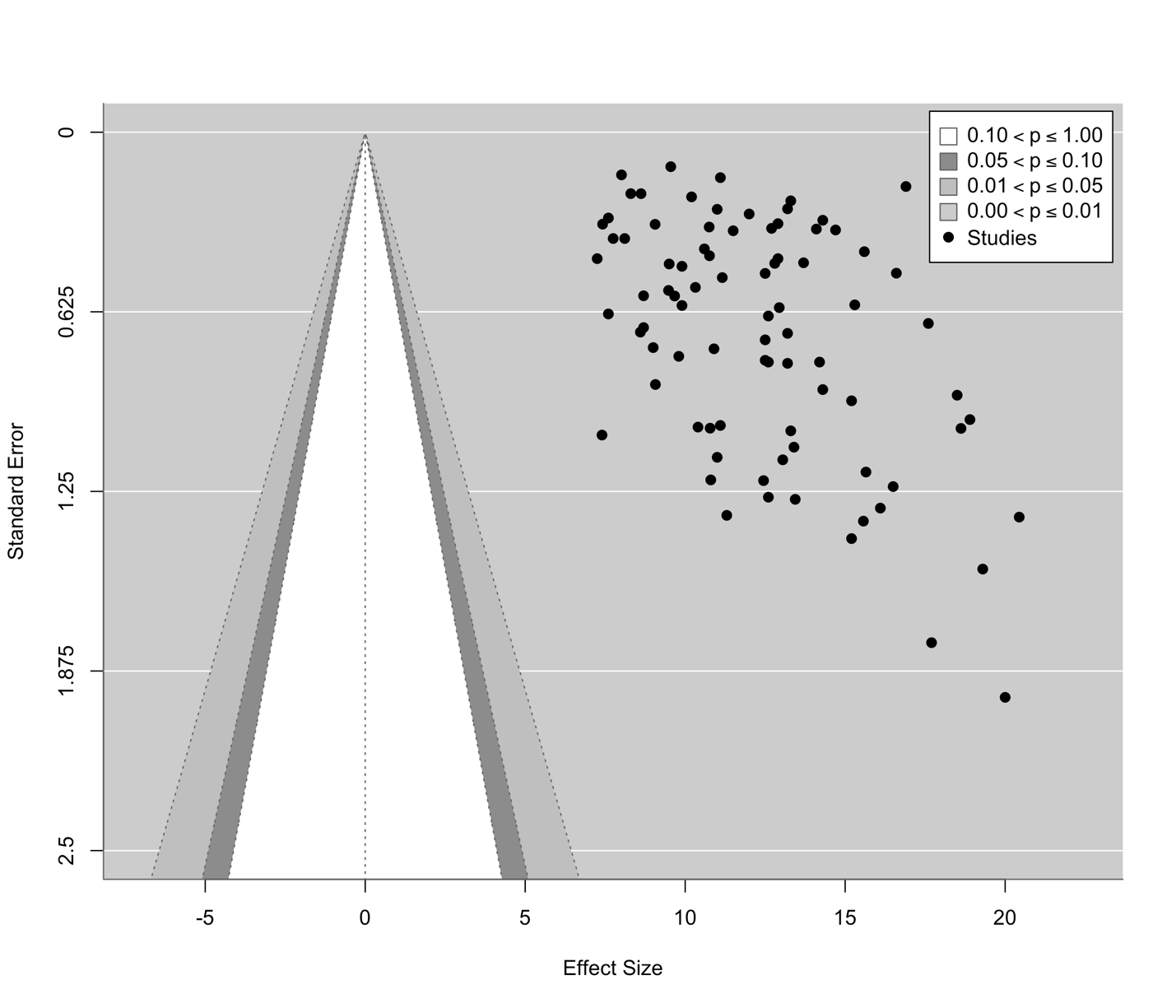


**Supplemental Figure 11:** Countour-Enhanced Funnel plot - T_max_ (°C)


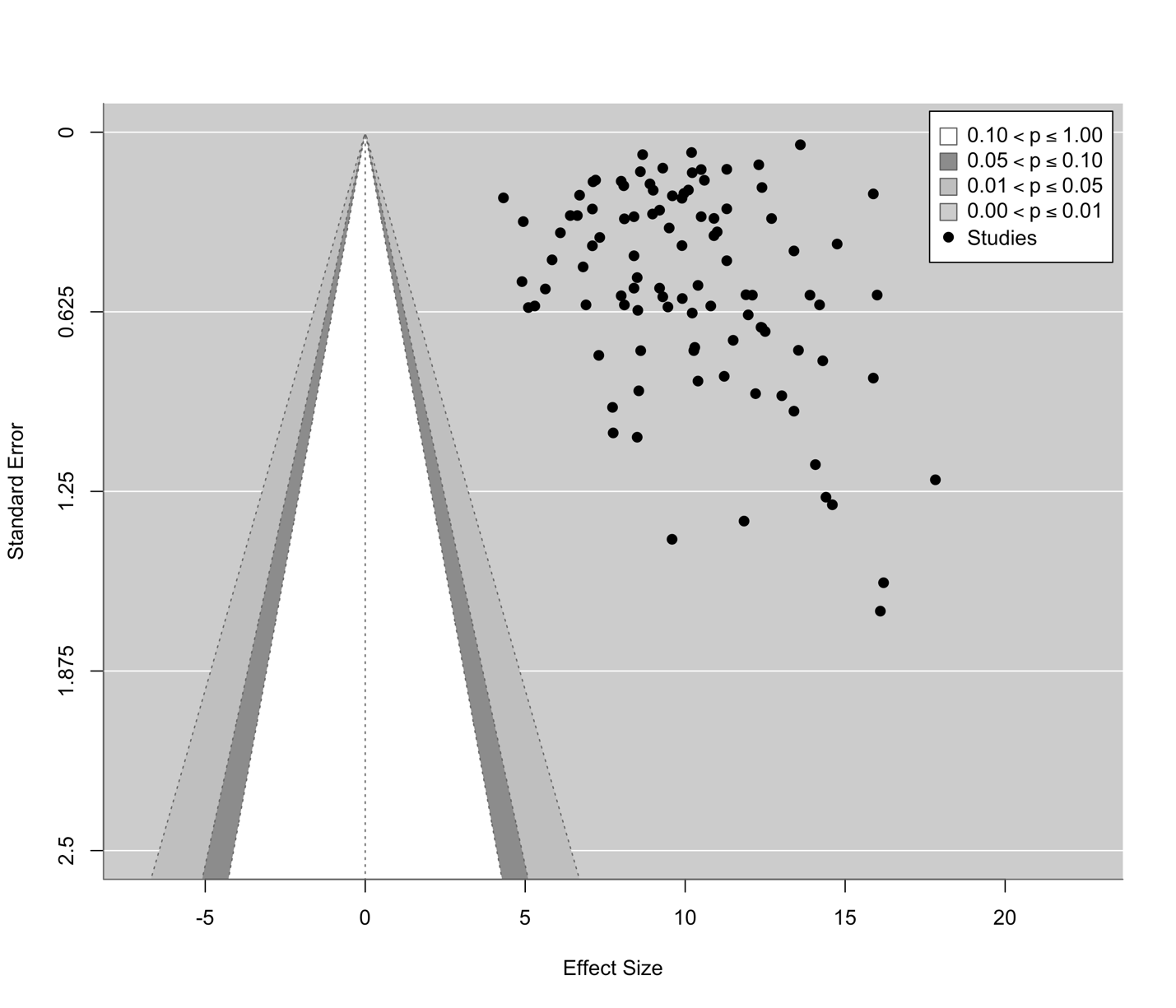


**Supplemental Figure 12:** Contour-Enhanced Funnel Plot - T_mean_ (°C)


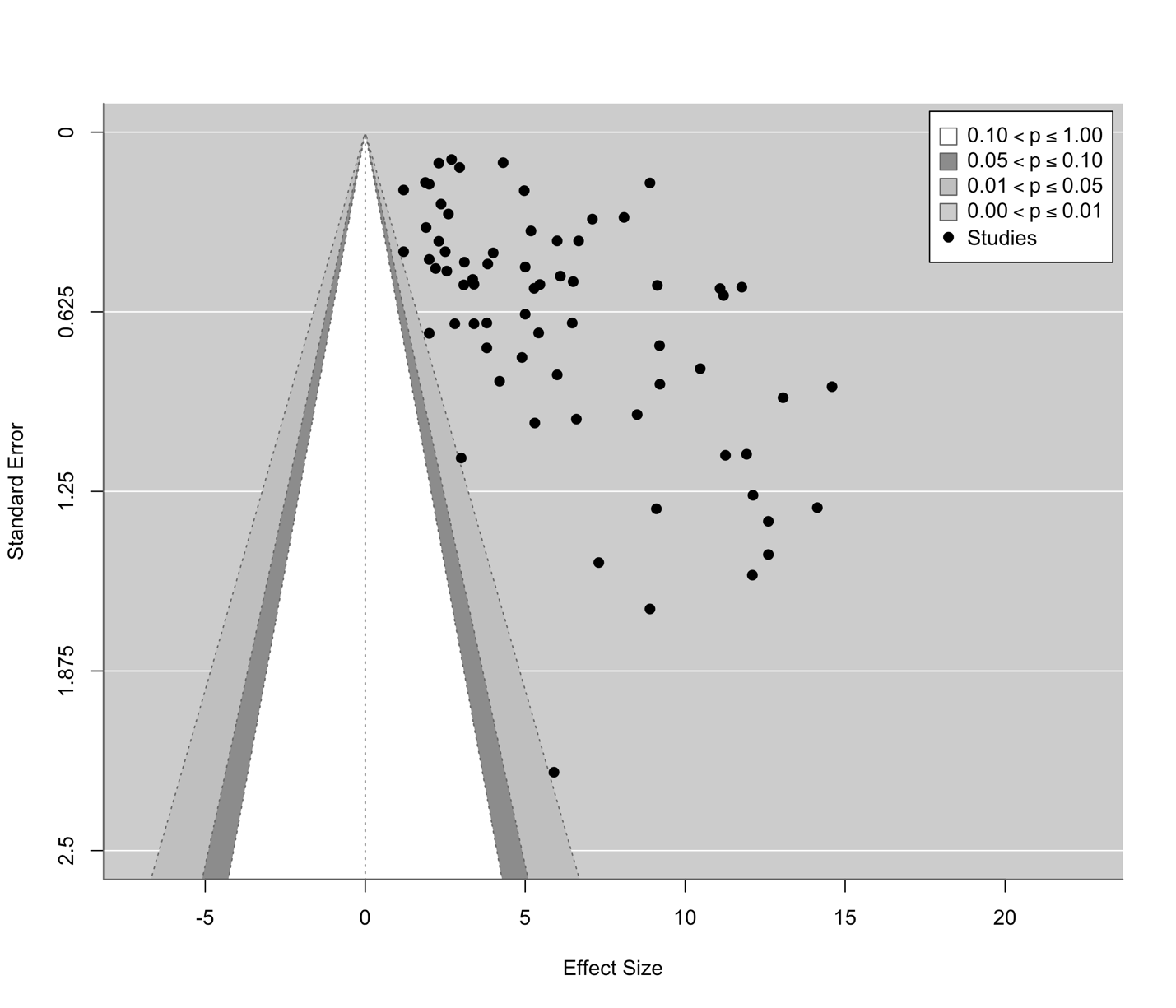
**Supplemental Figure 13:** Contour-Enhanced Funnel Plot – Amplitude (°C)


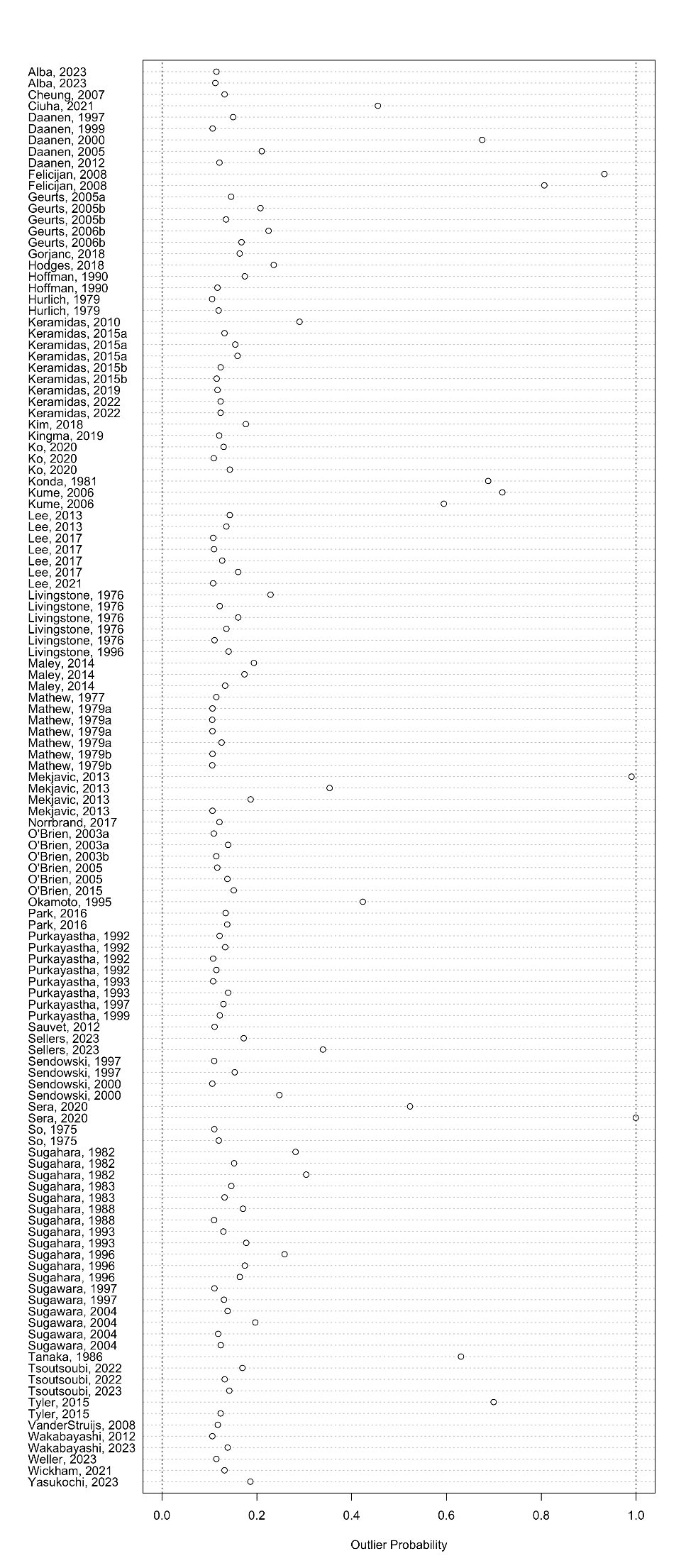


**Supplemental Figure 14:** Probability of Outliers - T_min_ (°C)


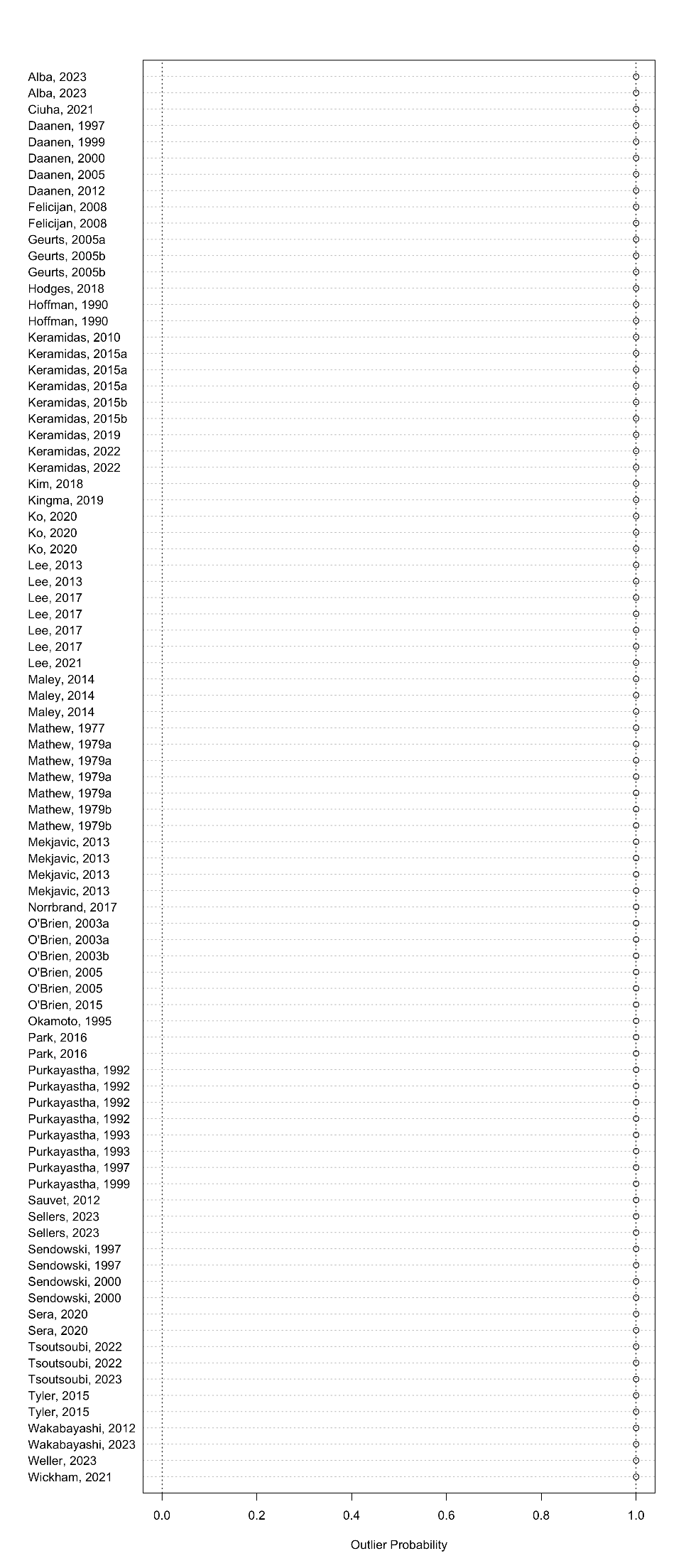


**Supplemental Figure 15:** Probabulity of Outliers - T_max_ (°C)


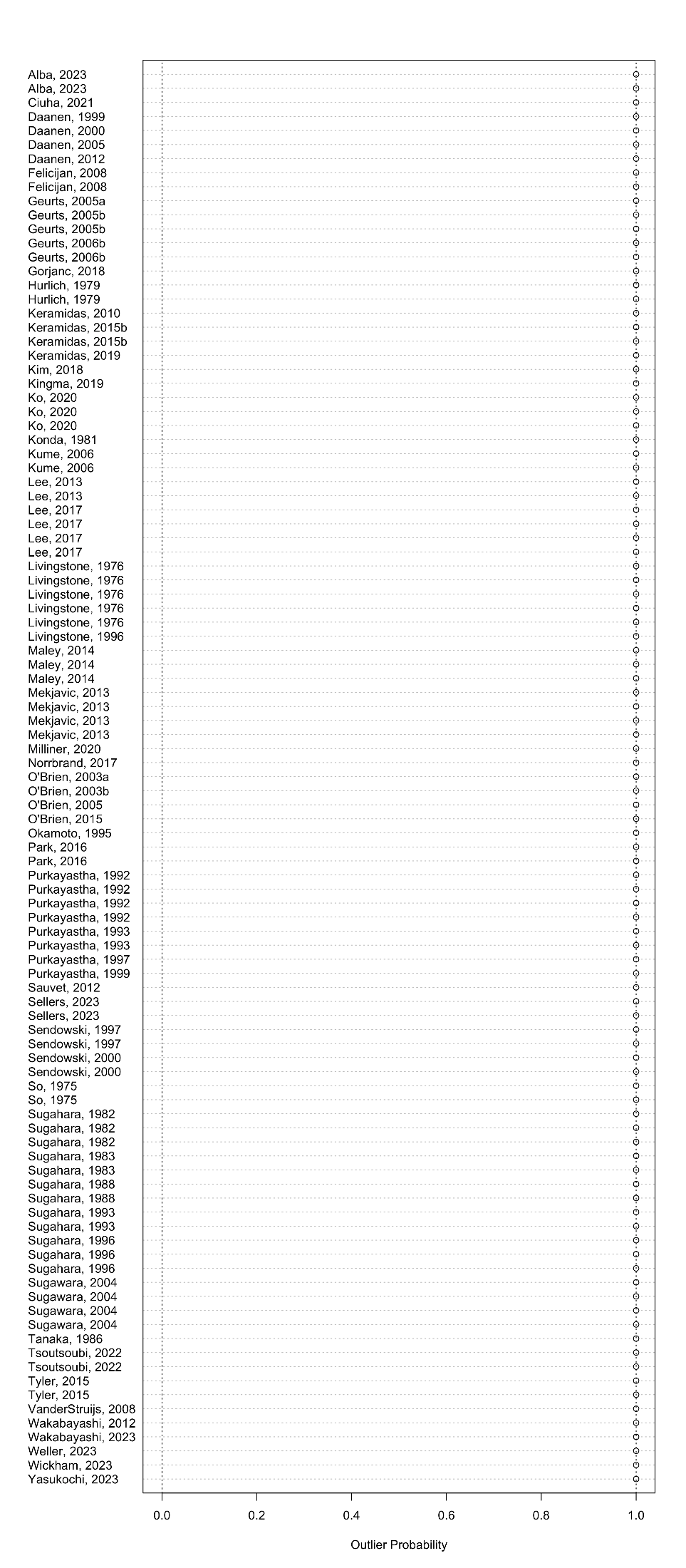


**Supplemental Figure 16:** Probability of Outliers - T_mean_ (°C)


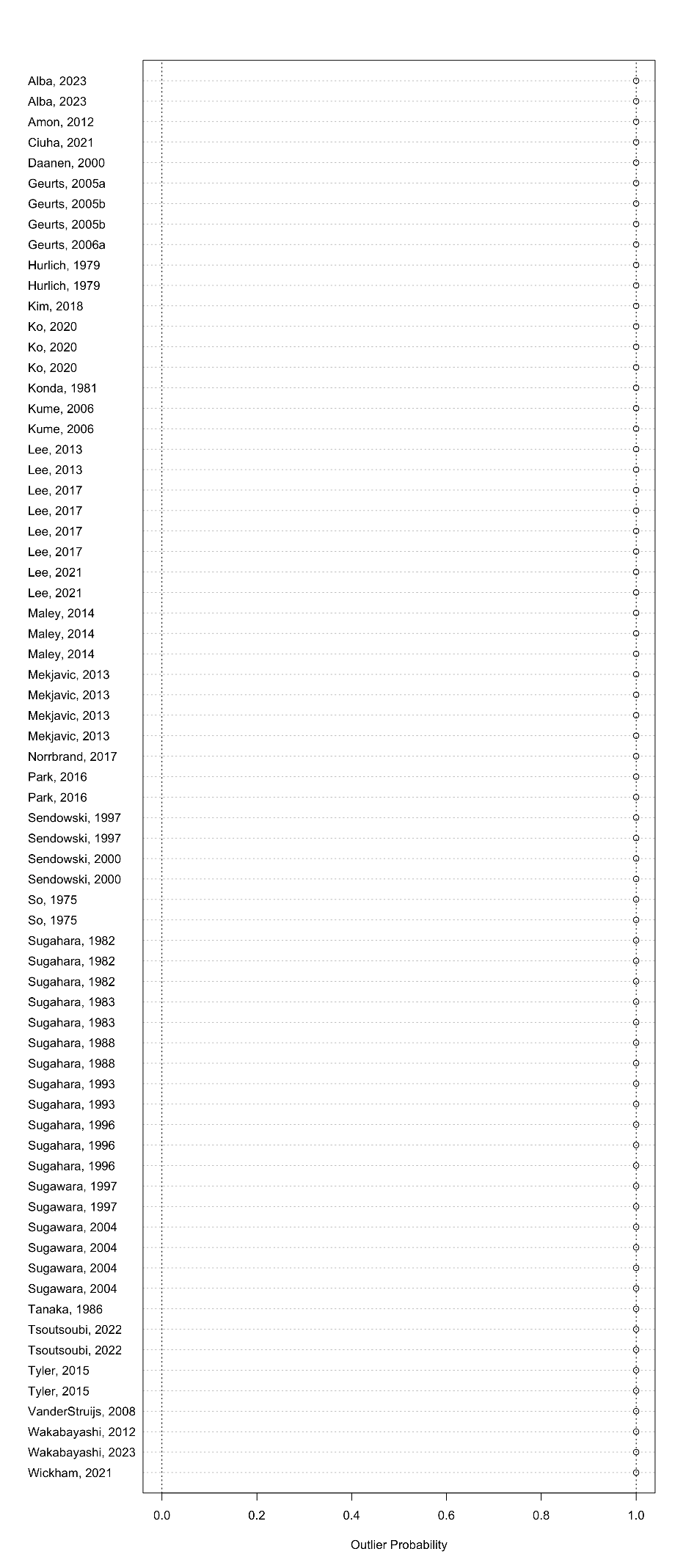


**Supplemental Figure 17:** Probability of Outliers – Amplitude (°C)


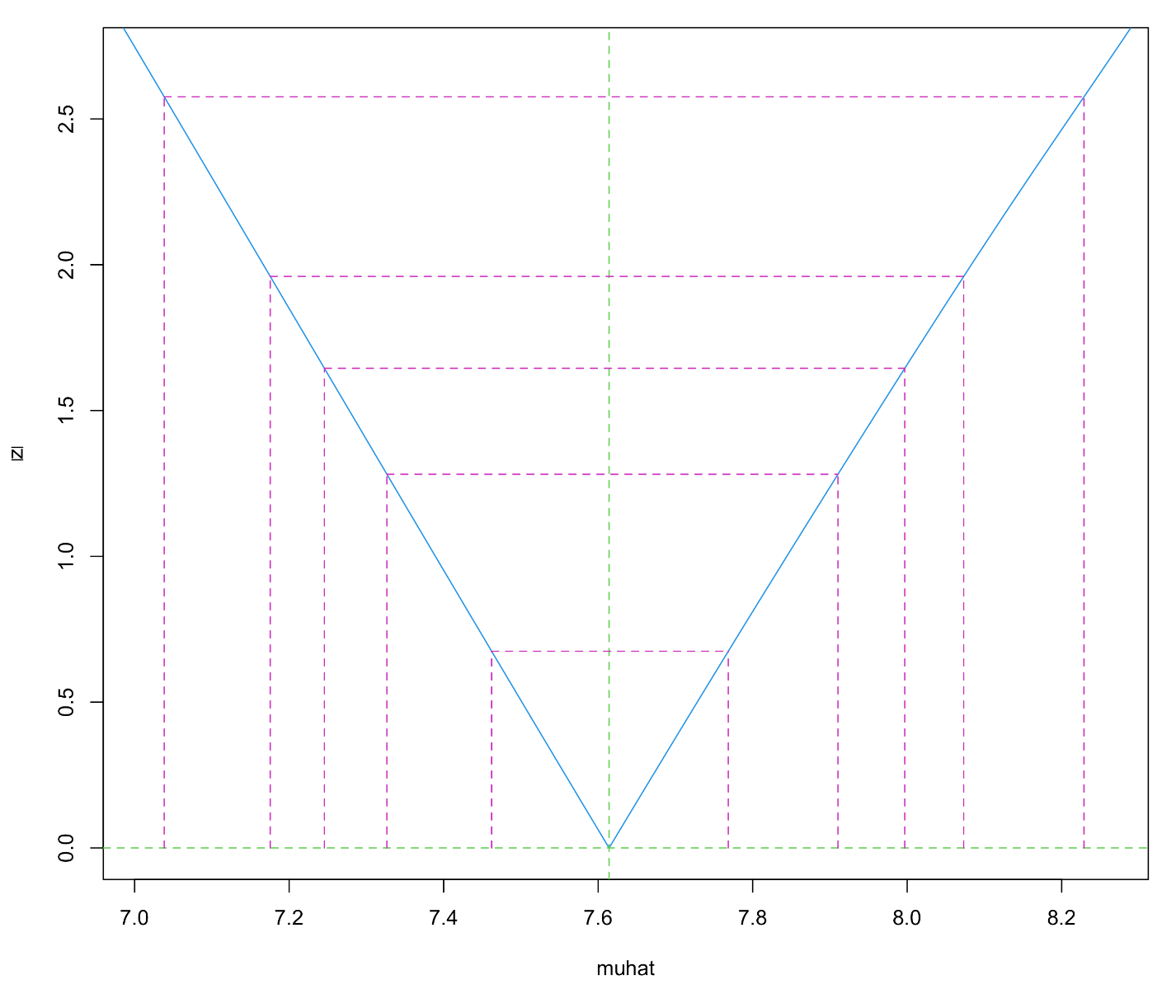


Effect Size

**Supplemental Figure 18:** Effect Size Mixture Distribution – Onset (min)

Onset time in minutes


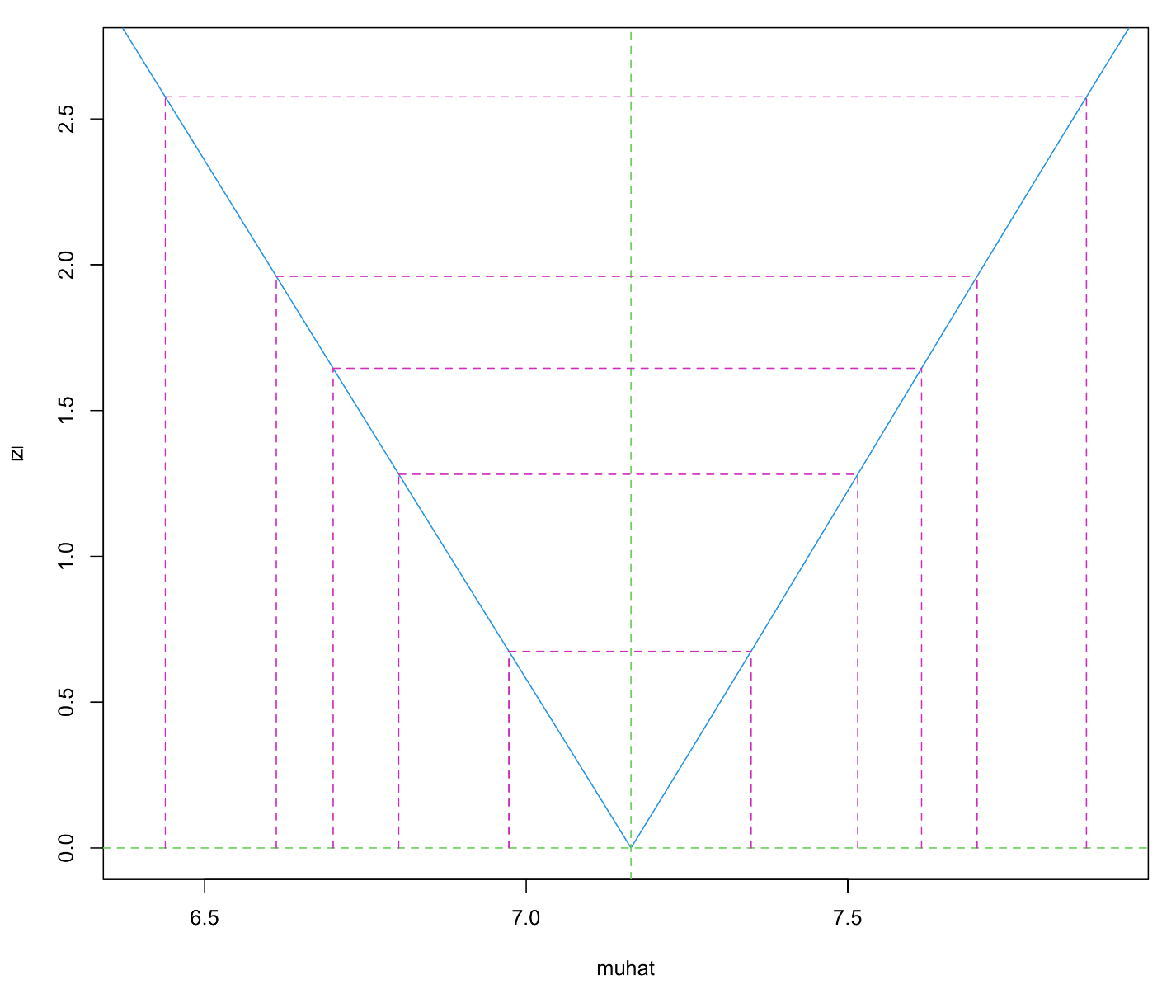


Effect Size

**Supplemental Figure 19:** Effect Size Mixture Distribution - T_min_ (°C)

Temperature in °C


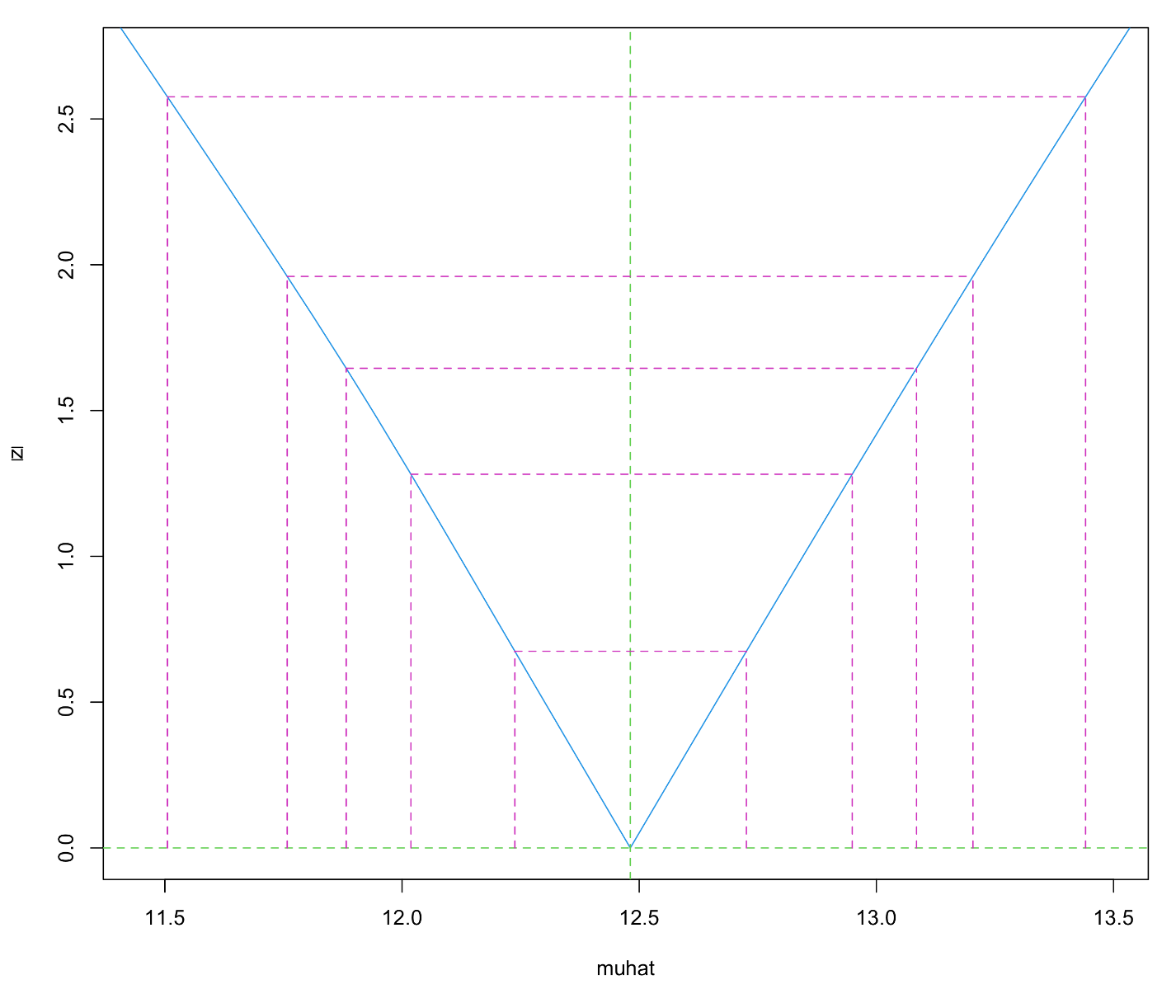


Effect Size

**Supplemental Figure 20:** Effect Size Mixture Distribution - T_max_ (°C)

Temperature in °C


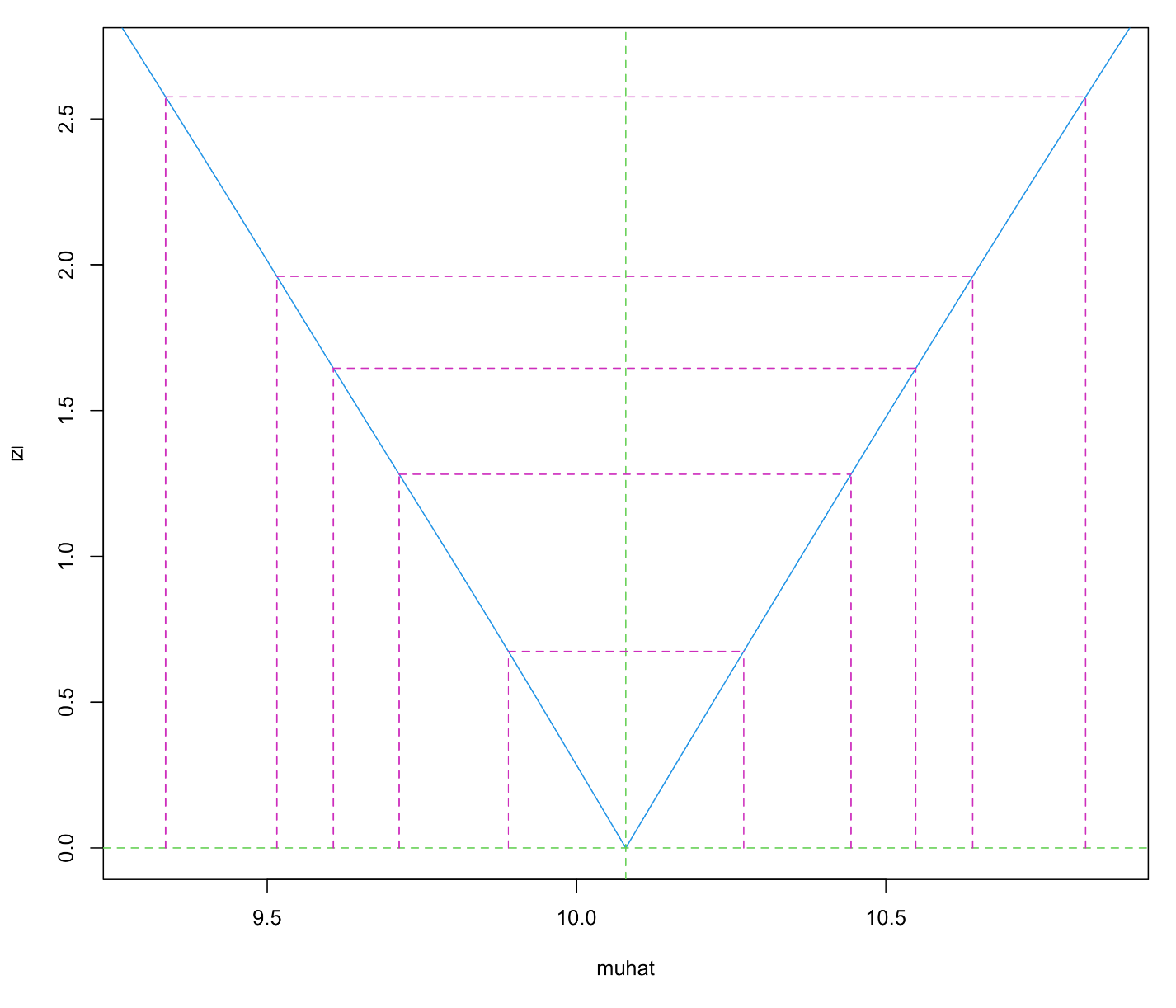


Effect Size

**Supplemental Figure 21:** Effect Size Mixture Distribution - T_mean_ (°C)

Temperature in °C


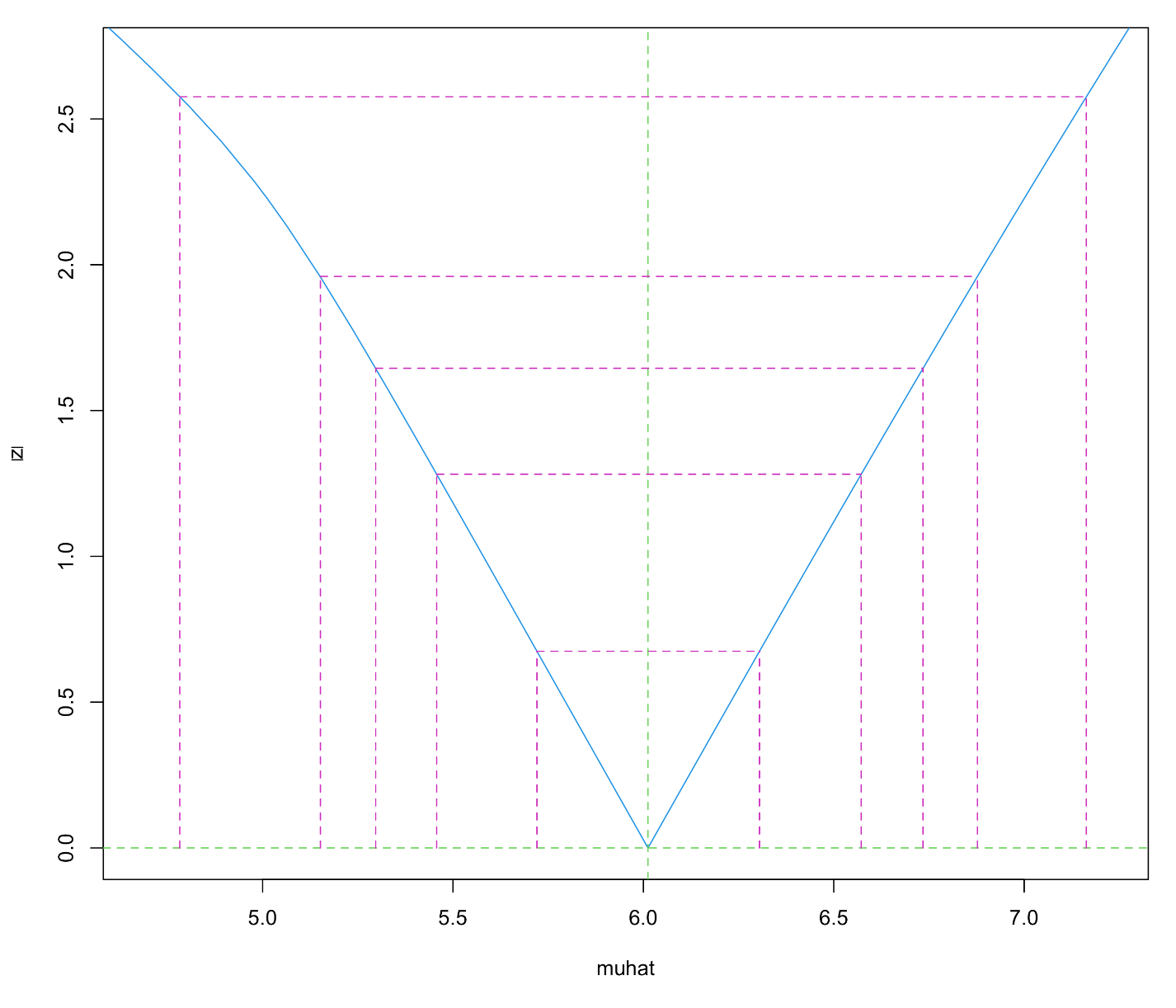


Effect Size

**Supplemental Figure 22:** Effect Size Mixture Distribution – Amplitude (°C)

Temperature in °C


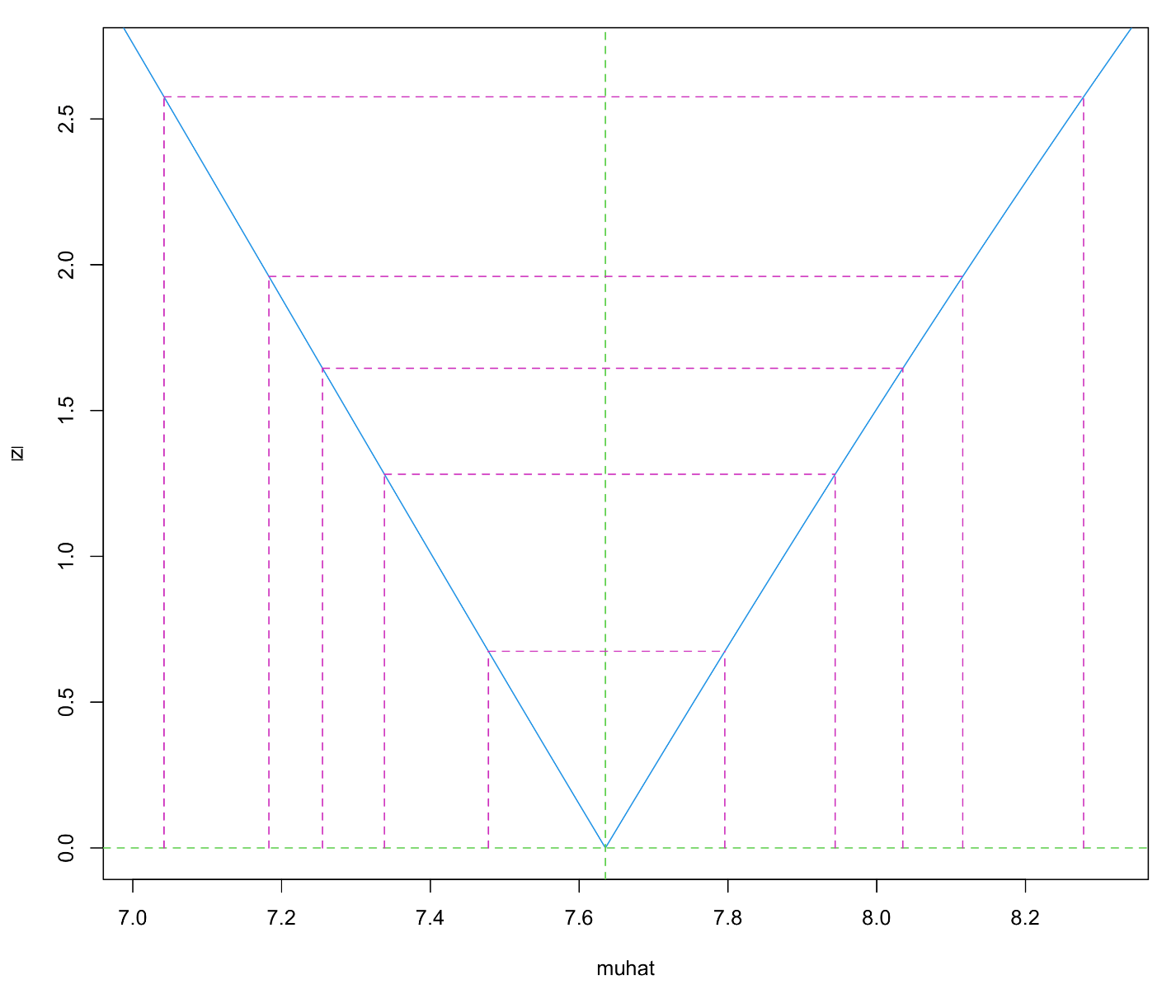


Effect Size

**Supplemental Figure 23:** Effect Size *t* Distribution – Onset (min)

Onset time in minutes


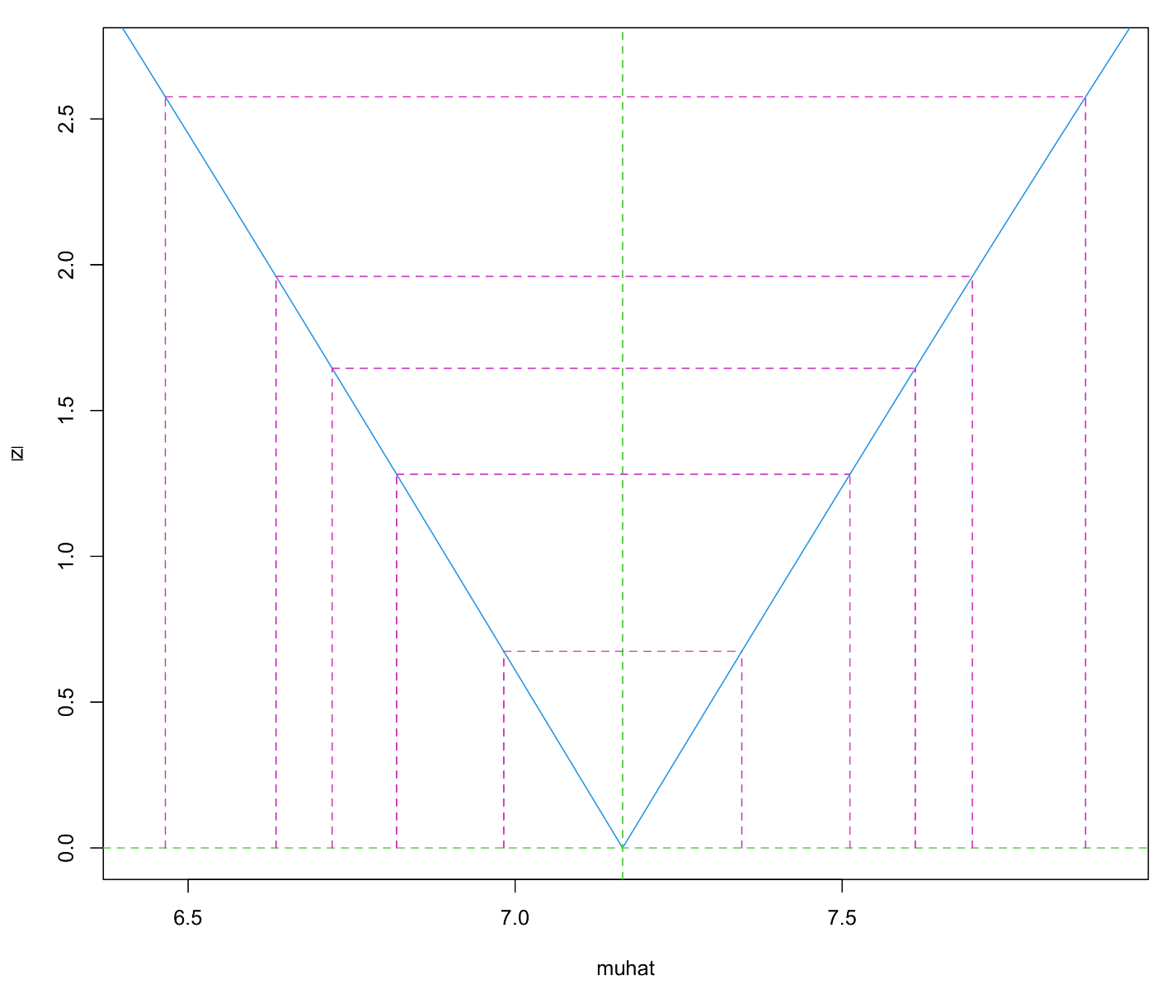


Effect Size

**Supplemental Figure 24:** Effect Size *t* Distribution - T_min_ (°C)

Temperature in °C


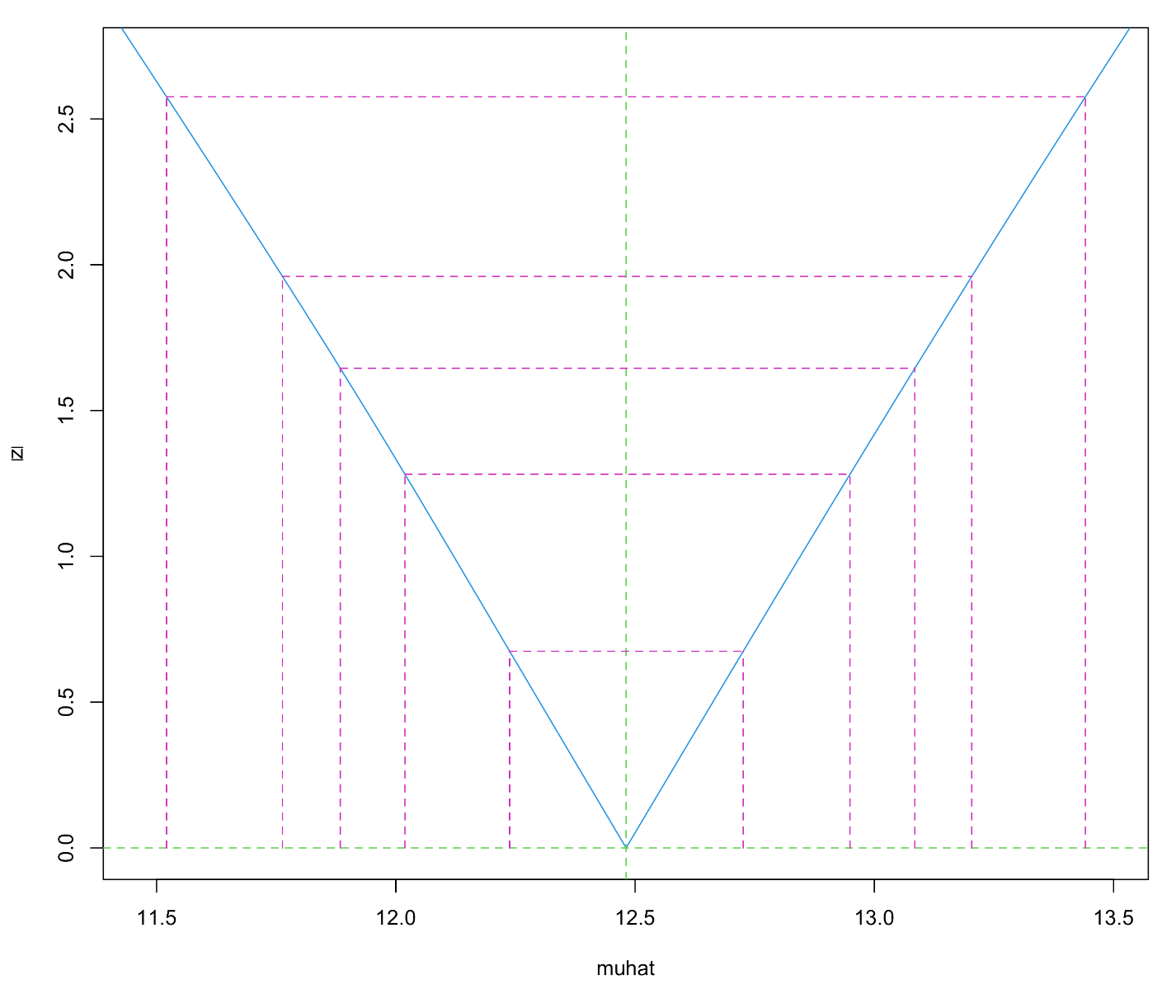


Effect Size

**Supplemental Figure 25:** Effect Size *t* Distribution - T_max_ (°C)

Temperature in °C


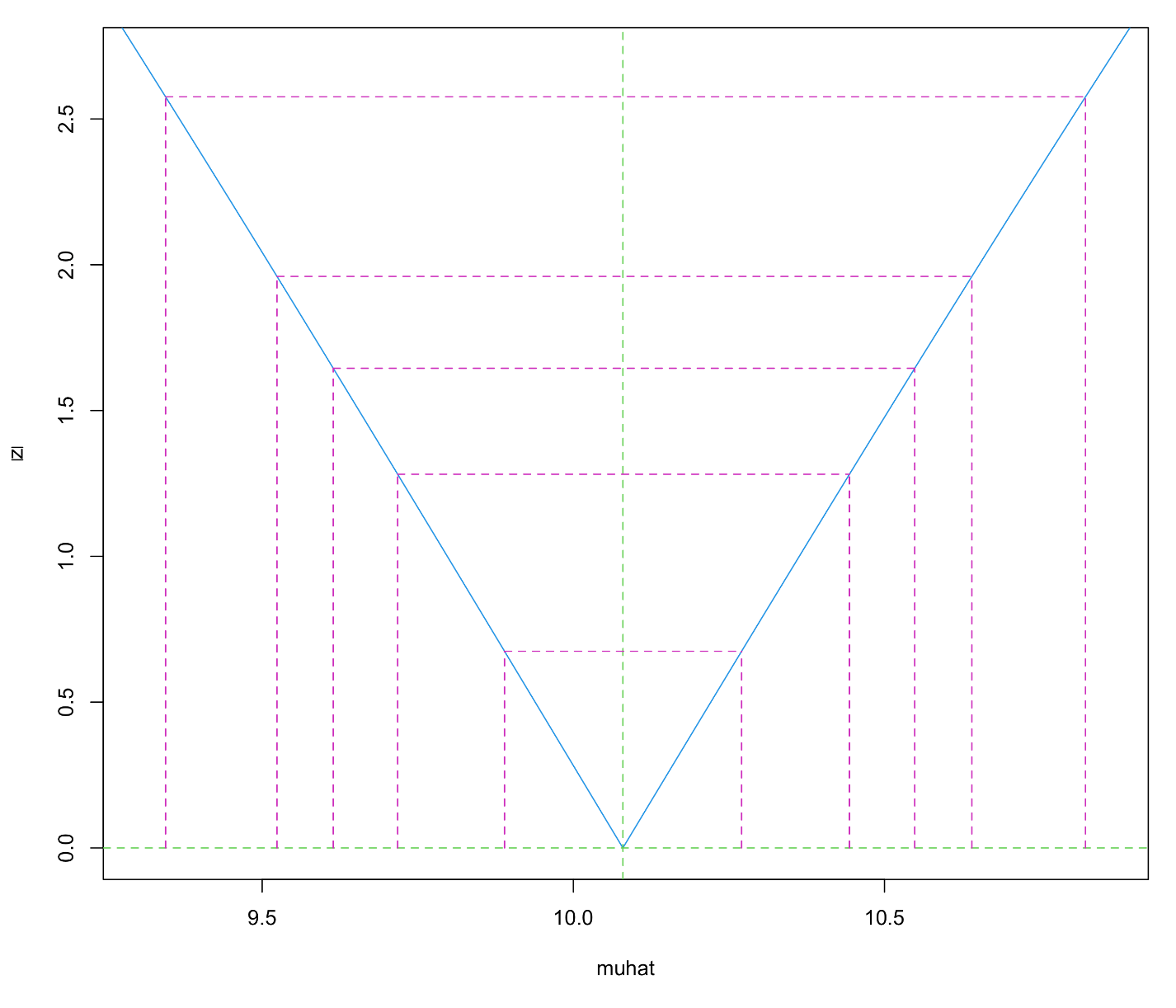


Effect Size

**Supplemental Figure 26:** Effect Size *t* Distribution - T_mean_ (°C)

Temperature in °C


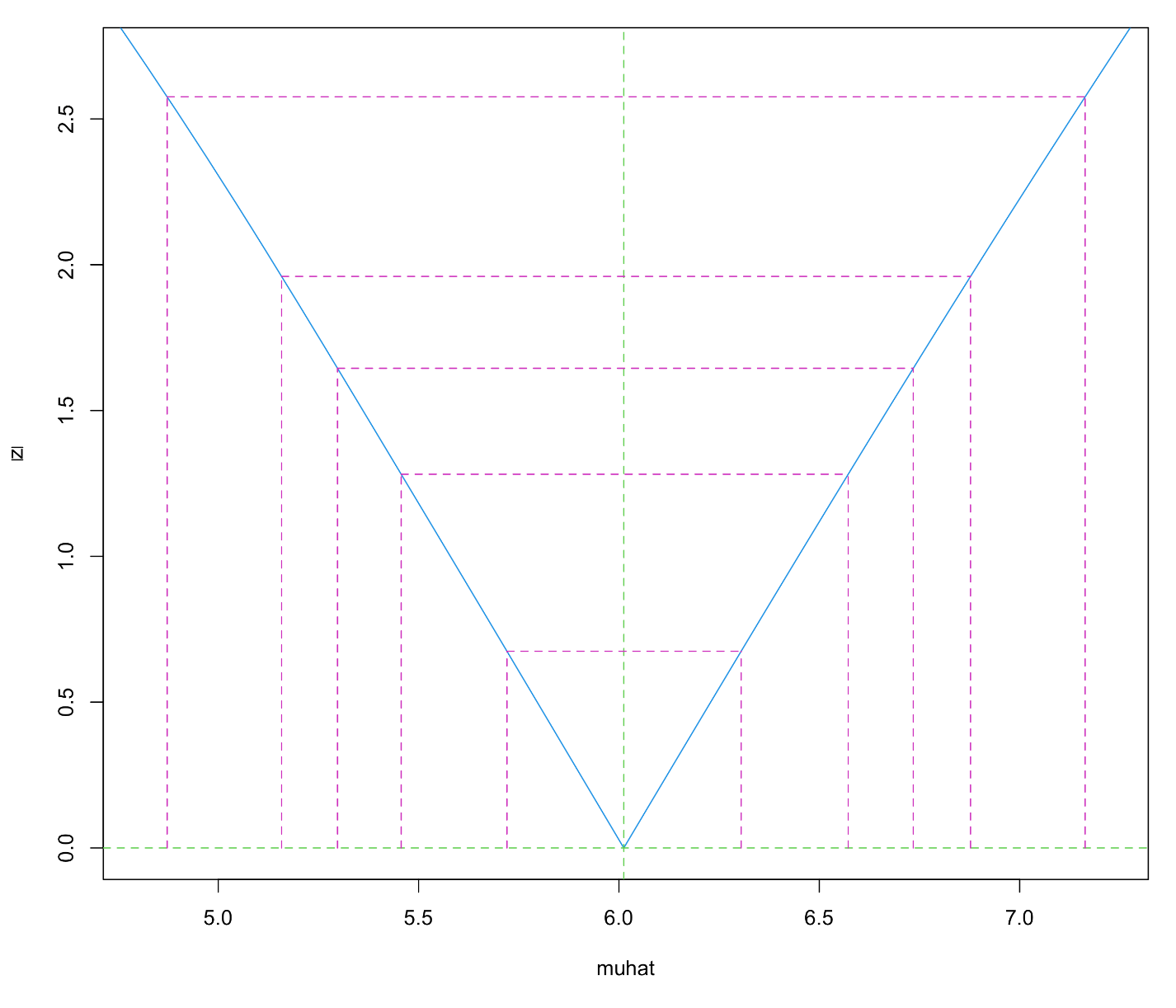


Effect Size

**Supplemental Figure 27:** Effect Size *t* Distribution – Amplitude (°C)

Temperature in °C
